# Identity and mobility through personal ornaments in Upper Paleolithic cantabrian hunter-gatherer societies: Insights from Llonín cave (Asturias, Spain)

**DOI:** 10.1371/journal.pone.0351170

**Published:** 2026-06-08

**Authors:** Daniel Pérez-García de los Salmones, David Cuenca-Solana, Borja González-Rabanal, Ana B. Marín-Arroyo, Elsa Duarte-Matías, Marco de la Rasilla-Vives

**Affiliations:** 1 Instituto Internacional de Investigaciones Prehistóricas de Cantabria, Universidad de Cantabria, Santander, Spain; 2 Centre de Recherche en Archéologie, ArchéoSciences, Histoire (CReAAH), UMR, Rennes, France; 3 Diet and ANcient TEchnology Laboratory (DANTE), Department of Oral and Maxillo-Facial Sciences, Sapienza University of Rome, Rome, Italy; 4 Grupo I+D+i EvoAdapta, Departamento de Ciencias Históricas, Universidad de Cantabria, Santander, Spain; 5 Área de Prehistoria, Departamento de Historia, Facultad de Filosofía y Letras, Universidad de Oviedo, Oviedo, Spain; Universita degli Studi di Ferrara, ITALY

## Abstract

The use of personal ornaments is considered a milestone in human cognitive evolution and the development of complex behavior. The Cantabrian region in the north of the Iberian Peninsula served as a hub of occupation and intergroup contact throughout the Upper Paleolithic, and acted as a climatic refugium during the Last Glacial Maximum (LGM – 25,000–19,000 years ago), constituting a key region for understanding the social and symbolic structures and interactions of hunter-gatherer groups. This study analyses personal ornaments recovered from the Upper Paleolithic stratigraphical sequence at Llonín Cave (Asturias, Spain), spanning from the Upper Solutrean to the Azilian (c. 21.8–11 kya cal BP), that comprises the regional largest and most diverse assemblage. The combination of taxonomic, biometric, taphonomic, and use-wear analyses reveals rarely documented patterns, such as diachronic shifts in the site’s role as an importer and producer of ornaments, the varying social significance of their use, and the strategies for raw material procurement. Furthermore, the study identifies evidence of coastal-inland interactions, allowing the reconstruction of the complete *chaîne opératoire* for ornament manufacture. These findings provide a framework for reinterpreting the use of personal ornaments among Paleolithic hunter-gatherer groups, facilitating critical discussions on key dimensions such as mobility and identity.

## Introduction

Personal ornaments represent the most repetitive and standardized symbolic manifestation of the Paleolithic, and the most widespread in both space and time [1]. The earliest evidence dates back to the Middle Stone Age of Africa and the Middle East [2–8], while in Europe, they are not documented until the Middle to Upper Paleolithic transition [9–15]. During the Upper Paleolithic, there was an intensification in the use of ornaments driven by the demographic increase of human populations, whose interaction or competition led them to develop complex and adaptive communication systems [16]. Personal ornaments have been used to identify and reconstruct cultural ties and standardized ideological systems, characterize the mobility of these groups, and trace medium-to-long-distance exchange networks [17–22]. They have also been used to propose the existence of ethnolinguistic groups based on the techno-typological similarity of ornament assemblages [23,24], as well as to infer cultural continuity as a potential alternative to lithic industry-based studies [25]. Recent studies have integrated these interpretations with social network analysis (SNA) [26] and paleogenetics [24]. Traceology has proven effective in determining manufacturing techniques, modes of suspension, use-wear [5,27–38] and inferring the existence of specialized bead production contexts [32,34]. The social functions of prehistoric ornaments are difficult to trace archaeologically; however, ethnographic and ethnoarchaeological studies focused on their use by contemporary hunter-gatherer groups provide limited hypotheses and analogies based on the archaeological record [39,40].

The Cantabrian region, in northern Iberia, forms a coastal corridor approximately 350 km long and 45 km wide on average, bounded by the Cantabrian Mountain range and the Cantabrian Sea. Factors such as ecological diversity, the significant presence of karst systems, and the role of the mountain range as a natural boundary supported a dense and continuous human occupation throughout the Upper Paleolithic [41–43], also serving as a climatic refuge during the LGM [43–53].

Climatic studies based on marine foraminifera, pollen records, and Greenland ice-core data have enabled the reconstruction of regional environmental conditions from the LGM to the Holocene, revealing a progressive global warming trend punctuated by pronounced climatic instability driven by rapid cooling (HS1, Younger Dryas) and warming events (Bølling–Allerød) [54–58]. These climatic dynamics, together with sea-level rise and the reduction of the coastal platform [59], affected the social and territorial organization of hunter-gatherer groups in the region [43,53].

## Materials and methods

**Fig 1:**
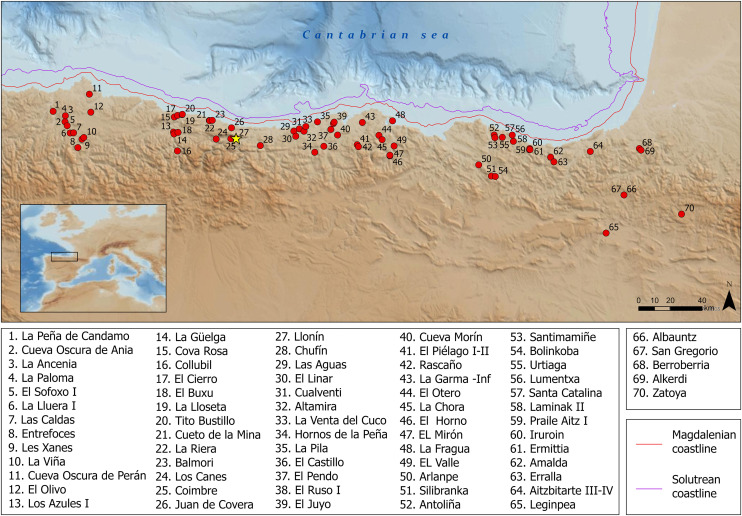
Location of Llonín Cave and other Upper Paleolithic contexts containing personal ornaments from the second half of the Upper Paleolithic in the Cantabrian region and the Upper Ebro Valley. The references consulted for the preparation of this map are liste‌‌d in S2 Table in S1 File.

This paper presents a multidisciplinary analysis of the personal ornaments recovered from the full stratigraphic sequence of Llonín Cave (S1 Text and S6 Fig in S1 File). Llonín Cave, located in the central section of the Cantabrian region (northern Spain) in a narrow valley between the Picos de Europa massif and the coastal platform (Fig 1 and S1 Text in S1 File), constitutes one of the most significant sites of the Cantabrian Paleolithic, featuring a long chrono-stratigraphic sequence spanning from Mousterian occupations to the Bronze Age [60–64], comparable to that of sites such as La Viña, Cueto de la Mina, La Riera, La Garma A or El Mirón. It is also a key site for understanding the transition between the Solutrean and the Magdalenian in the Cantabrian region [64,65]. Moreover, the cave contains an extensive parietal assemblage, with four distinct decorative phases contemporary with the occupations documented in the archaeological levels, as well as numerous superimpositions of graphic motifs [66,67]. These features have enabled a detailed characterization of parietal expression in the western Cantabrian region and its relationship with material culture. The assemblage originates from the site’s four main excavation areas, Vestíbulo, Galería, Cono Anterior, and Cono Posterior (S2-S6 Figs in S1 File), and includes levels associated with the Upper Solutrean (c. 23.5–22 cal kya), Badegoulian (c. 22–21 cal BP kya), Magdalenian (c. 21–14 cal BP kya), and Azilian (c. 13.8–11 cal kya) periods [60–64], while no Gravettian ornaments have been documented (S1 Text and S1 Table in S1 File). Materials were recovered through excavation in 1 m² grid units subdivided into nine sub-squares. Excavation proceeded by removing spits of 2–3 cm, always following the stratigraphic structure and dip of each archaeological layer. Finds were three-dimensionally recorded, and the sediment was washed and subjected to flotation. Screening and sorting were carried out using two sieves of 2.38 and 1.41 mm mesh size, respectively, in order to ensure the recovery of small-sized materials. Regarding the excavated areas, between 1984 and 1999 a total of 4 m² were excavated in the Galería sector, with an average depth of 1.5 m; 8 m² in the Vestíbulo, with an average depth of 1.3 m; 14.5 m² in the Cono Anterior, with an average depth of 2.1 m; and 36 m² in the Cono Posterior, with an average depth of 1 m.

According to the archaeological evidence, the site has been interpreted as a complex residential site during the Upper Solutrean, and as having served more specialized purposes during the Badegoulian, such as hide or food processing [61,64] (S1 Text in S1 File). The evidence from portable and parietal art, as well as osseous industry, reveals the importance of symbolic practices in the Magdalenian levels, particularly during the Middle Magdalenian, which shows high intensity and variability, while the Upper Magdalenian is represented by a more limited and less diverse material record [68].

### Taxonomic and biometric analyses

The taxonomic identification of the specimens is based on the comparative collection of malacofauna, ichthyofauna, and macromammal osteology held at the Instituto Internacional de Investigaciones Prehistóricas de Cantabria (IIIPC-Universidad de Cantabria) and the mammal osteological collection housed at the EvoAdapta Group (Universidad de Cantabria) facilities. Additionally, the World Register of Marine Species [69] website along specialized literature [70–76] was used. Raw materials were classified according, first, to material type and, second, to the Linnaean taxonomic framework, establishing a general categorization at the class level and a more specific categorization at the family, genus, or species level, depending on the degree of precision permitted by each specimen. Ornaments manufactured from vestigial deer canines were classified based on laterality, age, and sex, following qualitative and quantitative criteria established by previous studies [76,77], including occlusal wear stage, apex root opening, and canine morphology. Red deer (*Cervus elaphus*) exhibits a certain degree of sexual dimorphism in its vestigial canines, which allows for an initial sex attribution that can later be verified through biometric results. A positive correlation exists between root width and thickness measurements in both male (p = 0.03) and female (p = 0.027) canines, underscoring their applicability in sex estimation (S8 Fig in S1 File). Projected on a scatterplot, the root width-to-root thickness ratio generates distinct clusters, enabling the distinction between males and females. Statistical correlations and comparisons between root width and root thickness by sex were undertaken using the *ggplot2* package from R software [78,79]. A Shapiro–Wilk test was used to assess whether both populations were normally distributed (p-value >0.05). Depending on the result, Pearson/Spearman correlation tests were used to analyze significant relationships. Later, a Wilcoxon-Mann-Whitney U test with a post-hoc Holm-Bonferroni correction was conducted to know if they were significantly different (p-value of <0.05).

Biometric analysis of shell ornaments was conducted using metric variables defined by Álvarez-Fernández [80] for bivalves, scaphopods, and coiled gastropods, and by Gutiérrez-Zugasti [81] for uncoiled gastropods (S7 Fig in S1 File). Fragmented or incomplete shells were excluded from biometric analysis, as such conditions could distort statistical interpretations of the results. Additionally, two modern reference collections of *Littorina obtusata/fabalis* and *Trivia* sp. from the beaches of Ballenera (Cangas, Galicia, northern Spain) and Area Grande (A Guarda, Galicia, northern Spain) were measured to establish a comparative framework with the archaeological shells (S5 Data). General measurements of length and width were taken of complete bone and teeth elements, except for specimens exhibiting circular morphology, such as fish vertebrae, for which the diameter was measured.

### Taphonomic analysis

Taphonomic analysis leads to identifying the limitations that taphonomic alterations may pose to the microscopic study of the material and to assessing the overall preservation state of the assemblage, in relation to the biostratinomic and diagenetic processes it was subjected to. It also enables the identification of alterations affecting the specimens as a result of their interaction with or manipulation by humans, as well as processes affecting organisms before or after death, which in turn allow inferences to be made regarding the modes of acquisition of these specimens.

The study followed the taphonomic categories defined by Gutiérrez-Zugasti [81] for mollusks, and those defined by Behrensmeyer [82], Fernández-Jalvo and Andrews [83], and Lyman [84] for dental and osseous elements. Taphonomic processes were grouped into three categories: pre-collection alterations, anthropogenic alterations, and post-depositional alterations. Fragmentation, in turn, is considered a cross-cutting taphonomic process affecting all three categories. The first category includes surface abrasion produced by marine action, encrustation by other mollusks, and shell perforation by lithophagous organisms. The differentiation between anthropogenic perforations and those produced naturally by lithophagous organisms is based on morphological and morphometric criteria. These organisms produce highly regular and homogeneous circular perforations, with cylindrical or conical cross-sections, typically located on the external surface of the shells, through the use of a rasping organ known as the radula, in order to access the soft tissues of the prey or to obtain minerals from the shell itself [31,81]. Furthermore, such perforations are generally smaller, not exceeding 1.5 mm in diameter.

Anthropogenic processes include cut marks, decoration, thermal alteration, ochre staining—whether deliberate or accidental—and anthropogenic perforation. Decorative marks can be distinguished from technological marks or cut marks by their tendency to occur in organized patterns, such as short parallel or concentric lines, produced through deep grooves that require a sawing action or repeated bidirectional incision, generally using unretouched tools. In the case of teeth, these marks tend to be located on the occlusal surface [76]. Cut marks, by contrast, tend to appear in a more irregular or overlapping arrangement, often in small clusters, and are characterized by superficial or shallow grooves; in teeth, they are typically concentrated around the root or at the junction between the root and the enamel [76,82–84].

Post-depositional alterations encompass all processes resulting from the exposure of objects to natural, karstic, and sedimentary dynamics, including manganese staining, concretion, dissolution, biodegradation, weathering, and sediment infilling. For detailed definitions of the abovementioned processes, see [81–84].

### Morphometric and use wear analysis

Traceological analysis involves the study of the location, morphology, and morphometry of perforations, as well as the interpretation of drilling techniques and use-wear marks resulting from both manufacture and suspension as ornaments. The location of perforations in coiled gastropods follows the nomenclature proposed by Taborin [27], while uncoiled gastropods and bivalves were classified according to custom criteria based on archaeological material. The morphology of the perforations combines the categories proposed by Tatá et al. [28] and Álvarez-Fernández [80] (S8 Fig in S1 File). Morphometric analysis was carried out by measuring the maximum width of each perforation. Fractured perforations that preserved less than half their perimeter or whose widest point could not be estimated were not measured.

The technological and use wear analyses of personal ornaments is based on extensive published literature regarding manufacture techniques [28,85,86], use-wear [33,87,88] and both [5,12,27,29,32,35,89,90]; as well as on comparative experimental collections developed by the Bioarchaeology, Paleoclimate, and Social Transformations in Prehistory research group (IIIPC), which includes 260 experiments involving 7 perforation techniques applied to 14 mollusk, mammal and fish taxa, and 373 elements from 6 taxa subjected to suspension using various systems and materials (S11 Fig in S2 File).

Taphonomic and traceological analyses of manufacturing and use-wear traces were carried out using a Leica S8APO stereomicroscope with a Leica MEB115 ring light (magnification 10–80×) and a Leica DM2500M microscope (magnification 50–200×). Microscopic images from both devices were captured with a Leica K3C camera using LAS X software. Macroscopic images were taken with a Nikon D750 camera and an AF-S Micro NIKKOR 60 mm 1:2.8G ED lens.

## Results

The analyzed assemblage consists of 271 items (S1-S4 Data and S9 Fig), of which 180 (66,42%) are malacofaunal remains, 80 (29.52%) are mammalian teeth, 10 (3.69%) are bone fragments, and 1 (0.37%) is a fossil. The majority of items (n = 191; 70.48%) come from the Cono Anterior, while the rest are distributed among the Vestíbulo (n = 12; 4.43%), Galería (n = 62; 22.88%), and Cono Posterior (n = 6; 2.21%). Chronologically, they are attributed to the Upper Solutrean (n = 105; 38.75%), Badegoulian (n = 22; 8.12%), Middle Magdalenian (n = 92; 33.95%), Upper Magdalenian (n = 14; 5.17%), and Azilian (n = 3; 1.11%). Finally, 34 items (12.55%) lack chronological attribution as they come from fall of stratigraphic section, profile cleaning, and unclear stratigraphic correspondence. The highest artefact densities are recorded in the Solutrean level of Galería (38.89 items/m³) and in the Middle Magdalenian level of Cono Anterior (28.67 items/m³) (S3 Table in S1 File). It is worth noting that the analyzed assemblage (n = 271) includes unperforated mollusks without nutritional value, which may represent unprocessed raw material or discarded ornaments during manufacturing [28,34], as well as all unperforated atrophic deer canines, given the unusually high number of such specimens and their potential implications for ornament production.

### Taxonomic analysis

**Fig 2:**
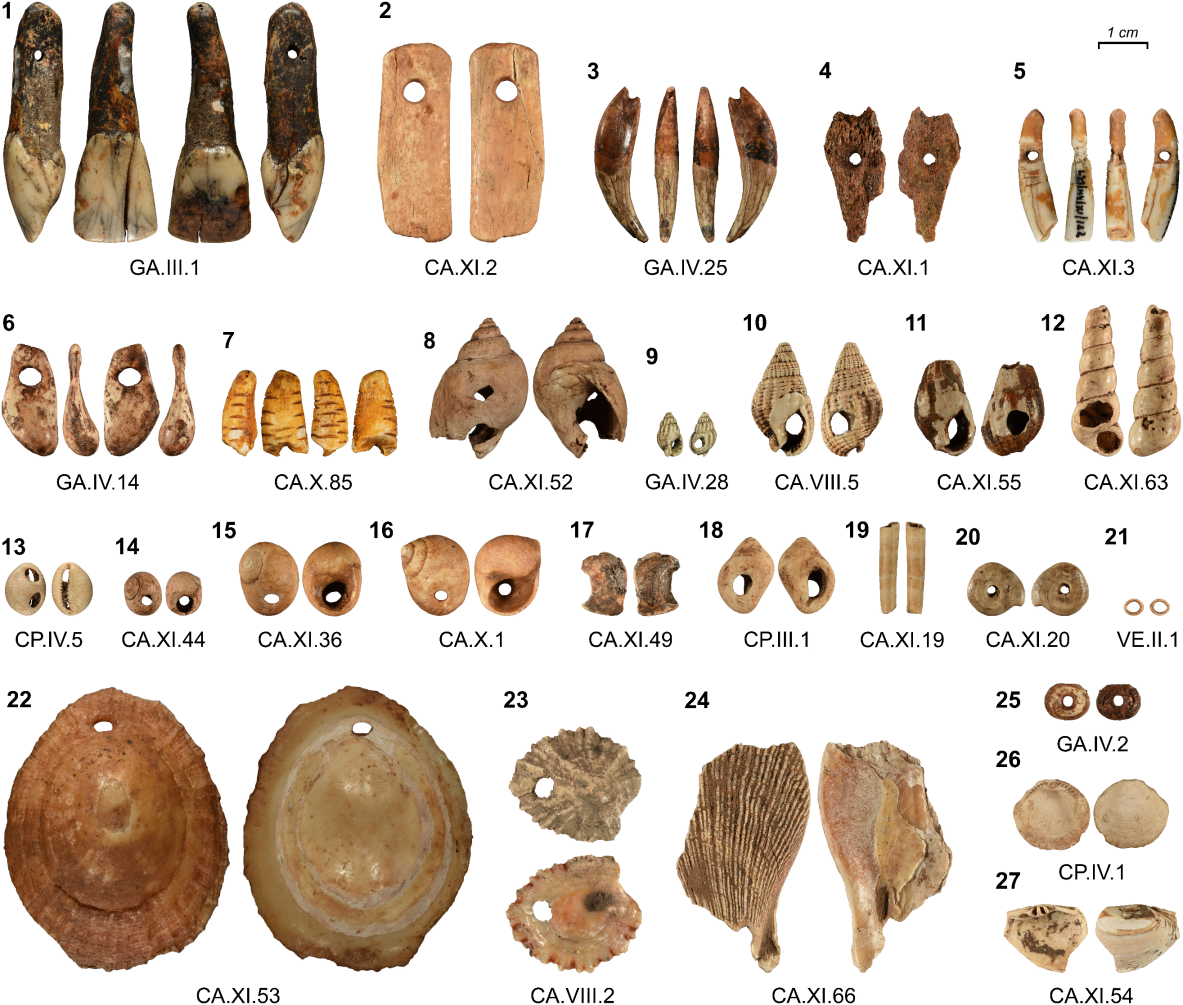
Taxonomic representation of Llonín cave personal ornaments. (1) *Bos/Bison* sp. (2) *Equus ferus* (3) *Vulpes* sp. (4) *Mammalia* (5) *Capra pyrenaica* (6) *Cervus elaphus* (7) *Canis lupus* (8) *Tritia mutabilis* (9) *Tritia incrassata* (10) *Tritia reticulata* (11) *Tritia* sp. (12) *Turritellinella tricarinata* (13) *Trivia* sp. (14) *Littorina saxatilis* (15) *Littorina obtusata/fabalis* (16) *Littorina littorea* (17) *Littorina* sp. (18) *Nucella lapillus* (19) *Antalis* sp. (20) *Rotularia* sp*.* (21) *Pisces* (22) *Patella vulgata* (23) *Patella depressa* (24) *Chlamys islandica* (25) *Salmo* sp*.* (26) *Glycymeris* sp*.* (27) *Veneridae.*

**Fig 3:**
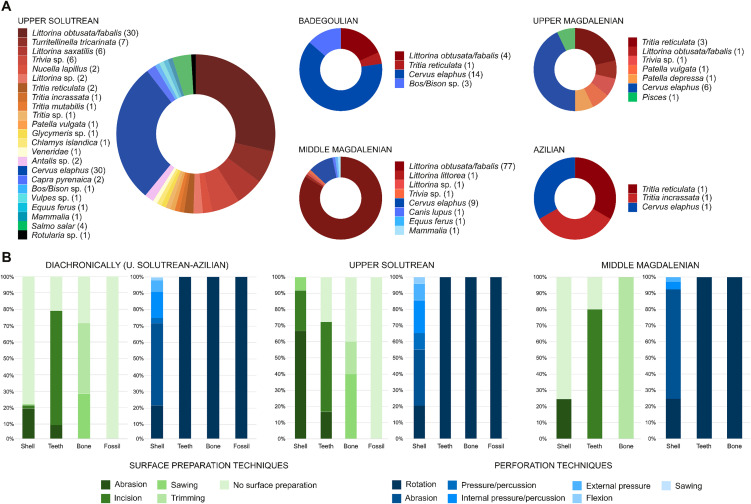
Taxonomic and technologic analysis of the assemblage. **(A)** Proportions in which the different taxa appear diachronically in the assemblage. **(B)** Proportions in which the documented manufacturing techniques occur by raw material diachronically, during the Upper Solutrean and during the Middle Magdalenian.

The taxonomic analysis of the assemblage has documented the presence of ornaments made from raw materials of both aquatic (marine and fluvial) and terrestrial origin, belonging to 17 different genera, and 15 species (Figs 2 and 3). A total of 239 specimens were identified at the species level, 28 at the genus level, 1 at the family level, and three could only be assigned to the class level, comprising two specimens from *Mammalia* and one from *Pisces* (Table 1).

**Table 1:** Taxonomic analysis of the assemblage. The presence of the various taxa is shown here according to the different stratigraphic and chronological attributions of Llonín Cave.

	Upper Solutrean					Badegoulian		Undetermined Magdalenian	Middle Magdalenian			Upper Magdalenian			Azilian	*Undetermined chronology*
	CA.XII	CA.XI	GA.IV	VE.IV	CP.IV	GA.III	VE.III	CP.III	CA.X	GA.II	VE.IIb	VE.II-IIa	CA.IX	CA.VIII	CA.VII	
***Mammalia***																
*C. elaphus (canine)*	1	14	13	1		13	1		4	5		3		3	1	12
*C. elaphus (scapula)*			1													
*Bos/Bison* sp*. (incisor)*	1					3										
*C. pyrenaica (incisor)*		2														1
*E. ferus (hyoid)*		1									1					
*Vulpes* sp*. (canine)*			1													
*C. lupus (canine)*									1							
*Mammalia undet.*		1							1							
***Pisces***																
*Salmo* sp*. (vertebra)*			4													
*Pisces (vertebra)*												1				
***Gastropoda***																
*L. obtusata/fabalis*		21	8	1			4		77					1		16
*L. saxatilis*		6														
*L. littorea*									1							
*Littorina* sp.		2							1							
*N. lapillus*		2						1								
*T. mutabilis*		1														1
*T. reticulata*		1			1	1								3	1	1
*T. incrassata*			1												1	
*Tritia* sp.					1											
*T. tricarinata*		7														
*Trivia* sp.		4			2				1				1			3
*P. vulgata*		1											1			
*P. depressa*														1		
***Bivalvia***																
*Glycymeris* sp.					1											
*C. islandica*		1														
*Veneridae*		1														
***Scaphopoda***																
*Antalis* sp.	1	1														
***Polychaeta***																
*Rotularia* sp.		1														
***Tooth***	33					17			10			6			1	13
***Bone***	7								2			1				
***Shell***	64					5		1	80			7			2	21
***Fossil***	1															
***TOTAL***	**105**					**22**		**1**	**92**			**14**			**3**	**34**

The marine record is predominantly composed of mollusks from the *Gastropoda* class (97.22%), being the *Littorina* genus –primarily represented by *L. obtusata/fabalis*– the most abundant taxon. No distinction has been made between these two species, as they are morphologically indistinguishable except for their reproductive organs, which are not preserved in the archaeological record [91,92]. The same applies to the *Trivia monacha* and *Trivia arctica* shells, classified as *Trivia* sp., since they can only be differentiated by their colour pattern, usually lost in archaeological specimens [93]. To a lesser extent, numerous genera gastropods such as *Tritia*, *Turritellinella*, *Nucella* and *Patella* have been documented. Bivalves, following the cantabrian ornamental patterns, are much scarcer, with three specimens corresponding to *Chlamys islandica*, *Glycymeris* sp. and a *Veneridae* family shell. Lastly, *Scaphopoda* class includes 2 fragments of *Antalis* sp. tusk shells. Additional information regarding the ecology of this species has been included in S2 Text in S1 File.

The fluvial-marine fauna record consists of four salmonid vertebrae and one belonging to an undetermined smaller *pisces* class individual. Terrestrial fauna is dominated by atrophic canines of red deer (*Cervus elaphus*) (83.53%), although ornaments made from the bones and teeth of other mammals are also present, including bovine incisors, horse (*Equus ferus*) hyoid bones, ibex (*Capra pyrenaica*) incisors and premolars, and fox (*Vulpes* sp.) and wolf (*Canis lupus*) canines. Isolated bovine incisors cannot be identified to species level [94]; therefore, the designation *Bos/Bison* sp. has been used. The results of the detailed study of teeth and bone ornaments are presented in S2 and S3 Data. Given the absence of paired canines, a minimum number of 71 deer specimens has been determined (MNI = 71). Lastly, the fossil specimen consists of a perforated tube-dwelling annelid (*Polychaeta* class) belonging to the genus *Rotularia*.

### Biometric analysis

**Fig 4:**
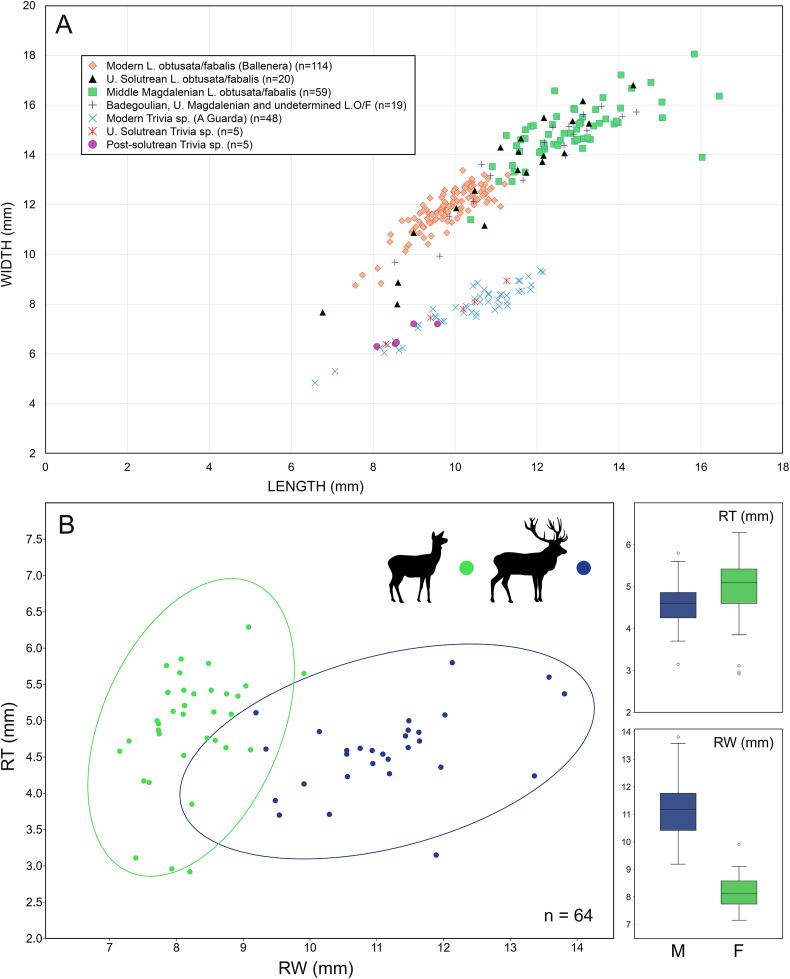
Biometric analyses. **(A)** Scatter plot showing the width to length relation of Llonín cave and modern *Littorina obtusata/fabalis* and *Trivia* sp. shells. **(B)** Scatter plot of root thickness (RT) and root width (RW) of male and female red deer canines, and separate boxplots for RT and RW distributions. A Wilcoxon-Mann-Whitney U test was carried out, with a post-hoc Holm-Bonferroni correction, showing that both groups are significantly different (RT p-value = 1,224e-11; RW p-value = 0.009604).

Measurements were taken from 211 out of the 271 specimens that make up the assemblage (S7 Fig in S1 File and S1-S4 Data). Measurements from archaeological *L. obtusata/fabalis* and *Trivia* sp shells have been plotted alongside specimens from modern collections obtained from the Galician beaches of Ballenera and Area Grande (Galicia, northern Spain) (S5 Data and Fig 4). Results show greater heterogeneity regarding size among *Littorina* shells in Upper Solutrean levels, while during the Middle Magdalenian shells tend to cluster around larger sizes, compared to modern collections and the solutrean record. *Trivia* shell sizes exhibit no statistically significant variation across different chronologies or in comparison to modern specimens. Biometric analysis of red deer canines has enabled the verification of sex attribution based on morphological criteria by comparing it with the results of root width-to-root thickness ratios. Overall, no significant preference for one sex over the other is observed in the *Cervus elaphus* sample from Llonín at any point of the sequence (Fig 4).

### Taphonomic analysis

**Fig 5:**
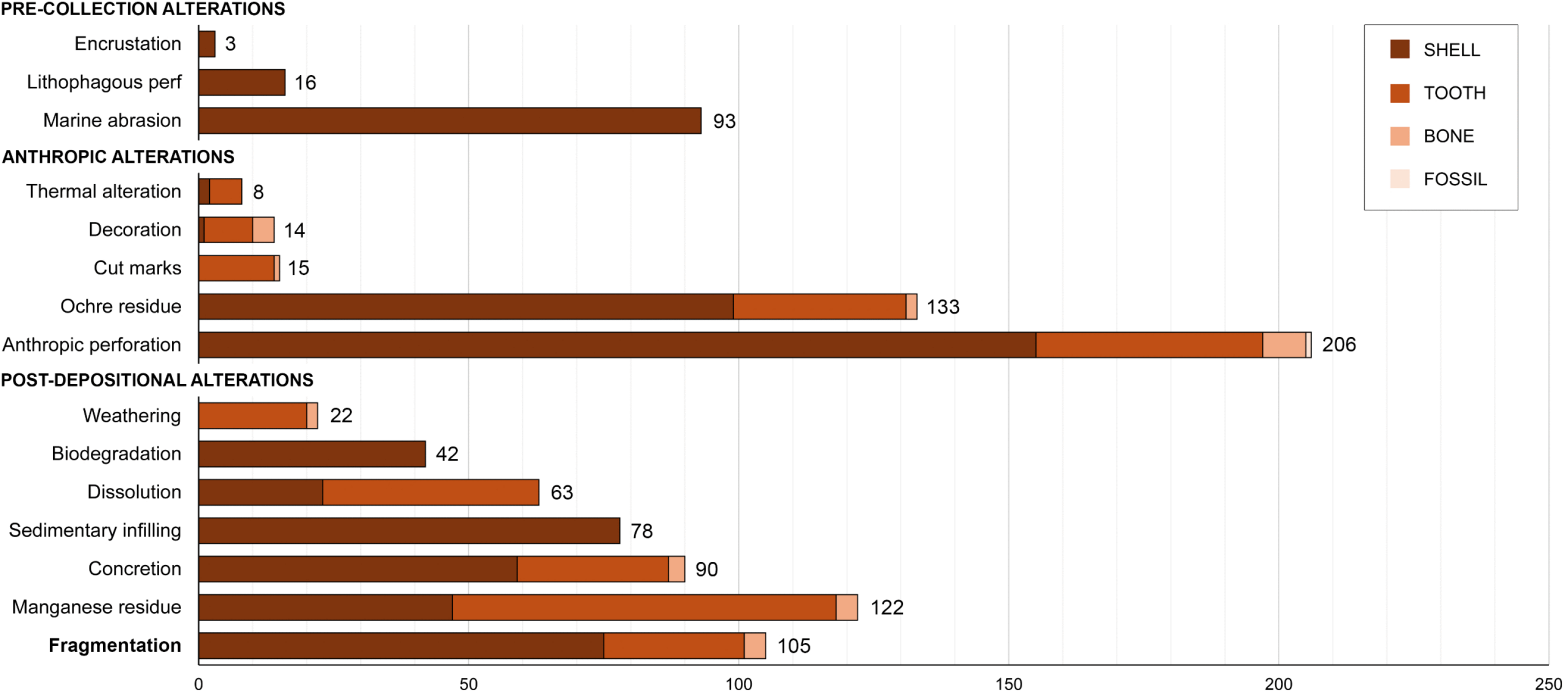
Taphonomic processes and their abundance in the assemblage.

**Fig 6:**
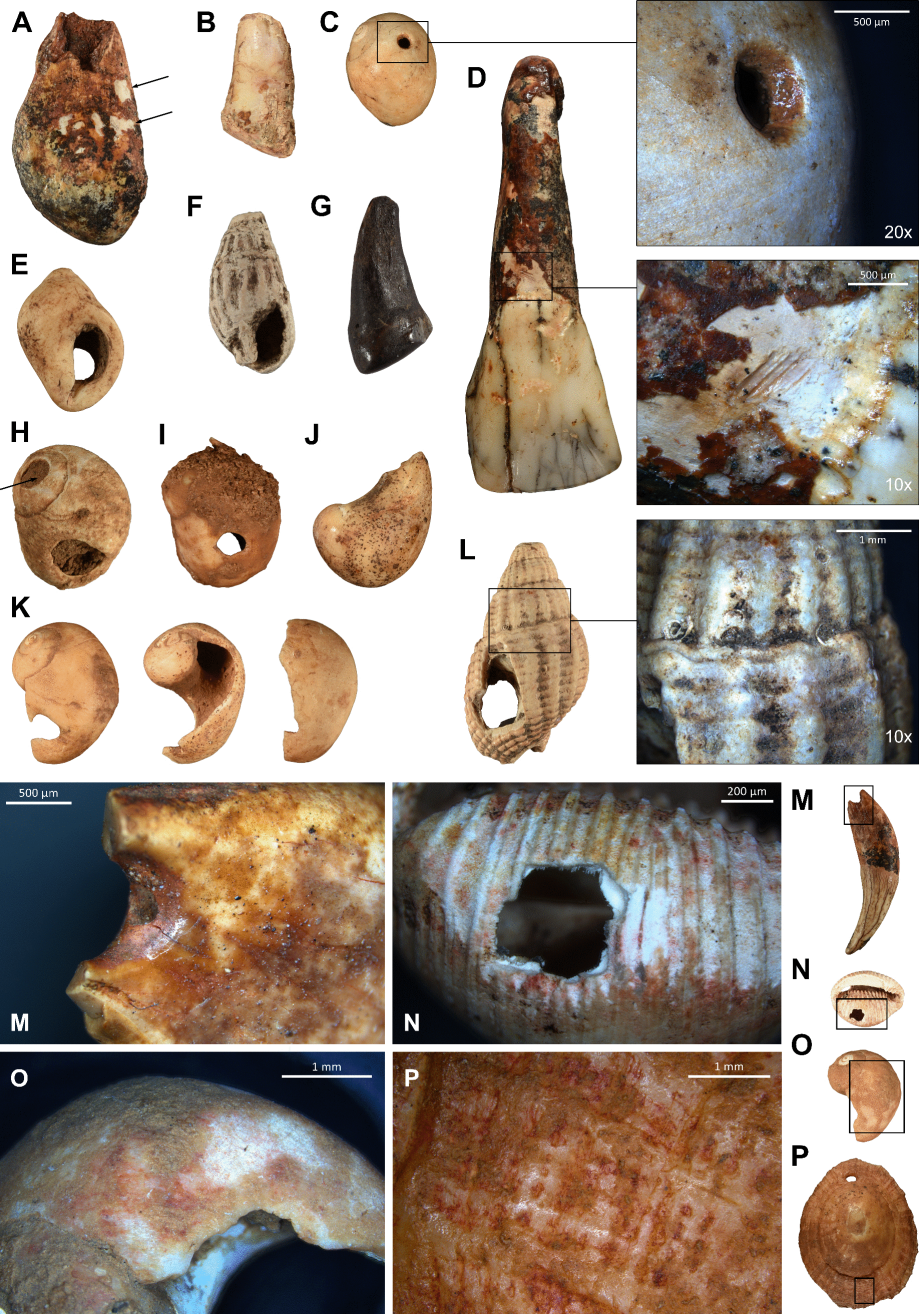
Evidence of the different taphonomic processes present in the assemblage. **A.** Dissolution, **B.** Weathering, **C.** Lithophagous perforation, **D.** Cut marks, **E.** Marine abrasion, **F.** Biodegradation, **G.** Thermal alteration, **H.** Sedimentary infilling, **I.** Concretion, **J.** Manganese residue, **K.** Fragmentation, **L.** Encrustation, M-P. Ochre residue.

We have identified 16 taphonomic processes that are heterogeneously present, with variability depending on the type of material (Fig 5 and 6, S1-S4 Data). In general terms, natural processes related to karst activity, such as concretions, manganese deposits, and sedimentary infillings, are abundant, as are those associated with the marine-coastal ecosystem, including abrasion, lithophagous perforations, and encrustations, which implies that these shells were mostly gathered from thanatocoenoses. However, the most characteristic taphonomic alterations are of anthropogenic origin. The abundant presence of ochre may be attributable to sedimentary layers rich in this material (e.g., Galería Levels IV and III), although it could also result from suspension using an ochre-treated hide cord or from contact with processed or ochre-painted hides, whose antiseptic properties inhibit putrefaction and desiccation [95,96]. A tendency can be observed –particularly during the Middle Magdalenian– for ochre to accumulate within the grooves of the shell spirals, as well as on surface areas polished through use, where it has been preserved within the micro-pores of the shell surfaces (S10 Fig), which does not preclude the possibility that some of these elements were deliberately painted, or in intense contact with painted skin or clothes. Cut marks on the root of some teeth suggest their extraction from animal carcasses by cutting through the gingival tissue [76]. Thermal alteration affects only a single *Littorina obtusata* specimen and three atrophic red deer canines, most probably resulting from incidental hearth exposure rather than intentional aesthetic burning, as observed in other Paleolithic assemblages [97–99].

The most common anthropogenic modification is perforation, indicating a predominance of finished ornamental elements over those considered raw material. This proportion varies between shell elements and those made of teeth or bone: in the former, anthropogenic perforations account for 86.67%, while in the latter, they represent 56.67%.

Finally, fracturing may result from factors related to sedimentary processes, trampling, technical errors during manufacturing, and other similar causes. In the studied assemblage, 39.48% of the elements exhibit some type of fracture, more frequently in shell elements, providing insight into the overall good state of preservation of the collection.

### Use wear-analysis

Among the 208 specimens deliberately conditioned as ornaments, 200 bear one perforation, five have two perforations, and one single specimen has three perforations. Three specimens of *L. obtusata/fabalis* perforated by lithophagous organisms in areas that could be used for ornamental purposes are also included among the single-perforated elements. The remaining two pieces correspond to tubular fragments, which therefore lack proper perforations. On the other hand, 59 items do not show anthropic modifications, although the absence of perforations in 19 of them cannot be confirmed due to the degree of fragmentation. Of the 41 pieces in which the absence of perforations is verifiable, 36 correspond to deer (*C. elaphus*) canines, and five to *L. obtusata/fabalis* and *Tritia* shells. Finally, the remaining three pieces correspond to ornaments whose manufacturing process was left incomplete (S2 Data).

### Production of personal ornaments

**Fig 7:**
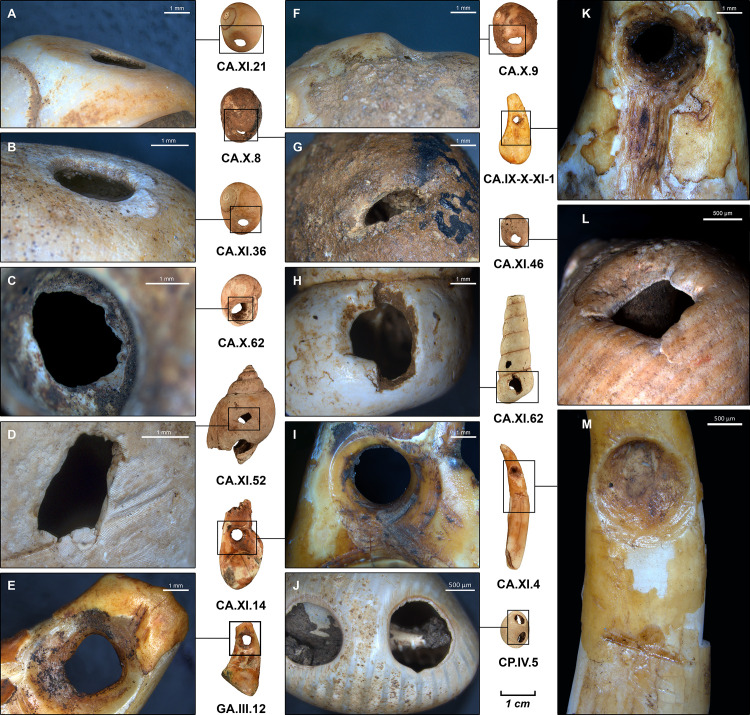
Manufacture techniques present in the assemblage. A,F. Abrasion, B,J. Abrasion and rotation, **C.** External pressure, **D.** Incision and pressure/percussion, E,K. Incision and rotation, **G.** Sawing, H,L. Internal pressure/percussion, **I.** Rotation, **M.** *Capra pyrenaica* tooth bearing two unfinished perforations, made through incision and rotation, which provides evidence of ornament manufacture within the cave.

We have determined the use of 8 different preparation and/or perforation techniques (Figs 3 and 7), which vary depending on the raw material and taxon used. In shell beads, abrasion is predominantly used, often combined with rotation or pressure/percussion. This technique uses flat abrasive surfaces, although some beads show traces of abrasion employing a convex surface as a matrix, perforating the shell by creating an elongated U-shaped channel (Fig 7F). Evidence for the use of this technique includes flattened surfaces, thinning around the perforation, and parallel micro-striations aligned with the direction of the abrasive motion (S12 Fig in S2 File). Rotation in shell ornaments is rarer, but for certain taxa, such as *Trivia* sp, it is used almost exclusively. This technique implies a rotative motion with a lithic implement and is extensively employed on all tooth and bone elements across all the chronological phases represented at Llonín, either as a standalone method or in combination with preparatory incision, abrasion, or both. It produces perforations with a generally circular outline, conical or biconical cross-sections, and concentric circular striations along the edges of the perforation (S12 Fig in S2 File). Other techniques, used less frequently and with particular variability in Solutrean levels, respond to specific morphologies of the shells or specific cases. An example of this is the use of flexion in the manufacture of *Antalis* sp beads, sawing in a specimen of *L. obtusata/fabalis*, or preparation by incision in one specimen of *Tritia mutabilis*. The salmonid vertebrae documented in the Solutrean level of the Galería sector warrant special attention. These elements were transversely sectioned by sawing and subsequently perforated by rotation. An alternative method for separating the articular surfaces of vertebrae, as demonstrated experimentally (S12 Fig in S2 File), involves percussion. However, this technique significantly alters the morphology of the resulting beads, producing concave profiles and fragile, irregular forms, a pattern not observed in the archaeological specimens.

Lastly, the pressure technique is traceologically indistinguishable from indirect percussion, as it leaves similar traces on the perforations (S12 Fig in S2 File); however, it is possible to determine whether the perforation was made from the outer or inner face of the shell, as a characteristic bevel is formed on the surface opposite to the one receiving the pressure or percussion [28] (S12 Fig in S2 File). Various materials have been proposed for executing these techniques, ranging from antler or bone to wood or crab claws [5,28]. Perforation from the inside has been used in the vast majority of shells, except for some specimens of *L. obtusata/fabalis* and *T. tricarinata*.

A greater homogeneity is perceived among tooth and bone beads from terrestrial fauna, due to the natural hardness and density they present. Bipolar rotation is used unanimously, often accompanied by preparation through incision (Fig 3). In certain tooth and bone elements, other techniques are occasionally applied, such as root thinning by abrasion. In the case of *Equus* hyoids and the bone disk, trimming was used to give the piece the desired shape before perforation by rotation. Although the combination of techniques is common, the application of a single technique predominates in most finished ornaments.

The Llonín collection includes 3 unfinished ornaments, which were discarded or lost during the manufacturing process, consisting of two deer canines and one caprine incisor. In the latter, an initial transversal line was traced by incision, aiming to delimit the perforation area or create a stable support point for applying a rotational movement. This first attempt was abandoned, and the same method was applied to a more proximal part of the root, which ultimately led to the piece being left unfinished (Fig 7M). The two deer canines show two distinct manufacturing methods: in one of them, rotation was started directly without prior preparation and was abandoned in an early stage of the process; while in the other, parallel incisions were made that possibly delineated and flanked the area where rotation would begin.

The preferred perforation areas in shells, bones, and teeth are heavily influenced by both the morphology of the taxon and the type of suspension intended for each bead [27] (S7 Fig in S1 File). In spiral gastropods, perforation near the edge of the labrum (E1) predominates (81.13%), although perforations in E2, E3, and E4 are also documented, especially in Solutrean levels. The combination of perforations in E1 and E4 is exclusive to the genus *Trivia*. Regarding non-spiral gastropods, two specimens were perforated in A3 and one in A2; while the bivalve specimens were perforated in Z1, except *C. islandica*, which was perforated in Z2. Tooth ornaments bear perforations exclusively in the root. The remaining elements were perforated in areas according to their morphology, as seen in circular pieces perforated at the center, such as fossils or fish vertebrae; and in flattened quadrangular elements, such as hyoids or metapodial fragments, perforated at one of their longitudinal ends. For *Antalis* sp. specimens, no perforation zone has been defined, as their tubular structure serves this function (S1 Data).

The morphology of the perforations is conditioned by factors such as the shape of the support, the technique used, the purpose, and the taphonomic processes involved. Certain techniques generate repetitive and standardized shapes, while others produce highly varied morphologies. Rotation is associated with regular circular shapes, while abrasion is linked to oval or elongated shapes. Pressure and indirect percussion mainly generate irregular shapes. Sub-regular and quadrangular morphologies are less commonly associated with all the documented techniques, drawing from the range of morphological variability generated by abrasion, pressure, and percussion techniques (S12 Fig in S1 File). The variability range of pressure and percussion is also reflected in the morphometric values of the perforations (S1-S4 Data), as these techniques allow for less control of the drilling process, resulting in more diverse holes in terms of morphology and morphometry. Abrasion and rotation generate more homogeneous perforations.

### Use and suspension systems of personal ornaments

**Fig 8:**
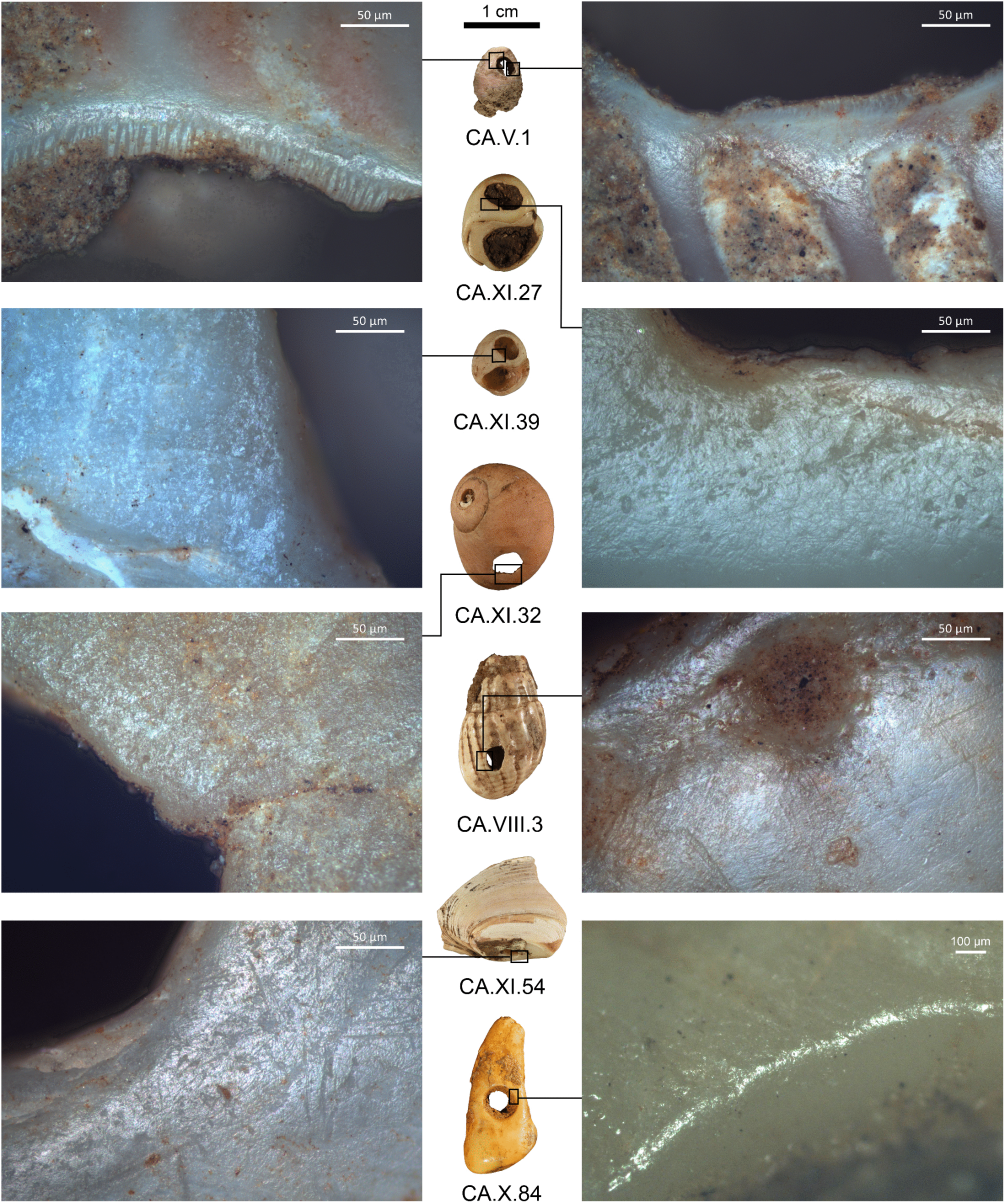
Micro-polish and striations on perforation edges. Prolonged use and friction against the thread and the wearer generates use-wear visible at microscopic level (50 to 200x magnification).

**Fig 9:**
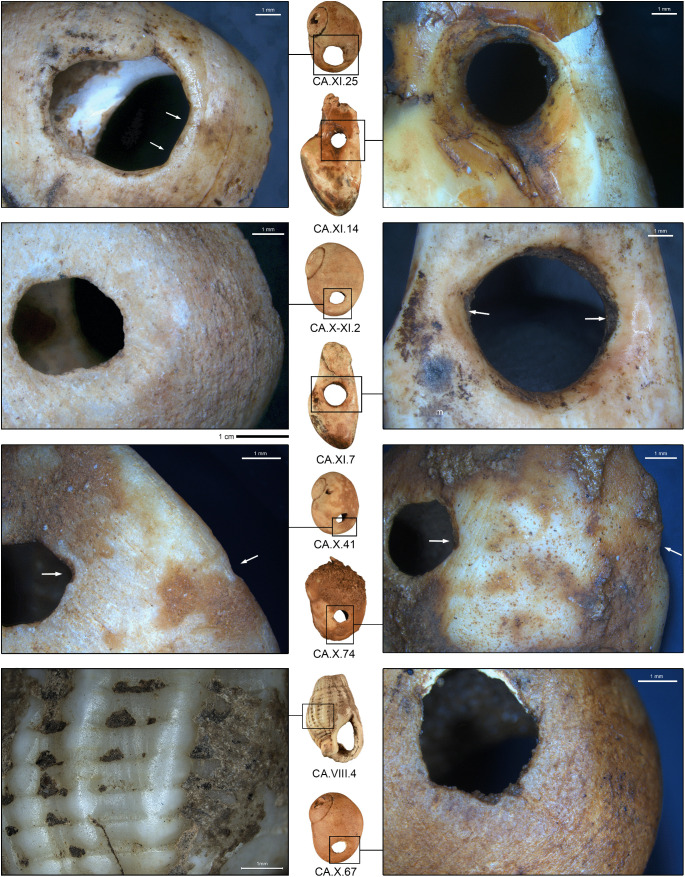
Rounding, enlargements, notches, and deformation of the perforations. At the macroscopic level (10–50 × magnification), prolonged use alters the shape of the edges, fades the manufacturing traces, and enlarges the perforations depending on the stringing method applied.

Continued suspension of personal ornaments generates friction between the suspended element and the suspensor, the ornament and the wearer, or between ornamental pieces, which favors the appearance of use-wear in the form of notches, enlargements, rounding, polishing, striations and deformations in the perforations [27] (Figs 8 and 9), visible at the microscopic level, that help locate the contact areas and infer the suspension methods used [29,33]. The attenuation of technological marks around the perforation edges (rotational traces, scraping, bevels…) is also indicative of use [30,37] (S13 Fig in S2 File).

Taphonomic processes present in the assemblage have limited the analysis of use-wear to 170 of the 208 finished beads (S1-S4 Data). The main taphonomic limitations affecting use-wear analysis relate to the fragmented condition of several specimens, such that the preserved remains were not sufficiently diagnostic. In addition, the presence of abundant concretions or sediment infilling within perforations and on the surfaces of some ornaments, as well as advanced biodegradation or dissolution, has in some cases hindered the analysis of use-wear. The study determined that 157 (92.35%) of the analyzed ornaments were used, although in 16 cases, said use is doubtful. No use-wear was found on the remaining 13 elements. In addition, no proportional differences are observed by material, with 91.3% of the shell elements and 96.77% of the bone and tooth ornaments showing use-wear traces. Although we have not conducted a quantitative study of the degree of use, the variability and development of wear traces are more noticeable in upper Solutrean levels than in later periods.

**Fig 10:**
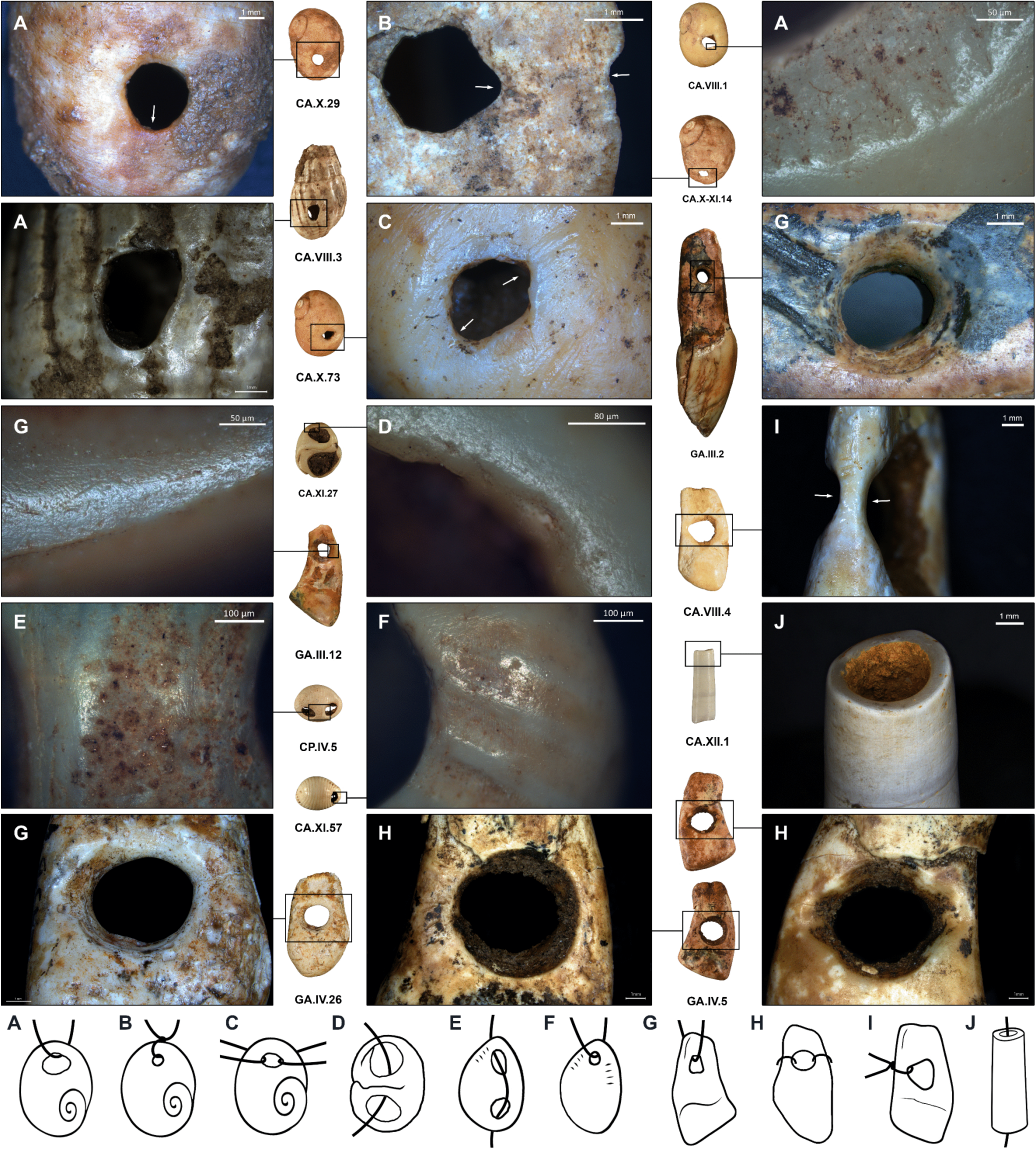
Evidence of use and suspension hypotheses. Notches, rounding, deformations, and microscopic polish observed in the assemblage are interpreted as traces resulting from use and suspension as personal ornaments. Correlated with these traces, proposed hypotheses of suspension are presented according to the different documented wear patterns.

Regarding the suspension modes and use of personal ornaments, several studies [27,33,36,87,88,90,100] have aimed to infer their arrangement based on the analysis of perforation zones and wear traces caused by friction with the suspending element or the body. These premises allow for suggesting various possible arrangements for the studied specimens (Fig 10). Stringing methods are more influenced by the perforation zones selected for each taxon than by the technique employed. However, in some cases, the morphology resulting from this technique determines the subsequent suspension mode of the shell. In the case of tubular beads, such as *Antalis* sp. fragments, suspension would have been achieved by threading a cord longitudinally through the piece; moreover, these shells could subsequently be arranged in a wide variety of configurations [35,100]. Some shells, such as *Trivia* specimens often display two perforations, which enables specific stringing methods, by threading the cord through both perforations, in a parallel display.

Gastropods, particularly *L. obtusata/fabalis*, display the greatest variability in suspension modes documented, although evidence of free suspension (Fig 10A) predominates over knotted or lateral suspension (Fig 10B–C). Tooth and bone elements likewise predominantly exhibit free suspension, although certain specimens show clear evidence of having been sewn, as indicated by marked differences in use-wear between their two faces (Fig 10H). Finally, particular attention should be drawn to an atrophic canine from the Upper Magdalenian (CA.VIII.4) (Fig 10I), as its use-wear marks are present exclusively and intensely on one side of the tooth’s root. This position does not physically align with a vertical suspension and the opposite end shows much fewer signs of wear, suggesting that this specimen was likely strung and attached solely through one side, and subjected to significant tension, which leads us to hypothesize that it may have functioned as a button. Additionally, the level of wear indicates that it was heavily used before being decommissioned.

### Decorated elements

**Fig 11:**
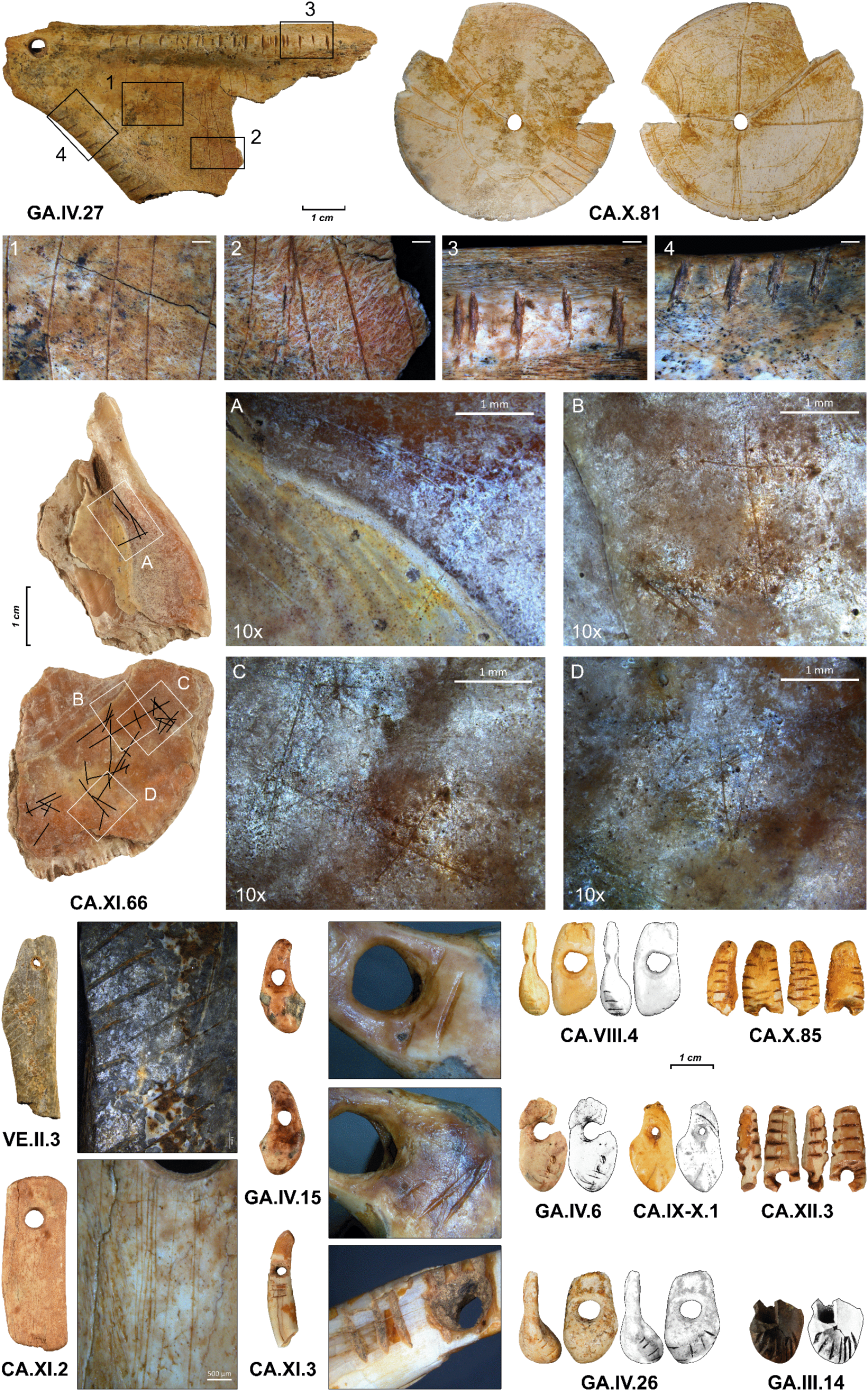
Decorated specimens. Red deer canines (CA.VIII.4, GA.IV.26, GA.IV.6, GA.III.14, CA.IX-X.1, GA.IV.15), wolf canine (CA.X.85), bovine incisor (CA.XII.3), horse hyoid bones (VE.II.3, CA.XI.2), caprine incisor (CA.XI.3), red deer scapula (GA.IV.27), perforated bone disk (CA.X.81), *Chlamys islandica* shell (CA.XI.66).

The decoration of personal ornaments made from teeth and bone is a relatively common practice in European Paleolithic since the Aurignacian, becoming recurrent during and after the Solutrean period [80]. Nevertheless, the proportion of decorated elements compared to undecorated pieces remains very low. The Llonín assemblage comprises 14 decorated specimens (Fig 11), all of which feature sequences of parallel, concentric, or longitudinal incisions of a non-figurative geometric nature. In certain cases (CA.XI.3, GA.IV.15, CA.IX-X.1), the decorative nature of the incisions is uncertain, as they coincide or are related to similar functional grooves intended for perforation preparation or delimitation. In other instances (CA.XI.2), longitudinal incisions made before perforation could be attributed to cut marks or the cleaning process of the hyoid bone. The remaining specimens show no doubts regarding their intentionally decorative nature. The atrophic red deer canines (GA.IV.6, GA.IV.26, CA.VIII.4, and GA.III.14) exhibit 4, 10, 4, and 7 incisions, respectively, all of them short and fine, except for the last specimen, which presents wider and deeper concentric incisions. The decoration is located on the enamel, particularly in the occlusal wear area.

The wolf canine (CA.X.85) and the *Bos/Bison* sp incisor (CA.XII.3) display very similar decorations despite their different chrono-cultural attributions, consisting of numerous (12 and 23, respectively) deep, wide, parallel grooves made through bidirectional sawing along the entire root surface. The assemblage also includes a bone disk previously described by Fortea et al. [101] (CA.X.81), a fragment of an *Equus* hyoid bone decorated with 19 fine oblique parallel incisions (VE.II.3), and a perforated scapula decorated on its anterior face with 20 parallel incisions on the cranial edge, nine on the caudal edge, and numerous long, fine incisions across the entire anterior scapular surface (GA.IV.27). Incisions made on the cranial and caudal edges employed bidirectional sawing. In contrast, those made on the scapular surface were produced through fine single incisions.

Decoration on shell ornaments is extremely rare throughout the Paleolithic. However, the inner surface of the *C. islandica* specimen from Llonín shows possible engraved incisions (Fig 11). These markings are exceptionally thin and barely perceptible, raising doubts about a possible taphonomic origin. Nevertheless, the non-random arrangement of the lines, creating parallel patterns and geometrically intersecting shapes, suggests an anthropogenic origin. Similarly, this pseudo-reticular layout and the presence of incisions across the entire inner surface rule out the possibility that they are cut marks related to the mollusk’s consumption.

## Discussion

### Raw material procurement

The acquisition process of raw materials provides vital information about the context, production strategies and mobility of human groups. The malacological assemblage of Llonín indicates a primary ornamental intent, as more than 95% of the ornamental shells come from taxa with no nutritional value (Table 1), except for *Patella*, *Veneridae*, *Glycymeris*, and *L. littorea* specimens. The study of mollusks with nutritional value is currently ongoing; however, procurement primarily for ornamental purposes is inferred again, from the relatively low number of edible mollusks in proportion to the specimens modified into pendants. For instance, the total number of remains from the Solutrean levels across all excavation sectors—mainly fragments of *Patella* and *Littorina littorea*—does not exceed 60 pieces, whereas 64 shell ornaments or raw material remains intended for ornamental use have been documented.

Regarding their geographical origin, most of the studied taxa are commonly found in the Cantabrian Sea, although many of them, such as *Antalis*, *Tritia*, or *Turritellinella* sp., are also present in Mediterranean waters. An exception to this pattern is represented by the two documented specimens of *Tritia mutabilis*, one assigned to the Upper Solutrean (CA.XI.52) and the other lacking a clear chronological attribution. This species does not currently inhabit the Cantabrian coast; therefore, it is likely that during the Paleolithic its ecological niche was restricted to the Mediterranean [19]. Likewise, cold-water specimens such as *Littorina* sp. or *N. lapillus* may have been present in the Mediterranean during the LGM [102]. Llonín cave is located less than 13 km in a straight line from the sea during the LGM; however, the orography favors a detour around the Sierra del Cuera mountains, following the course of the Cares River to reach the coastal platform. This means the actual distance may have been tripled, although there are shorter routes that require greater energy expenditure, either through mountain passes or by traveling upstream through other contemporary sites like La Covaciella, El Bosque or Coimbre (S1 Fig in S1 File). The collection of raw materials may, therefore, have been carried out by the inhabitants of Llonín on multi-day expeditions which included the occasional gathering of edible shellfish, or brought to the site by groups from the coastal strip as items of exchange or gift. Regarding the Mediterranean taxa, they were incorporated through long range contact networks, which will be further discussed below.

Criteria for shell selection were influenced by technical factors (perforation feasibility and availability in the ecosystem), and symbolic/aesthetic factors (taxon, morphology, size, shine, color, etc.). The predominance of *L. obtusata/fabalis* is consistent throughout the entire sequence. This preference may be linked to its abundance, aesthetic appeal, the range of techniques and perforation zones it offers, and especially its wide range of colors, which would allow for the creation of compositions with a chromatic component. The presence of lithophagous attacks and marine abrasion in several shells (S1 Data) suggests that most of them were collected from thanatocoenoses. Additionally, certain taxa inhabit deeper subtidal zones and the continental shelf (S2 Text in S1 File), such as *Antalis* sp., *Chlamys islandica*, *Glycymeris* sp. or *Trivia* sp., also suggesting that they were collected along the shoreline. The biometric study suggests an indiscriminate collection of *Trivia* and *Littorina* shells along the sequence, except during the Middle Magdalenian, when a deliberately biased search by size among *L. obtusata/fabalis* shells is reported (S1 Data and Fig 4). This diachronic difference is also observed in the selection of taxa. During the Solutrean, ornaments were made from a wide taxonomic range of mollusks, while during the Magdalenian, *L. obtusata/fabalis* is almost exclusively documented (Table 1).

Acquisition of bone and teeth also comes from the local-regional area, both from terrestrial and riverine-marine fauna, coincident with the typically Pleistocene fauna of the Cantabrian region [103–105], suggesting they were obtained through hunting activities. The presence of cut marks on several teeth surfaces (S2 Data) supports this interpretation [76]. With regard to the archaeozoological evidence from Llonín, Gravettian occupations of the cave show a clear predominance of caprines (*Capra pyrenaica* and *Rupicapra pyrenaica*), consistent with the rugged topography of this pre-Alpine environment [106]. This pattern shifts in the Upper Solutrean levels, where an intensive exploitation of red deer and ibex is documented, with chamois playing a secondary role [107]. During the Badegoulian occupations, *Rupicapra pyrenaica* once again constitutes the predominant taxon, surpassing both red deer and ibex, while horse and aurochs are only occasionally recorded [64]. We consider that the imbalance between the most hunted species and the preferred ornamental raw materials responds to a human decision rather than a matter of availability. Deer vestigial canines were likely imbued with a special symbolic, standardized and globalized status, justified not only by the strong recurrence of these teeth transformed into ornaments but also by the existence of numerous imitations in other materials such as bone, antler, ivory, or mineral; in several European contexts such as Tito Bustillo, Gatzarria, Gourdan, Pech de la Boissière, Rigney or Continenza [80,108–110]. No clear preference is reported in the selection of canine teeth regarding laterality, sex, or age at any point of the sequence (Fig 4 and S2 Data). The most notable aspect is the high MNI represented in the sample, which amounts to 71 (29 Solutrean, 14 Badegoulian, 9 M. Magdalenian, 6 U. Magdalenian, 1 Azilian, 12 undetermined), and, coupled with the absence of paired specimens, leads us to consider the acquisition of canine teeth through means other than exclusively hunting or carcass scavenging, such as the deliberate exchange of canines between individuals of the same group or different groups [76].

**Fig 12:**
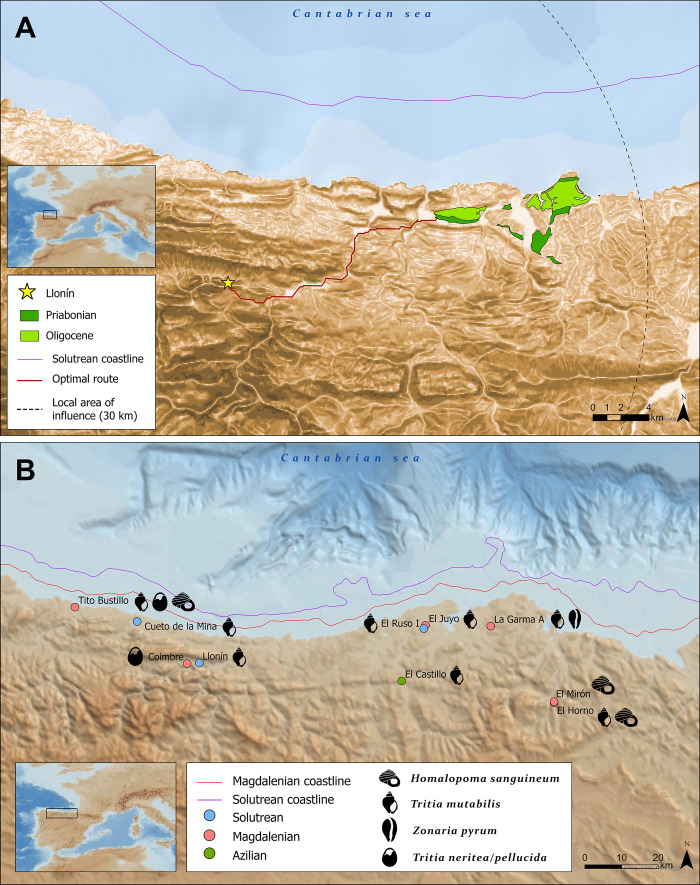
*Rotularia* sp. fossil geological sources and distribution of Mediterranean shell occurrences in the Cantabrian region. **(A)** Location of marine Priabonian and Oligocene outcrops in relation to Llonín cave through a least-cost-path analysis. The dotted line represents how these outcrops fall within what Gamble [114] and Fuentes et al. [115] defined as the local area of influence. Geological layers provided by the Instituto Geológico y Minero de España [116]. **(B)** Distribution of Solutrean, Magdalenian and Azilian Cantabrian sites where shells of Mediterranean origin have been documented. The Solutrean and Magdalenian coastlines employed on both maps derive from the Paleocoastlines GIS dataset developed by Zickel et al. [117] and from the sea-level reconstructions proposed by Bilbao-Lasa et al. [59].

Lastly, the fossil tube worm specimen (Fig 2) is present in marine geological deposits dating from the Kimmeridgian to the Oligocene [75,111]. There are Eocene and Oligocene marine outcrops in the immediate surroundings of the Llonín cave [112–113], with the closest being less than 20 km away (Fig 12). While we consider the nearby local origin as the most plausible option, the exotic nature of the piece cannot be ruled out. Similar specimens have been found in other Cantabrian contexts such as La Garma A (Cantabria) or Bolinkoba (Basque Country), although in magdalenian rather than solutrean layers [69]. The procurement of fossils through long-distance contacts is evidenced by the presence of *Vitta picta* specimens originating from southwestern France in the Gravettian levels of La Garma A and Aitzbitarte III [118] and is particularly well attested in French Upper Paleolithic contexts such as Abri Lachaud, Abri Fritsch, or Mont Saint Aubin [35].

### Manufacture techniques and suspension modes

The techniques chosen for ornament manufacturing respond to the physical properties of the raw materials used. Thick materials such as teeth, bone, and fossils employ rotation exclusively throughout the sequence, preceded in around 70% of the cases by an incision-based preparation (Figs 3 and 7). In the case of mollusks, this technical consistency disappears. Shell beads are perforated using a wide range of techniques, although abrasion is the most commonly used, both for surface preparation prior to perforation and for the execution of the perforation itself (Fig 3). Despite being more labor-intensive, this method reduces the risk of technical failure and allows for the production of beads with highly standardized perforations. The use of surface preparation techniques is significantly less frequent than that documented for tooth and bone elements and is largely limited to abrasion (Fig 3). A difference in technical and morphological diversity is observed between the different phases of the sequence. During the Upper Solutrean, the full spectrum of documented techniques, taxa, and perforation zones is used; however, in the Middle Magdalenian, abrasion near the edge of the labrum (E1) predominates in over 85% of shell ornaments, frequently complemented by rotation. This suggests a standardization and homogenization of manufacturing processes in this phase of the sequence (Fig 3 and S1-S2 Data).

Detailed analysis of the perforation zones and associated use-wear traces (Figs 8 and 9), resulting from friction with suspension elements or direct contact with the body, has enabled the formulation of hypotheses regarding the suspension systems and functional use of the personal ornaments under study (Fig 10). The proposed reconstructions suggest multiple possible arrangements for the analyzed specimens, with morphological and spatial choices in perforation placement often exerting greater influence than the specific manufacturing techniques employed. Ultimately, the appearance of the ornaments formed by these beads or the items to which they were sewn can only be hypothesized, often based on ethnographic comparisons. In this regard, White [119] suggests that these elements were used in necklaces, bracelets, anklets, and earrings, as well as sewn onto clothing or attached to various objects such as bags, baskets, blankets, and weapons.

### Social functions

The social functions and symbolic sphere surrounding personal ornaments likely varied diachronically and geographically, depending on the specific circumstances of each region and period [80]. Beads can be considered irreducible elements of potentially complex communication systems, susceptible to being recombined, relocated, and reformulated in terms of symbolic meaning [1]. Studies based on ethnography have proposed a broad range of functions for personal ornaments [39]. The most commonly associated with Paleolithic groups are aesthetics or self-affirmation of individual status, identification with an ethnic group or social category, use as trade goods, or involvement in ritual acts.

In Llonín cave, the context of bead production and its temporal variability are critical for interpreting the potential social functions of these ornaments. During the Upper Solutrean, partially perforated mammal teeth and 16 unperforated deer canines are documented alongside finished beads, providing direct evidence for the in-situ manufacture of teeth ornaments. With regard to shells, some specimens exhibit no use-wear and display fracture patterns consistent with technical errors incurred during perforation. Likewise, three non-perforated *Littorina obtusata/fabalis* specimens were recorded; these may have been discarded due to their small size or peristome breakage, factors that, however, did not prevent basal perforation in other similar specimens. The Solutrean level (IV) of Galería also yields the highest ornament density at both the diachronic and synchronic scales within the entire site (S3 Table in S1 File). Collectively, these results demonstrate that ornament production occurred within the Llonín site itself, on a limited, likely group-scale level, and focused on a diverse range of materials generally available in the local environment. Furthermore, the heterogeneity in terms of techniques, choices, and perforation zones visible in this period points to the existence of different crafters with distinct goals and preferences. This suggests a more individual and personal function of the ornaments, although we cannot be certain whether they were worn as hunting trophies, body adornments, talismans, or other purposes. In any case, we can identify them as markers of individual identity. The Badegoulian record, despite its small volume, lends itself to a similar interpretation, judging by the variety of typologies and taxa, with a notable presence of a decorated specimen (GA.III.14) (Fig 11) and three incisors of *Bos/Bison* sp., which, based on their location, technological, use-wear and taphonomic characteristics (S3 Data), appear to belong to the same pendant. The scarcity of shell beads contrasts with the general dynamics of the context. It could be attributed to reduced mobility or interaction of the Llonín inhabitants with the coastal area or the groups living there, within the context of worsening climatic conditions during the Last Glacial Maximum. However, this interpretation is contradicted by the variety and quantity of mollusks found in the cave during the Upper Solutrean. In general terms, the lower density of ornaments during this phase may be related to the specialized nature of the Badegoulian occupation, closely associated with fire-related activities (e.g., smoking, drying, hide processing), in contrast to the preceding phase, in which a broader range of symbolic, economic, and technical activities is documented, in accordance to its interpretation as a residential site [61,64] (S1 Text in S1 File).

On the other hand, the Middle Magdalenian assemblage presents different characteristics from the Solutrean. The malacofaunal sample, already technically, biometrically, and typologically less varied, lacks unperforated specimens or verifiable technical failures (S1 Data); suggesting that the ornaments were brought to the site already manufactured. The fact that most of the fragmented specimens show use wear on the preserved edge of the perforation rules out the possibility that they were fractured during the manufacturing process. In this sense, we propose that the arrival of the shell beads was related to an event of aggregation or exchange that took place in the cave itself. These are interpreted as contexts in which dispersed hunter-gatherer groups aggregated at a single location for varying durations in response to economic, social, or ritual factors [120]. The interpretation of Llonín as a recurrent aggregation site during the Middle Magdalenian has already been suggested by other authors [68], based on the rich thematic diversity of the bone industry and portable art, as well as the rich parietal graphic assemblage [66].

The Middle Magdalenian assemblage suggests a collective social nature and function, given the level of homogeneity and standardization presented (Fig 3, S1-S3 Data and S8 Fig in S1 File). During aggregation contexts, this could translate into their use as group identity markers, or as exchange goods. In an inland site like Llonín, shell ornaments may have been exchanged for bone and tooth items obtained from local fauna and manufactured at the cave itself. This interpretation coincides, at least, with the scarce record of bone and tooth ornaments from Middle Magdalenian layers; among which the perforated bone disk [101] stands out, considered by some authors [121] as a standardized element used to distinguish individuals or groups during social interactions. Use wear traces on Middle Magdalenian specimens of *L. obtusata/fabalis* are less developed than those from other periods, suggesting they were likely used for a shorter period of time before being discarded or worn out, possibly as part of the process. The fact that the elements are displaced from their original position due to the sedimentary dynamics of the dejection cone prevents further interpretations based on the location of the pieces.

During the Upper Magdalenian, a techno-economic and symbolic regionalization is observed, with less thematic diversification in bone industry and parietal art [66,68]; contrasting with a greater taxonomic and typological variety in ornaments than that observed in the previous phase, despite only having 14 specimens (Fig 3). These characteristics refer to what has been proposed for the Solutrean period, with greater heterogeneity of techniques, models, and artisans; associated with a more individualistic character in terms of the social use of these ornaments.

### Cultural networks and mobility

Personal ornaments provide a valuable *proxy* for reconstructing intergroup networks and patterns of hunter-gatherer mobility [17–23,80,122,123]. In this regard, Llonín Cave is strategically located within an east–west corridor that links the western Cantabrian region with two major routes: one leading north of the Pyrenees and another connecting to the Mediterranean coast through the Ebro valley [17,19,80,124] (Fig 1). Within these networks, the circulation of raw materials is well attested by the abundant presence of lithic elements originating from the eastern Cantabrian area and the northern Pyrenees [19,64,118], as well as the formal parallels observed in mobiliary and parietal graphic representations [125–130].

Regarding the lithic industries, the entire sequence shows a clear predominance of immediately local raw materials. In fact, within the Badegoulian assemblages from Gallery Level III, more than 90% of the lithic elements originate from sources located within 5 km of the site [64]. In addition, raw materials from local and semi-local environments are also documented, including quartzites, lutites, radiolarites, and flint, within a range extending approximately 40–85 km to the east (Monte Picota and Virgen del Mar-type flints) [131–132], and 60–100 km to the west (Piloña and Piedramuelle-type flints) [64,133,134]. This pattern, characterized by the predominance of local raw materials complemented by exogenous elements, continues throughout the Magdalenian, during which the transport of Piloña flint cores to Llonín Cave has been documented [133]. The existence of east–west procurement and exchange routes is well documented by the presence of northwestern Pyrenean (e.g., Chalosse, Bidache flints) or eastern Cantabrian (e.g., Treviño, Urbasa, Flysch flints) raw materials in several Upper Paleolithic contexts from the central-western Cantabrian region, including sites such as El Juyo, Altamira, El Cierro, Coimbre, Cova Rosa, Tito Bustillo, Llonín, La Viña, and Las Caldas [118,135,136]. Conversely, raw materials from the western Cantabrian region (e.g., Piloña flint) have also been identified in central sites such as Cualventi, El Linar, and Las Aguas [133,137].

The study of osseous artifacts made from whale bone further supports the existence of long-distance exchanges along this Cantabrian–Pyrenean axis during the Magdalenian, involving key reception sites and major nodes such as central Asturias (e.g., Las Caldas and La Viña), the Ariège region (e.g., Mas d’Azil and La Vache) and, mainly, the Franco–Spanish Basque Country (e.g., Ermittia and Isturitz) [138,139]. Llonín itself lies in a natural passage through the Cares River valley, linking western Asturias to the central-eastern section of the Cantabrian corridor, an area rich in Upper Paleolithic contexts (e.g., La Covaciella, Coimbre, El Bosque, Arangas, etc.) [64,140–143] (S1 Fig in S1 File). This setting made it a likely aggregation and reception point [68], consistent with the large number of beads documented at the site.

Focusing on the personal ornaments, the Llonín assemblage fits within the general patterns of the Late Upper Paleolithic in the Cantabrian region, characterized by a predominance of shell elements. While shell and dental ornaments—mainly atrophic red deer canines—occurred in roughly similar proportions during the Last Glacial Maximum, shell use became clearly preeminent throughout the Magdalenian. Other raw materials such as bone, antler, fossils, or minerals occur only sporadically [80,144]. The region exhibits remarkable malacological diversity through time, shaped by local raw material availability and shifting cultural preferences. Yet, during the Magdalenian *L. obtusata/fabalis* emerged as the dominant taxon throughout the region. Similar trends appear at other major Cantabrian sites, both Solutrean (e.g., La Riera, Cueto de la Mina, El Mirón, Bolinkoba) and Magdalenian (e.g., Tito Bustillo, El Juyo, El Mirón, El Pendo, Coimbre, Altamira, Cualventi, La Garma A, Rascaño, Santa Catalina, Urtiaga) [80,86,145–147]. This broad consistency points to a shared cultural background and continuity in ornamental preferences across the Cantabrian corridor, with local nuances reflecting environmental and cultural constraints [144]. This coherence was further strengthened by east–west mobility and by exchange networks through which lithic and osseous raw materials, finished items, personal ornaments, information, and symbolic conventions circulated [19,64,66,118,126–129,139].

In relation to the Cantabrian–Mediterranean axis, the presence of strictly Mediterranean species such as *Tritia mutabilis* in Llonín cave confirms the existence of long-distance networks and contacts from the Solutrean onwards, linking the Iberian Mediterranean coast with the Cantabrian region, probably through the natural corridor of the Ebro Valley [80]. The earliest evidence of Mediterranean mollusks in the Cantabrian area corresponds to a specimen of *Luria lurida* from the Gravettian levels of Bolinkoba; however, it is not until the end of the Solutrean that such evidence becomes more recurrent. *Tritia mutabilis* appears contemporaneously with Llonín in the Upper Solutrean levels of Cueto de la Mina [148] and El Ruso I [149] (Fig 12). During the Magdalenian, although absent from the Llonín assemblage, evidence of Mediterranean-cantabrian exchange networks increases, with specimens of taxa such as *Tritia neritea/pellucida*, *Homalopoma sanguineum*, *Zonaria pyrum*, in addition to *Tritia mutabilis*, documented in contexts such as El Horno, El Juyo, El Castillo, Tito Bustillo, El Mirón, or La Garma A [17,19,80,150]. Besides, the presence of several *Tritia neritea/pellucida* specimens in Level 1 of the nearby site of Coimbre B (Upper Magdalenian) [19] (Fig 12), located less than five kilometers upstream, indicates that such connections did occur within Llonín’s immediate surroundings during broadly contemporaneous phases.

Taken together, the available evidence suggests the existence of two complementary forms of mobility around Llonín, a pattern also documented at regional sites such as Las Caldas [124]. One operated at a local scale associated with logistical movements for acquiring subsistence resources and raw materials [42–51], from which certain ornamental substrates—teeth, bone, shells, and possibly fossils—were procured. The second mode encompassed broader, non-utilitarian mobility [122] along east–west and coastal–inland axes, positioning Llonín as a significant node within the wider network of intergroup communication linking the western Cantabrian region and both the northern Pyrenees and the Iberian Mediterranean coast. Through these connections, Llonín’s foragers engaged in, and benefited from, the circulation of raw materials, finished ornaments, and symbolic traditions.

## Conclusions

The results obtained provide a basis for discussing the identity and mobility of hunter-gatherer populations and the ornaments associated with them. During the Upper Solutrean, small-scale manufacture of personal ornaments is documented, in consistence with a residential occupation pattern, likely serving a social role as individual identity markers within a context of frequent population movements across the surrounding region. This interpretation is supported by the pronounced heterogeneity in locally sourced taxa, manufacturing techniques, use-wear intensity, suspension methods, and ornamental typologies, as well as the complete representation of the ornament production *chaîne opératoire*. Similar, though more limited, processes are documented in the Badegoulian and Late Magdalenian layers. In contrast, during the Middle Magdalenian, the cave may have served as an aggregation site for hunter-gatherers, acting as a node in the long-range networks extending along the west–east axis. Llonín cave became a repository for finished ornaments produced elsewhere along the coastal area. These objects may have been linked the collective identity of a group or to related individuals who gathered at the site during events of social interaction. This is supported by the marked homogeneity combined with low techno-typological and taxonomic diversity of the assemblage. Llonín Cave thus emerges as a key context for understanding diachronic social interaction and mobility dynamics among Iberian Upper Paleolithic hunter-gatherers. It serves as a reference point for approaching these questions from a functional and social perspective, moving beyond purely technological analyses of ornament assemblages.

## Supporting information

S1 Data Detailed analytical dataset of shell personal ornaments and raw materials. (ZIP)

S2 Data Detailed analytical dataset of teeth personal ornaments and raw materials. (ZIP)

S3 Data Detailed analytical dataset of bone personal ornaments. (ZIP)

S4 Data Detailed analytical dataset of fossil personal ornaments. (ZIP)

S5 Data Biometric data of modern *Littorina obtusata/fabalis* and *Trivia* sp. shells. (ZIP)

S1 File Site description (S1 Text). Information regarding the ecology of the mollusk taxa identified in Llonín cave **(S2 Text)**. Radiocarbon dates. Absolute dates for Levels III (Badegoulian), IV (Upper Solutrean) and V (Gravettian) from Galería, and Level VIa (Gravettian) from Cono Posterior. Original dates recalibrated using IntCal20 **(S1 Table)**. List of references consulted for the creation of Fig 1. Numbers refer to the bibliography cited above **(S2 Table)**. Summary of sectors and levels, including excavated area, mean depth, number of recovered objects, sediment volume, and ornament density (items/m³) **(S3 Table)**. Detail map of Llonín cave and other archaeological sites in the Cantabrian region and in Llonín immediate surroundings **(S1 Fig)**. Llonín cave fence, vestibule and Galería excavation area (left). Plan of the cave with the excavation sectors and rock art panels (right) **(S2 Fig)**. General view of the vestibule (Vestíbulo) **(S3 Fig)**. View of the dejection cone, including the Cono Anterior (above) and Cono Posterior (below) excavation sectors **(S4 Fig)**. General view of the stratigraphic section from Galería (above). Detail of Level III (below) **(S5 Fig)**. Stratigraphic profiles of the four excavation sectors of Llonín cave **(S6 Fig)**. Categories used to classify perforation location and morphology (A). Biometric criteria used to measure coiled and non-coiled gastropods, scaphopods, bivalves and red deer canines (B) **(S7 Fig)**. Scatter plots showing the positive correlation between root width and root thickness among hind and stag canines from Llonín cave, proving its availability for sex estimation **(S8 Fig)**.
(ZIP)

S9 Fig Images of the complete personal ornaments and raw materials assemblage of Llonín cave. (PDF)

S10 Fig Graphic representation of use-wear and ochre distribution on personal ornaments from Llonín cave. (ZIP)

S2 File Summary of the experimental collection employed during the analysis of the Llonín cave ornamental assemblage. The figure below illustrates the suspension modes employed during the use-wear experiments. Suspension durations ranged from 1,000 hours to 6 months, depending on the specimen. In total, the manufacturing experiments comprised 260 beads, whereas the use-wear experiments comprised 373 beads arranged into 126 ornaments **(S11 Fig)**. Detailed images of the manufacture traces of experimentally pierced teeth, shell and bone elements using the main techniques documented on the Llonín cave assemblage **(S12 Fig)**. Evolution of use-wear in experimental shell and tooth ornaments, evidenced by polishing, rounding, enlargement, and deformation of perforation edges. Note how use-wear progressively obscures the technological traces of manufacture **(S13 Fig)**.
(ZIP)
