# Supplementary material for: Identity and mobility through personal ornaments in Upper Paleolithic cantabrian hunter-gatherer societies: Insights from Llonín cave (Asturias, Spain)
Source: PLoS One. 2026 Jun 8;21(6):e0351170. doi: 10.1371/journal.pone.0351170 (PMC13245794; doi:10.1371/journal.pone.0351170)
Supplement: S1 File — Information regarding the ecology of the mollusk taxa identified in Llonín cave (S2 Text). Radiocarbon dates. Absolute dates for Levels III (Badegoulian), IV (Upper Solutrean) and V (Gravettian) from Galería, and Level VIa (Gravettian) from Cono Posterior. Original dates recalibrated using IntCal20 (S1 Table). List of references consulted for the creation of Fig 1. Numbers refer to the bibliography cited above (S2 Table). Summary of sectors and levels, including excavated area, mean depth, number of recovered objects, sediment volume, and ornament density (items/m³) (S3 Table). Detail map of Llonín cave and other archaeological sites in the Cantabrian region and in Llonín immediate surroundings (S1 Fig). Llonín cave fence, vestibule and Galería excavation area (left). Plan of the cave with the excavation sectors and rock art panels (right) (S2 Fig). General view of the vestibule (Vestíbulo) (S3 Fig). View of the dejection cone, including the Cono Anterior (above) and Cono Posterior (below) excavation sectors (S4 Fig). General view of the stratigraphic section from Galería (above). Detail of Level III (below) (S5 Fig). Stratigraphic profiles of the four excavation sectors of Llonín cave (S6 Fig). Categories used to classify perforation location and morphology (A). Biometric criteria used to measure coiled and non-coiled gastropods, scaphopods, bivalves and red deer canines (B) (S7 Fig). Scatter plots showing the positive correlation between root width and root thickness among hind and stag canines from Llonín cave, proving its availability for sex estimation (S8 Fig). (ZIP) [file pone.0351170.s006.zip › S1-S2 texts S1-S2 tables S1-S8 Figs.pdf]

# S1 Text. Site description.

## Llonín cave: location, chronology, and archaeological interventions

Llonín cave (UTM30 ETRS89 X:366584, Y:4798975, Z: 111.30) is located in the easternmost part of Asturias, near the eponymous village, in the municipality of Peñamellera Alta. The cavity lies on the eastern slope of one of the spurs on the southern face of the Cuera mountain range, at the confluence of the La Molinuca and El Sangual streams, both tributaries of the Cares River. It is thus situated in a west–east oriented valley, shaped by the erosive action of the river on Namurian limestone, featuring the characteristically mountainous terrain of eastern Asturias. This valley runs parallel to the coast, bounded to the south by the central and eastern massifs of the Picos de Europa and to the north by the Cuera mountain range and the Sierras Planas [1].

The cave has a single entrance through a small, covered vestibule facing ENE, which provides access to two main chambers: a narrow upper gallery approximately 30 meters long, well-suited for habitation [2], and a large, irregularly shaped lower hall around 25 meters wide and 40 meters deep. The elevation difference between the vestibule and the lower hall—about 15 meters at its lowest points—has led to the formation of a prominent dejection cone sloping north-westward.

Originally known as *Concha de la Cova*, the site was used for cheese fermentation from 1957 until its scientific discovery in 1971 by M. Berenguer Alonso, who identified its Paleolithic art and renamed it *Cueva de Llonín* [2,3]. Early studies by Berenguer [3], Gómez Tabanera [4], Apellániz [5], Almagro [6], and Aramburu [7] focused primarily on the cave's parietal art. Archaeological surveys began in 1984 under the direction of Javier Fortea, with M. de la Rasilla and V. Rodríguez joining the team in 1987. Excavations continued until 2002, though from 1999 onwards the work focused exclusively on the study, documentation, and restitution of the cave's rock art panels [8].

Prior to excavation, three main areas were designated: Vestíbulo (vestibule), Galería (gallery), and the dejection cone (Cono de Deyección)—the latter further subdivided into Cono Anterior and Cono Posterior. The goal of excavating the dejection cone was to understand the site's sedimentary dynamics, given that it was formed by material sliding down from the vestibule. Despite the displacement of materials, stratigraphic inversions were ruled out [9].

Llonín presents a broad chrono-stratigraphic sequence, including the Mousterian, Gravettian, Upper Solutrean, Badegoulian, Middle and Upper Magdalenian, and Azilian periods, along with traces of the Bronze Age. This sequence, however, is not homogeneously distributed across the four excavation sectors; due to taphonomic, sedimentary, and anthropic factors, some occupation phases are only represented in specific sectors.

**Vestíbulo**

Contrary to expectations, Vestíbulo yielded the least stratigraphic information [9]. The morphology of the cave has caused sediment and materials to migrate toward the dejection cone, as evidenced by the steep inward and outward dipping of the levels [2]. Four main stratigraphic horizons were identified. The uppermost (Level I) consists of a surface layer with mixed and imprecise chronology, underlain by a Magdalenian horizon (Levels II and III) divided into an Upper Magdalenian phase (Level II) rich in lithic and bone industry, and a phase most likely associated with the Badegoulian (Level III), with sparse archaeological remains. Level IV, rich in organic matter but thin, corresponds to the Upper Solutrean, while the underlying, nearly sterile levels are attributed to early phases of the Upper Paleolithic, likely Gravettian [2].

**Galería**

Galería provides the best-preserved spatial distribution of archaeological elements at Llonín, having remained undisturbed by sedimentary dynamics due to its elevated position, isolated from the dejection cone. Its stratigraphy includes occupational levels with hiatuses between the Mousterian, Gravettian, and Upper Solutrean, followed by a more continuous sequence from the Solutrean through the end of the Magdalenian. Post-Paleolithic levels are absent or mixed with materials of unclear chronology [2]. The Badegoulian and Upper Solutrean levels are separated by a thin continuous layer of ochre. Rasilla et al [10] suggest that this layer was sprinkled at the beginning of the occupation of level III.

Chrono-stratigraphic attributions were initially based on diagnostic fossils found throughout the site. However, recent studies [10-12] provided absolute dates for Levels IV and III of Galería, helping to chronologically contextualize the transition between the Upper Solutrean and Badegoulian. These levels, separated by the abovementioned ochre layer, have been crucial in interpreting this transition and the lack of post-depositional

alterations seen in other sites of the Cantabrian region have allowed it to be ascribed to the Badegoulian [11,13,16].

Fortea et al. [9] suggest that Galería served as a complex habitation zone during the Solutrean, later becoming a specialized activity area (e.g., hide processing, food preparation), particularly during the Badegoulian. This interpretation is based on the characterization of Level III as a generalized combustion area, with interstratified charcoal concentrations (hearths) and patches of iron oxide [9]. In addition, a substantial input of sandstone and quartz-arenite cobbles has been documented; their spatial distribution and evidence of thermal alteration suggest their use as "hot stones" for the production of heat, smoke, or steam, rather than as structural components of hearth features. The presence of numerous faunal remains, together with tools associated with hide processing (e.g., awls and needles), further supports this interpretation [9-10]. In contrast, the Solutrean occupation is characterized by a wide range of activities that appear to be extensively distributed across the four excavation sectors. These include lithic production, the processing of faunal remains, and a substantial assemblage of finished tools [9, several PhD theses in preparation]. Notably, these levels also exhibit a greater abundance and clearer evidence for the manufacture of non-utilitarian items (personal ornaments) when compared to those attributed to the Badegoulian. Finally, the spatial distribution of hearths in Level IV of the Galería sector appears to respond to the cave's topography, likely representing a deliberate strategy to optimize smoke evacuation and improve habitability conditions within the site [9].

In the upper levels (II and I) of Galería, interstratigraphic contamination was identified and isolated, originating from a large hearth excavated into the superficial level. This disturbance affected both the surface level and the upper portions of Level II within a localized area of squares B4 and C4. For further details, see Duarte et al. [17].

**Cono Anterior**

This sector features a thick stratigraphic sequence displaced from its original context by the dejection cone's dynamics, though there are no stratigraphic inversions, except for Levels I–IV, consisting of a nearly sterile sedimentary mix. The remainder of the stratigraphy appears vertically intact, with clearly differentiated levels. Levels V–VII correspond to post-Paleolithic materials (Azilian and Bronze Age), while Levels VIII–X contain a rich Magdalenian record with faunal remains, lithic industry and bone tools [2], along with the site's highest concentration of personal ornaments. Despite dense Upper

(Levels VIII–IX) and Middle Magdalenian (Level X) remains, the Lower Magdalenian and the Badegoulian are absent in this sector. A major issue here is the nature of the Magdalenian sediments from levels IX-X—stone and pebble layers without matrix—causing some profile collapses and the consequent data loss [9]. The main unclear stratigraphic correspondence comes from the contact between these two layers, whose ornaments have been classified as "Level IX-X" (S1-4 Data) and, while being undoubtedly Magdalenian, have been discarded from the analysis and interpretations.

Level XI, attributed to the Upper Solutrean, is less dense but includes a significant lithic and bone assemblage, along with several personal ornaments. The underlying Levels XII–XIV are nearly sterile [9], clearly delimiting the beginning of the Solutrean occupation of level XI.

Despite the absence of absolute dating for the Cono Anterior, the stratigraphic levels and their associated cultural phases are clearly defined by diagnostic fossils. Levels VIII and IX (Upper Magdalenian) are characterized by the presence of several single-row harpoons with quadrangular cross-sections, as well as engravings of goats depicted in frontal view on bone fragments and on the harpoons themselves [9,17]. Level X is associated with fork-based spear points, with particular emphasis on the presence of a bone disc[79], included in this study, which constitutes a clear marker of the Cantabrian Middle Magdalenian. In Level XI, lithic elements with flat retouch are abundant, including numerous concave-based points, notched points, and laurel-leaf points.

**Cono Posterior**

Located along the western cave wall, Cono Posterior represents the lowest part of the dejection cone. It was extensively excavated to better understand the stratigraphic dynamics of the cone. Some parts of the excavation area were compromised by modifications made during the cave's use for cheese maturation, nevertheless, stratigraphic continuity between the Cono Anterior and Cono Posterior confirms the absence of inversions [9].

This sector begins with a post-Paleolithic horizon (Level II) sealed beneath a stalagmitic crust (Level I). Level III is a nearly sterile sedimentary fill, contemporaneous with the Magdalenian horizon in Cono Anterior, underlain by faunal-rich Upper Solutrean deposits in Level IV. Level V, also sterile, and seals the solutrean occupation of this sector, being dated to Würm III, and followed by lower levels from the Gravettian (Level VI) and Mousterian (Level VIII), both radiocarbon dated [15,79] with an intermediate

mixed Level VII. Archaeological remains are sparse in this sector except for Level IV, though Level VIII contains a notable feature, an anthropized carnivore accumulation [14,15].

**Chrono-cultural and stratigraphic correlation across sectors**

At Llonín, variable occupation intensities are observed over time, with human activity especially intense from the Upper Solutrean through the end of the Magdalenian. The Solutrean levels show the best stratigraphic correlation, occurring in Level XI (Cono Anterior) and Level IV (Galería, Vestíbulo, and Cono Posterior). Magdalenian phases appear intermittently across sectors, with some levels —e.g., Level III of the Cono Posterior—remaining imprecisely attributed. The Badegoulian is radiocarbon dated in Level III of Galería and also associated with Level III of Vestíbulo, and the Middle Magdalenian is present in level X of the Cono Anterior and also in the lowermost part of Level II of Galería, where is partially mixed with Upper Magdalenian [17]. The Upper Magdalenian is consistently present in Level II Galería and Vestíbulo, and in Level VIII and IX of Cono Anterior.

Earlier Upper Paleolithic phases are represented by the Gravettian in Level V of Galería and Level VI-VII of Cono Posterior, although in level VII inter-stratigraphic contaminations with the underlying Mousterian layer (VIII) are documented [1,15]. Post-Paleolithic layers are heavily impacted by taphonomic processes and surface disturbance, except for Levels V–VII of Cono Anterior, where Azilian and Bronze Age occupations are detected. As for the link between stratigraphy and parietal art, Llonín is a prime example where "art and occupation are mutually correlated"[9]; graphic representations with notable superpositions in the main panel are recorded from the Gravettian through to the end of the Magdalenian [8].

# S2 Text. Information regarding the ecology of the mollusk taxa identified in Llonín cave.

## *Gastropoda*

### Genus *Littorina*

The genus *Littorina* is widely represented at Llonín by the species *Littorina littorea* (Linnaeus, 1758), *Littorina saxatilis* (Olivi, 1792), and *L. obtusata / fabalis*. *L. littorea* is a taxon with clear nutritional value, intensively consumed during the Upper Palaeolithic and the Mesolithic; it is characterised by a thick, spiralled, striated shell with a pointed apex [20]. Despite its marked intraspecific polymorphism, *L. saxatilis* shares certain features with *L. littorea*, such as a pointed apex and the presence of striated ornamentation; however, it is smaller in size, lacks nutritional value, and displays a colour range varying from brown to reddish tones, whereas *L. littorea* typically exhibits darker and more uniform coloration [63].

Although *L. obtusata* and *L. fabalis* exhibit some ecological differences, such as habitat preferences within the intertidal zone and dietary behaviour, they are morphologically indistinguishable except for their reproductive organs, a diagnostic feature not preserved in archaeological specimens [64]. For this reason, both species have been grouped under a single taxonomic category, *L. obtusata/fabalis*. These taxa are characterised by a smooth, non-umbilicate shell surface, with considerable variability in both shape and colour among individuals. As in L. littorea and L. saxatilis, the shell morphology is globular with a wide, elliptical aperture; however, in *L. obtusata/fabalis*, the apex is not prominently pointed and spiral ornamentation is absent [63,20]. These species inhabit a variety of substrates within the intertidal zone, primarily rocky areas and algal environments, feeding on algae as well as biofilm and other microalgae [65-66].

WoRMS links:

- WoRMS - World Register of Marine Species - Littorina obtusata (Linnaeus, 1758)
- WoRMS - World Register of Marine Species - Littorina fabalis (W. Turton, 1825)
- WoRMS - World Register of Marine Species - Littorina saxatilis (Olivi, 1792)
- WoRMS - World Register of Marine Species - Littorina littorea (Linnaeus, 1758)

**Genus *Trivia***

Specimens of the genus *Trivia* are characterised by a globular morphology, pale pink coloration, and a straight aperture running parallel to the longitudinal axis from the apex to the end of the columella. Both the base and dorsal surface are covered by spiral ribs that terminate along the aperture lips in the form of small denticles [63]. Two species of this genus are documented along the Cantabrian coast, *Trivia arctica* (Pulteney, 1799) and *Trivia monacha* (da Costa, 1778) [67]. However, the only distinguishing feature between them is the presence in *T. monacha* of a pattern of three dark spots on the dorsal side. The loss of pigmentation commonly observed in archaeological shells limits taxonomic identification to the genus level [68]. Both species inhabit rocky substrates in shallow littoral and sublittoral environments, where they feed primarily on marine sponges. This genus lacks nutritional value but has been widely used for the manufacture of personal ornaments from the Upper Palaeolithic to the present.

WoRMS links:

- WoRMS - World Register of Marine Species - Trivia arctica (Pulteney, 1799)
- WoRMS - World Register of Marine Species - Trivia monacha (da Costa, 1778)

**Genus *Turritellinella***

Represented by the species *Turritellinella tricarinata*, this taxon is characterised by relatively fragile shells with a distinctive turriform morphology, highly elongated and reaching up to 19 whorls in some individuals [20]. Its colour range varies from red to pale yellow, although both interspecific and intraspecific variability in colour patterns and ornamentation is observed. The whorls are markedly convex and ornamented with three keels. This species inhabits sandy and muddy substrates in subtidal environments and across the continental shelf, down to depths of approximately 200 m [69], where it feeds primarily on suspended particles. Its presence in Cantabrian contexts is relatively frequent, being documented in Palaeolithic levels at sites such as El Horno, Urtiaga, Cueto de la Mina, and Bolinkoba [20].

WoRMS links:

- WoRMS - World Register of Marine Species - Turritellinella tricarinata (Brocchi, 1814)

**Genus *Nucella***

Archaeologically, this genus is represented by *Nucella lapillus*. Its morphology varies considerably depending on environmental conditions and coastal exposure. The shell is characterised by its robustness and conical profile with a pointed apex, as well as a wide, oval aperture [63,70]. Its coloration ranges from greyish ochre tones to alternating light and dark bands. This species inhabits the intertidal zone, particularly on hard substrates covered by mussels or barnacles, upon which it feeds [71]. It is especially abundant in Asturian archaeological sites, although it is distributed along the entire Cantabrian coast, as well as in Mediterranean and Atlantic Iberian contexts.

WoRMS links:

- WoRMS - World Register of Marine Species - Nucella lapillus (Linnaeus, 1758)

**Genus *Tritia***

The genus *Tritia* is represented in the Llonín assemblage by the species *Tritia reticulata* (Linnaeus, 1758), *Tritia incrassata* (Strøm, 1768), and *Tritia mutabilis* (Linnaeus, 1758). *Tritia reticulata* is characterised by an oval-conical shape, a greyish yellow-brown coloration, and a reticulated ornamentation produced by the intersection of spiral and axial ribs [70]. *Tritia incrassata* is generally smaller, with more convex whorls and a more rounded aperture. Both species are scavengers and detritivores, inhabiting sandy or rocky substrates in intertidal and subtidal environments in both the Atlantic and the Mediterranean [63]. In contrast, *Tritia mutabilis* displays a smooth, non-reticulated shell of relatively low thickness, with a large initial whorl and a wide aperture. Its colour range varies from light brown to yellow. This species is characteristic of the Mediterranean Sea and inhabits sandy and muddy substrates at greater depths than *T. reticulata* and *T. incrassata*, while sharing a scavenging and detritivorous feeding behaviour.

WoRMS links:

- WoRMS - World Register of Marine Species - Nucella lapillus (Linnaeus, 1758)
- WoRMS - World Register of Marine Species - Tritia incrassata (Strøm, 1768)
- WoRMS - World Register of Marine Species - Tritia mutabilis (Linnaeus, 1758)

### Genus *Patella*

The species represented are *Patella vulgata* (Linnaeus, 1758) and *Patella depressa* (Pennant, 1777), both characterised by a robust constitution and a conical shape, with the apex slightly displaced from the centre along the longitudinal axis. They are primarily distinguished by rib thickness and internal coloration: entirely yellow in *P. vulgata*, and marked by concentric red or black and light bands in *P. depressa* [72]. These molluscs exhibit marked intraspecific variability influenced by factors such as wave exposure and desiccation [72]. Both species inhabit rocky substrates in the intertidal zone [63], where they feed primarily on algae and biofilm.

WoRMS links:

- WoRMS - World Register of Marine Species - Tritia mutabilis (Linnaeus, 1758)
- WoRMS - World Register of Marine Species - Patella depressa Pennant, 1777

## *Bivalvia*

### Genus *Glycymeris*

This genus comprises equivalve and equilateral bivalves, characterised by their robust and only slightly convex shells, as well as a brown external surface contrasting with a nacreous white interior [20]. Specimens rarely exceed 6–7 cm in length and inhabit shallow subtidal sandy or muddy substrates, where they feed on suspended particles through filter-feeding. This genus is among the most commonly used bivalves for the manufacture of personal ornaments along both the Atlantic and Mediterranean coasts during the Palaeolithic, with *Glycymeris glycymeris* (Linnaeus, 1758) and *Glycymeris nummaria* (Linnaeus, 1758) being two of the most frequently exploited species for this purpose.

WoRMS links:

- WoRMS - World Register of Marine Species - Glycymeris da Costa, 1778

### Genus *Chlamys*

The genus Chlamys is documented in the Llonín assemblage through the species *Chlamys islandica* (Müller, 1776). It is characterised by two identical but individually asymmetrical valves, such that the right auricle of each valve displays a deeper notch than the left [73]. The shell surface, which exhibits a wide range of colours—from white to red, yellow, or violet—is only slightly convex and marked by numerous radial ribs, whose

abundance allows it to be distinguished from the morphologically similar taxon *Mimachlamys varia* (Linnaeus, 1758). This species is commonly used as a palaeoclimatic indicator, as its ecological niche is restricted to very cold waters; at present, it is found exclusively in Arctic seas [53]. It inhabits infratidal environments, particularly at depths between 15 and 60 m, on substrates composed of gravel, sand, shells, or stones [73].

Although *C. islandica* is recorded in Magdalenian and Solutrean levels at numerous sites such as Aitzbitarte IV, El Castillo, El Pendo, Cueto de la Mina, Altamira, and Balmori, its use as a raw material for ornament manufacture in the Iberian Peninsula appears to be limited to a single specimen from Santa Catalina [53]. In contrast, personal ornaments made of *C. islandica* have been documented at French sites such as Isturitz, Bedeilhac, Enlène, and La Vache [74].

WoRMS links:

- WoRMS - World Register of Marine Species - Chlamys islandica (O. F. Müller, 1776)

### ***Veneridae* family**

Members of the family *Veneridae* are characterised by equivalve shells with an asymmetrical oval shape and robust hinges bearing well-developed teeth. Their preferred habitat is the intertidal zone on sandy and silty substrates, although some species occur at greater depths in subtidal environments [63]. Molluscs of this family were intensively exploited for consumption, particularly during the Mesolithic; however, their use as raw material for the manufacture of personal ornaments is uncommon in the Iberian Peninsula.

WoRMS links:

- WoRMS - World Register of Marine Species - Veneridae Rafinesque, 1815

## ***Scaphopoda*:**

### **Genus *Antalis***

Specimens of the genus *Antalis* possess tubular, slightly curved shells with openings at both ends, commonly referred to as “tusk shells.” Shell length, thickness, and ornamentation vary depending on the species, although precise taxonomic attribution is often hindered by poor surface preservation due to natural agents such as marine abrasion [20]. These organisms inhabit sandy or muddy seabeds in subtidal environments [63], feeding on microorganisms and suspended organic matter. Species within this genus have

been widely and recurrently used as personal ornaments across a broad range of chronological periods and geographical contexts.

WoRMS links:

- WoRMS - World Register of Marine Species - Antalis H. Adams & A. Adams, 1854

## *Polychaeta:*

***Rotularia* sp.**

This taxon corresponds to a fossil polychaete, specifically a tube-dwelling serpulid annelid, characterised by a tubular morphology with a relatively regular planispiral coiling [75]. Its chronological range remains debated: some authors place it between the Upper Jurassic (Kimmeridgian, ca. 157 Ma) and the Upper Eocene-Oligocene (ca. 34 Ma) [76-77], whereas others restrict its origin to the Lower Palaeocene (Danian, ca. 66 Ma) [78]. At a regional scale, this fossil species is found in Eocene deposits of the Aquitaine Basin, the South Pyrenean region of Vic, and the Jaca and Pamplona basins, as well as in certain areas of the Basque-Cantabrian Basin [75].

# TABLES

**S1 Table. Radiocarbon dates.** Absolute dates for Levels III (Badegoulian), IV (Upper Solutrean) and V (Gravettian) from Galería, and Level VIa (Gravettian) from Cono Posterior. Original dates [11-12,22-23] recalibrated using IntCal20 [24].

| ID | Origin | Lab. Ref. | BP Date | SD | Material | Method | Cal BP 2σ (95.4%) | Period |
|---|---|---|---|---|---|---|---|---|
| LLO-71 | GA.III | OxA-26042 | 17480 | 75 | Ericacea | AMS | 21366-20902 | Badegoulian |
| LLO-70 | GA.III | OxA-26041 | 17610 | 90 | Ericacea | AMS | 21712-21549 (6.8%)<br>21505-20955 (88.6%) | Badegoulian |
| LLO-68 | GA.III | OxA-26338 | 17650 | 130 | Juniperus | AMS | 21829-20950 | Badegoulian |
| LLO-71 | GA.III | OxA-26340 | 17920 | 80 | Juniperus | AMS | 22033-21445 | Badegoulian |
| LLO-31 | GA.III | OxA-X-2559-11 | 18100 | 90 | Bone | AMS | 22286-21814 | Badegoulian |
| LLO-69 | GA.III | OxA-26339 | 18345 | 75 | Fabacea | AMS | 22435-22123 | Badegoulian |
| LLO-28 | GA.IV | OxA-22700 | 19300 | 110 | Bone | AMS | 23734-23532 (20.7%)<br>23429-22976 (74.7%) | U. Solutrean |
| LLO-27 | GA.IV | OxA-22699 | 19330 | 100 | Bone | AMS | 23737-23526 (24.3%)<br>23436-23001 (71.2%) | U. Solutrean |
| LLO-26 | GA.IV | OxA-22698 | 19480 | 110 | Bone | AMS | 23779-23145 | U. Solutrean |
| LLO-81 | GA.V | OxA-X-2640-48 | 28390 | 350 | Bone | AMS | 33668-31688 | Gravettian |
| LLO-100 | CP.VIa | ANU70306 | 29231 | 387 | Bone | AMS | 34528-32805 | Gravettian |

**S2 Table. List of references consulted for the creation of Fig 1.** Numbers refer to the bibliography cited above.

| SITE | REFERENCES |
|---|---|
| Abauntz | [20] and references therein |
| Aitzbitarte III y IV | [20] and references therein |
| | [25] |
| Alkerdi | [20] and references therein |
| Altamira | [20] and references therein |
| | Under study by A. León Castelao |
| Amalda | [20] and references therein |
| Antoliña | [20] and references therein |
| | [26] |
| Arlanpe | [27] |
| Balmori | [20] and references therein |
| Berroberria | [20] and references therein |
| Bolinkoba | [20] and references therein |
| | [28,29] |
| Chufin | [20] and references therein |
| Coimbre | [30] |
| Collubil | [20] and references therein |
| | [31] |
| Cova Rosa | [20] and references therein |
| | [32] |
| Cualventi | [33] |
| Cueto de la Mina | [20] and references therein |
| El Horno | [20] and references therein |
| | [34] |
| Cueva Morin | [20] and references therein |
| C. Oscura de Ania | [20] and references therein |
| | [35] |
| C. Oscura de Perán | [36] |
| El Buxu | [20] and references therein |
| El Castillo | [20] and references therein |
| El Cierro | [20] and references therein |
| | [37] |
| El Juyo | [20] and references therein |
| El Linar | [20] and references therein |
| | [33] |
| El Miron | [20] and references therein |
| | [38] |
| | Under study by A. León Castelao |
| El Olivo | [39] |
| El Otero | [20] and references therein |
| El Pendo | [20] and references therein |
| | [40] |
| El Pielago I y II | [20] and references therein |
| El Ruso I | [20] and references therein |
| | [41] |
| El Sofoxo I | [20] and references therein |
| El Valle | [20] and references therein |
| Entrefoces | [20] and references therein |
| Ermittia | [20] and references therein |
| | [42] |
| Erralla | [20] and references therein |
| Hornos de la Peña | [20] and references therein |
| | [43] |
| Iruroin | [20] and references therein |
| Juan de Covera | [20] and references therein |
| La Ancenia | [20] and references therein |
| La Chora | [20] and references therein |
| | Under study by O. Montero Buch |
| La Fragua | [21] |
| | Under study by C. Martínez Cagigas |
| La Garma | [20] and references therein |
| | [44-46] |
| La Güelga | [20] and references therein |
| | [47] |
| La Lloseta | [20] and references therein |
| La Lluera I | [48] |
| La Paloma | [20] and references therein |
| La Peña de Candamo | [20] and references therein |
| La Pila | [20] and references therein |
| La Riera | [20] and references therein |
| La Venta del Cuco | [20] and references therein |
| La Viña | [20] and references therein |
| | [1] |
| | Under study by A. León Castelao |
| Laminak II | [20] and references therein |
| Las Aguas | [33] |
| Las Caldas | [20] and references therein |
| | [49-50] |
| Les Xanes | [20] and references therein |
| | [51] |
| Leginpea | [20] and references therein |
| Llonín | [20] and references therein |
| | This study |
| Los Azules I | [20] and references therein |
| | [52] |
| Los Canes | [20] and references therein |
| Lumentxa | [53] |
| Praile Aitz I | [20] and references therein |
| | [54-55] |
| Rascaño | [20] and references therein |
| San Gregorio | [20] and references therein |
| Santa Catalina | [53] |
| Santimamiñe | [20] and references therein |
| | [56-57] |
| Silibranka | [20] and references therein |
| Tito Bustillo | [20] and references therein |
| | [58-61] |
| Urtiaga | [20] and references therein |
| | [62] |
| Zatoya | [20] and references therein |

**S3 Table. Summary of sectors and levels, including excavated area, mean depth, number of recovered objects, sediment volume, and ornament density (items/m³).**

| Galería | | | | | | |
|---|---|---|---|---|---|---|
| Level | Chronology | Excavated area ($m^2$) | Avg. depth (m) | Ornament count | Sediment volume ($m^3$) | Ornament density (items/$m^3$) |
| II | MM | 4 | 0.14 | 5 | 0.56 | 8.93 |
| III | BAD | 4 | 0.40 | 17 | 1.60 | 10.63 |
| IV | SS | 4 | 0.18 | 28 | 0.72 | 38.89 |
| **Vestibulo** | | | | | | |
| Level | Chronology | Excavated area ($m^2$) | Avg. depth (m) | Ornament count | Sediment volume($m^3$) | Ornament density (items/$m^3$) |
| II+IIA | MS | 5.5 | 0.145 | 4 | 0.80 | 5.02 |
| IIB | MM | 5.5 | 0.035 | 1 | 0.19 | 5.19 |
| III | BAD | 5.5 | 0.22 | 5 | 1.21 | 4.13 |
| IV | SS | 7 | 0.14 | 2 | 0.98 | 2.04 |
| **Cono Anterior** | | | | | | |
| Level | Chronology | Excavated area ($m^2$) | Avg. depth (m) | Ornament count | Sediment volume($m^3$) | Ornament density (items/$m^3$) |
| VIII | MS | 12 | 0.05 | 8 | 0.60 | 13.33 |
| IX | MS | 12 | 0.30 | 2 | 3.60 | 0.56 |
| X | MM | 12 | 0.25 | 86 | 3.00 | 28.67 |
| XI | SS | 12 | 0.44 | 67 | 5.28 | 12.69 |
| **Cono Posterior** | | | | | | |
| Level | Chronology | Excavated area ($m^2$) | Avg. depth (m) | Ornament count | Sediment volume($m^3$) | Ornament density (items/$m^3$) |
| IV | SS | 12.5 | 0.23 | 5 | 2.88 | 1.74 |

## REFERENCES


1. de la Rasilla M, Duarte E, Sanchís A, Carrión Y, Cañaveras JC, Marín-Arroyo AB, et al. 2020. Environment and subsistence strategies at La Viña rock shelter and Llonin cave (Asturias, Spain) during MIS3. *J Archaeol Sci Rep* **30**:1–17.
2. Fortea FJ, de la Rasilla M, Rodríguez V. 1992. La cueva de Llonín (Llonín, Peñamellera Alta). Campañas de 1987 a 1990. *Excav Arqueol Asturias* **2**:9–18.
3. Berenguer M. 1979. *El arte parietal prehistórico de la Cueva de Llonín*. (Caja de Ahorros de Asturias).
4. Gómez-Tabanera JM. 1979. El arte prehistórico de la cueva de Llonín (Peñamellera Alta. Alles) y la lógica de la conexión de los símbolos de la Prehistoria y la Etnografía astures. *Bol Inst Estud Astur* **96**–**97**:421–444.
5. Apellániz JM. 1980. El método de determinación de autor en el Cantábrico. Los grabados de Llonín. In *Altamira Symposium*. (Ministerio de Cultura), pp. 73–84.
6. Almagro M. 1980. Los grabados de trazo múltiple en el arte cuaternario español. In *Altamira Symposium*. (Ministerio de Cultura), pp. 27–71.
7. Aramburu FJ. 1984. Contribución al estudio espacial del Paleolítico Superior cantábrico: El caso asturiano. *Arqueol Espac* **2**:181–191.
8. Fortea FJ, de la Rasilla M, Rodríguez V. 2007. La Cueva de Llonín (Llonín, Peñamellera Alta). Campañas de 1999 a 2002. *Excav Arqueol Asturias* **5**:77–86.
9. Fortea FJ, de la Rasilla M, Rodríguez V. 1995. La cueva de Llonín (Llonín, Peñamellera Alta). Campañas de 1991 a 1994. *Excav Arqueol Asturias* **3**:33–43.
10. de la Rasilla M, Duarte E, Aura JE, Sanchís A, Carrión Y, Pérez-Ripoll M, et al. 2019. The Llonin Cave (Peñamellera Alta, Asturias, Spain), level III (Galería): techno-typological characterisation of the Badegoulian lithic and bone assemblages. In M. Deschamps, et al., eds. *La conquête de la montagne: des premières occupations humaines à l'anthropisation du milieu. Actes du 142e Congrès du CTHS*. (Éditions du Comité des travaux historiques et scientifiques, Paris).
11. Aura JE, Tiffagom M, Jordá-Pardo JF, Duarte E, Fernández de la Vega J, Santamaria D, et al. 2012. The Solutrean–Magdalenian transition: a view from Iberia. *Quat Int* **272**–**273**:75–87.

12. Aura JE, et al. 2014. New dates for the Iberian Badegoulian: data from Cantabrian and Mediterranean regions. In *Abstracts Book XVII World UISPP Congress*. (Fundación Atapuerca), pp. 250–251.

13. de la Rasilla M. 1989. El solutrense y magdaleniense en la región cantábrica. *Gallaecia Rev Arqueol Antigüedad* **14**:103–111.

14. Fortea JF, de la Rasilla M, Rodríguez V. 1999. La cueva de Llonín (Llonín, Peñamellera Alta). Campañas de 1995 a 1998. *Excav Arqueol Asturias* **4**:59–68.

15. Sanchis A, Real C, Sauqué V, Núñez-Lahuerta C, Égüez N, Tormo C, et al. 2019. Neanderthal and carnivore activities at Llonin Cave, Asturias, Northern Iberian Peninsula: faunal study of Mousterian levels (MIS3). *C R Palevol* **18**:113–141.

16. Ducasse S, Chauvière FX, Pétillon JM. 2021. Breaking bad? Discarding the Solutrean norms: chronology, evolution and geographical extent of the Badegoulian phenomenon in Western Europe. *Quat Int* **581**:61–83.

17. Duarte E, de la Rasilla M. 2020. The bone industry collections from the Middle and Upper Magdalenian levels in La Viña rock shelter and Llonin cave (Asturias, Northern Spain). In L. G. Straus, M. Langlais, eds. *Magdalenian ChronoStratigraphic Correlations and Cultural Connections between Cantabrian Spain and Southwest France...and beyond. Session XVII-2 of the XVIII UISPP Congress*. (Société préhistorique française, Paris), pp. 319–356.

18. Taborin Y. 1993. Traces de façonnage et d’usage sur les coquillages perforés. In P. C. Anderson, S. Beyries, M. Otte, H. Plisson, eds. *Traces et fonction: les gestes retrouvés Vol. I.* (Service de Préhistoire), pp. 255–267.

19. Tatá F, Cascalheira J, Marreiros J, Pereira T, Bicho N. 2014. Shell bead production in the Upper Paleolithic of Vale Boi (SW Portugal): an experimental perspective. *J Archaeol Sci* **42**:29–41.

20. Álvarez-Fernández E. 2006. *Los objetos de adorno-colgantes del Paleolítico superior y del Mesolítico en la Cornisa Cantábrica y en el Valle del Ebro: una visión europea*. PhD thesis. (Universidad de Salamanca).

21. Gutiérrez-Zugasti I. 2009. *La explotación de moluscos y otros recursos litorales en la región cantábrica durante el Pleistoceno final y el Holoceno inicial*. PhD thesis. (Universidad de Cantabria).

22. Marín-Arroyo AB, Rios-Garaizar J, Straus LG, Jones JR, de la Rasilla M, González-Morales MR, et al. 2018. Chronological reassessment of the Middle to Upper Paleolithic transition and Early Upper Paleolithic cultures in Cantabrian Spain. *PLoS ONE* **13**:e0194708.

23. Sanchis A, Duarte E, Pérez L, Real C, Gómez-Olivencia A, Pastor F, et al. 2025. Morphometry of a partial Late Pleistocene dhole (Cuon alpinus europaeus (Bourguignat, 1868); Carnivora, Canidae) skeleton from Llonín Cave (Asturias, Spain) and its taphonomic origin. *Geobios* **88**:227–240.

24. Reimer PJ, Austin WEN, Bard E, Bayliss A, Blackwell PG, Bronk-Ramsey C, et al. 2020. The IntCal20 Northern Hemisphere radiocarbon age calibration curve (0–55 cal kBP). *Radiocarbon* **62**:725–757.

25. Álvarez-Fernández E. Estudio de los moluscos y los crustáceos del yacimiento de Aitzbitarte III (zona de entrada). In: Ocupaciones humanas en Aitzbitarte III (País Vasco) 33,600–18,400 BP: Zona de entrada a la cueva. Vitoria-Gasteiz: Gobierno Vasco; 2011. p. 517–528.

26. Aguirre-Ruiz de Gopegui M. Ocupaciones gravetienses de Antoliñako koba: aproximación preliminar a su estratigrafía, cronología e industrias. In: Pensando en el Gravetiense: nuevos datos para la región cantábrica en su contexto peninsular y pirenaico. Madrid: Ministerio de Educación, Cultura y Deporte; 2012. p. 216–228.

27. Rios-Garaizar J, Maidagan DG, Olivencia AG, Alonso DA, Aviles EI, Moreno AG, et al. El final del Solutrense en el Oriente Cantábrico a través de las ocupaciones de la cueva de Arlanpe (Lemoa, Vizcaya). Zephyrus. 2013;72:15–38.

28. Iriarte-Chiapusso MJ, Arrizabalaga A. El yacimiento arqueológico de Bolinkoba (Abadiño, Bizkaia): crónica de las investigaciones en la cavidad, secuencia estratigráfica y cronología numérica. Kobie Ser BAI. 2015;6:5–88.

29. Álvarez-Fernández E. Las conchas marinas perforadas de Bolinkoba (Abadiño, Bizkaia). Kobie. 2015;6:159–164.

30. Álvarez-Fernández E. Los objetos de adorno de la zona B de la cueva de Coímbre (Asturias, España). In: La cueva de Coímbre (Peñamellera Alta, Asturias): ocupaciones humanas en el valle del Cares durante el Paleolítico superior. Oviedo: Fundación María Cristina Masaveu Peterson; 2017. p. 458–469.

31. González-Morales MR. El colgante decorado paleolítico de la cueva de Collubil (Amieva, Asturias). Bol Inst Estud Asturianos. 1974;28(83):837–842.

32. Álvarez-Fernández E, Andrés M, Aparicio MT, Elorza M, Gabriel S, García-Ibaibarriaga N, et al. Biotic resources in the Lower Magdalenian at Cova Rosa (Sardeu, Asturias, Cantabrian Spain). Quat Int. 2019;506:25–34.

33. De las Heras-Martín C. Objetos grabados y elementos de adorno de los yacimientos de Cualventi, El Linar y Las Aguas (Alfoz de Lloredo, Cantabria): excavaciones de 2003 a 2005. In: Proyecto de investigación "Los tiempos de Altamira": actuaciones arqueológicas en las cuevas de Cualventi, El Linar y Las Aguas (Cantabria, España). Madrid: Subdirección General de Documentación y Publicaciones; 2016. p. 645–688.

34. Fano MA, Álvarez-Fernández E. Magdalenian marine shells from El Horno Cave (Ramales, Cantabria, Spain) in the regional context. In: Not only food: Marine, terrestrial and freshwater molluscs in archaeological sites. San Sebastián: Sociedad de Ciencias Aranzadi; 2010. p. 58–68.

35. Adán-Álvarez GE, García Sánchez E, Quesada López JM. Avance al estudio del Magdaleniense de Cueva Oscura de Ania (Las Regueras, Asturias, España). Caesaraugusta. 2007;78:91–106.

36. Adán-Álvarez GE. De la caza al útil: la industria ósea del Tardiglaciar en Asturias. Oviedo: Servicio Central de Publicaciones del Principado de Asturias; 1997.

37. Álvarez-Fernández E, Arias P, Bécares J, Cubas M, Elorza M, Gabriel S, et al. Intervenciones arqueológicas en la cueva de El Cierro (Fresnu, Ribadesella, Asturias, España): síntesis de los datos disponibles procedentes de los recientes trabajos realizados en tres zonas del yacimiento. Entemu. 2022;19.

38. Gutiérrez-Zugasti I, Cuenca-Solana D. Ornaments from the Magdalenian burial area in El Mirón Cave (Cantabria, northern Spain): were they grave goods? J Archaeol Sci. 2015;60:112–124.

39. Álvarez-Alonso D, De Andrés M, Álvarez-Fernández E, García-Ibaibarriaga N, Jordá-Pardo FJ, Rojo J. Los campamentos secundarios en el Magdaleniense cantábrico: resultados preliminares de la excavación en la cueva del Olivo (Llanera, Asturias). In: Cien años de arte rupestre paleolítico: centenario del descubrimiento de

la cueva de la Peña de Candamo (1914–2014). Salamanca: Universidad de Salamanca; 2014. p. 359–368.

40. Álvarez-Fernández E, Armenteros I, Barba P, Camarós E, Cueto M. La esteatita en la cueva de El Pendo (Escobedo de Camargo, Cantabria): caracterización geológica y funcionalidad. In: Gárate D, González Urquijo JE, editors. Reflejos de una sociedad: estudios sobre el Paleolítico superior en homenaje al profesor César González Sainz. Santander: Ediciones Tantín; 2026.
41. Álvarez-Fernández E, Fernández García R. Marine resources exploitation in Cantabrian Spain during the Solutrean: molluscs, fish and sea mammals. Bull Mus Anthropol Préhist Monaco. 2012;51:87–97.
42. Erostarbe A. Reevaluación de la secuencia paleolítica de la excavación antigua de Ermittia: la contribución del análisis tecnológico de las materias duras animales [Master's thesis]. Vitoria-Gasteiz: Universidad del País Vasco; 2018.
43. Ríos-Garaizar J, Maíllo-Fernández JM, Marín-Arroyo AB, Sánchez-Carro MA, Salazar S, Medina-Alcaide MA, et al. Revisiting Hornos de la Peña 100 years after. J Archaeol Sci Rep. 2020;31:102259.
44. Peñalver E, et al. Local amber in a Palaeolithic context in Cantabrian Spain: the case of La Garma A. J Archaeol Sci. 2007;34(6):843–849.
45. Martín-Jarque S, Tarriño A, Delclòs X, García-Alonso B, Peñalver E, Prieto A, et al. Les silex et autres matières premières comme preuves de contacts entre les groupes de chasseurs-cueilleurs pendant le Paléolithique supérieur de la région cantabrique (nord de l'Espagne): synthèse de l'information disponible. L'Anthropologie. 2023;127(1):103092.
46. Álvarez-Fernández E. Investigaciones arqueomalacológicas en La Garma A (Omoño, Cantabria): los moluscos marinos de los niveles N y O (Magdaleniense superior). In: Arias Cabal P, Corchón Rodríguez MS, Menéndez Fernández M, Rodríguez Asensio JA, editors. El Paleolítico Superior Cantábrico: actas de la Primera Mesa Redonda (San Román de Candamo, Asturias, 26–28 de abril de 2007). Santander: Universidad de Cantabria; 2012. p. 145–156.

47. Menéndez M, García E, Quesada JM. Excavaciones en la cueva de La Güelga (Narciandi, Cangas de Onís): campañas de 1999–2002. Excav Arqueol Astur. 2006;5:63–75.

48. Rodríguez-Asensio JA, Barrera-Logares JM. Las ocupaciones solutrenses de las cuevas de La Lluera. In: Excavaciones arqueológicas en Asturias 2007–2012: en el centenario del descubrimiento de la caverna de la Peña de Candamo. Oviedo: Consejería de Educación, Cultura y Deporte; 2013. p. 87–108.

49. Álvarez-Fernández E. The marine molluscs of Las Caldas cave (San Juan de Priorio, Oviedo). In: Corchón S, editor. Las cuevas de Las Caldas (Priorio, Oviedo): ocupaciones magdalenienses en el valle del Nalón. Salamanca: Universidad de Salamanca; 2017. p. 247–556.

50. Corchón S, Ortega P. Los niveles solutrenses de la Sala I de la cueva de Las Caldas (25,000–21,000 cal BP): industrias y arte mueble. In: Corchón S, editor. Las cuevas de Las Caldas (Priorio, Oviedo): ocupaciones magdalenienses en el valle del Nalón. Salamanca: Universidad de Salamanca; 2017. p. 247–556.

51. Álvarez-Fernández E, Martínez-Villa A, Cerezo-Fernández R, Cubas M, Cueto M, García-Alonso B, et al. Nuevos datos sobre la prehistoria en el valle del Nalón: la cueva de Les Xanes (Olloniego, Oviedo, Asturias, España). Entemu. 2024;20:169–203.

52. Fernández-Irigoyen J. Los moluscos marinos del yacimiento de la cueva de Los Azules: recurso bromatológico y elemento ornamental y simbólico. In: Ad Orientem: del final del Paleolítico en el norte de España a las primeras civilizaciones del Oriente Próximo: estudios en homenaje a Juan Antonio Fernández-Tresguerres Velasco. Oviedo: Servicio de Publicaciones; 2012. p. 91–108.

53. Berganza E, Arribas JL, Idarraga RR. Estudio tecnológico de los moluscos marinos perforados de los yacimientos de Lumentxa y Santa Catalina (Lekeitio, Bizkaia). Munibe. 2012;63:91–102.

54. Peñalver X, San José S, Mujika-Alustiza JA, editors. La cueva de Praileaitz I (Deba, Gipuzkoa, Euskal Herria): intervención arqueológica 2000–2009. San Sebastián: Sociedad de Ciencias Aranzadi; 2017.

55. D’Errico F, Vanhaeren M, Queffelec A. Les galets perforés de Praileaitz I (Deba, Gipuzkoa). In: Peñalver X, San José S, Mujika-Alustiza JA, editors. La cueva de Praileaitz I (Deba, Gipuzkoa, Euskal Herria): intervención arqueológica 2000–2009. San Sebastián: Sociedad de Ciencias Aranzadi; 2017. p. 453–484.

56. González-Sainz C. Industrias en hueso y asta de los niveles magdalenienses de Santimamiñe (excavaciones 2004–2007). Kobie. 2011;1:111–154.

57. Gutiérrez-Zugasti I, Cuenca-Solana D, Clemente-Conte I, González-Sainz C, López-Quintana JC, et al. Instrumentos de trabajo y elementos de adorno en conchas de moluscos de la cueva de Santimamiñe (Kortezubi, Bizkaia). Kobie. 2011;1:155–170.

58. Álvarez-Fernández E, Tapia J, Aguirre-Uribesalgo A, Arias P, Camarós E, Cerezo-Fernández et al. La cueva de Tito Bustillo (Ardines, Ribadesella, Asturias, España): intervenciones arqueológicas en el Área de Estancia. Entemu. 2022;19:247–265.

59. Álvarez-Fernández E. La récolte des coquillages dans la région cantabrique au Magdalénien: la grotte de Tito Bustillo (Asturies, Espagne). L’Anthropologie. 2013;117(1):62–93.

60. Álvarez-Fernández E, Cueto M, Tapia J, Aparicio MT, Douka K, Elorza M, et al. Nuevos datos cronoestratigráficos y arqueozoológicos de la cueva de Tito Bustillo-Área de Estancia (Ribadesella, Asturias). Kobie (Anejo). 2018;18:109–122.

61. Álvarez-Fernández E, Martín-Jarque S, Tarriño A. 2021. Flint & Shell: raw materials as evidence of long-distance contacts in Cantabrian Spain during the Magdalenian. En: Gauzinski-Windheuser S, Jöris O (eds.) The Beef behind all Possible Pasts – The Tandem Festschrift in Honour of Elaine Turner and Martin Street. Mainz: Römisch-Germanisches Zentralmuseum; pp. 215–235.

62. Altuna J, Mariezkurrena K. Moluscos marinos de la cueva de Urtiaga (Deba, Gipuzkoa). Kobie Paleoantropol. 2010;29:47–63.

63. Palacios-Egüen N, Vega de la Torre JJ. Guía de conchas de las playas y rías de Cantabria. Santander: Gobierno de Cantabria; 1997.

64. Sotelo G, Duvertorp M, Costa D, Panova M, Johannesson K, Faria R. Phylogeographic history of flat periwinkles, *Littorina fabalis* and *Littorina obtusata*. *BMC Evol Biol*. 2020;20:1–18.

65. Pizzolla PF. Littorina obtusata (common flat periwinkle). In: Tyler-Walters H, Hiscock K, editors. Marine Life Information Network: Biology and Sensitivity Key Information Reviews [Internet]. Plymouth: Marine Biological Association of the United Kingdom; 2008 [cited 2026 Mar 3]. Available from: https://www.marlin.ac.uk/species/detail/1487

66. Ballerstedt S. Littorina saxatilis (rough periwinkle). In: Tyler-Walters H, Hiscock K, editors. Marine Life Information Network: Biology and Sensitivity Key Information Reviews [Internet]. Plymouth: Marine Biological Association of the United Kingdom; 2007 [cited 2026 Mar 3]. Available from: https://www.marlin.ac.uk/species/detail/1649

67. Rigaud S, Gutiérrez-Zugasti I. Symbolism among the last hunter–fisher–gatherers in northern Iberia: personal ornaments from El Mazo and El Toral III Mesolithic shell midden sites. Quat Int. 2016;407:131–144.

68. Dommergues E, Dommergues JL, Dommergues CH. Deux espèces sous un même masque: le point de vue paléontologique piégé par les coquilles de deux espèces européennes de Trivia (Mollusca, Gastropoda). Rev Paleobiol. 2006;25:775–790.

69. Carter MC. Turritellinella tricarinata Auger shell. In: Tyler-Walters H, Hiscock K, editors. Marine Life Information Network: Biology and Sensitivity Key Information Reviews [Internet]. Plymouth: Marine Biological Association of the United Kingdom; 2008 [cited 2026 Mar 3]. Available from: https://www.marlin.ac.uk/species/detail/1950

70. Dupont C, Gruet Y. Variations morphologiques de mollusques gastropodes (Nucella lapillus et Hinia reticulata): intérêts pour l'archéologie. ArcheoSciences. 2000;24(1):53–61.

71. Crothers JH. Dog-whelks: an introduction to the biology of Nucella lapillus (L.). Field Stud. 1985;6:291–360.

72. Madariaga B. La Cueva del Otero (Estudio paleontológico). Madrid: Dirección General de Bellas Artes; 1966.

73. Wiborg KF. Some observations on the Iceland scallop, Chlamys islandica (Müller), in Norwegian waters. Rep Norw Fish Mar Invest. 1963;13:38–53.

74. Taborin Y. La parure en coquillage au Paléolithique. Paris: CNRS; 1993.

75. Elorza J, Astibia H. Fosildiagénesis del anélido Rotularia spirulaea (Lamarck, 1818) (Polychaeta, Serpulidae) en el Eoceno del dominio pirenaico occidental. Span J Palaeontol. 2018;33(2):299–320.

76. Jäger M. Serpulidae und Spirorbidae (Polychaeta sedentaria) aus Campan und Maastricht von Norddeutschland, den Niederlanden, Belgien und angrenzenden Gebieten. Geol Jahrb A. 2005;157:121–149.

77. Savazzi E. Morphology and mode of life of the polychaete Rotularia. Paläontol Z. 1995;69:73–85. doi:10.1007/BF02985975

78. Ippolitov AP, Vinn O, Kupriyanova EK, Jäger M. Written in stone: history of serpulid polychaetes through time. Mem Mus Vic. 2014;71:123–159.

79. Fortea FJ, De La Rasilla M, Rodríguez V. Sobre un rodete perforado magdaleniense de Llonín (Asturias). Arch Prehist Levant 1990;20:95–108.

# S1-S8 Figures

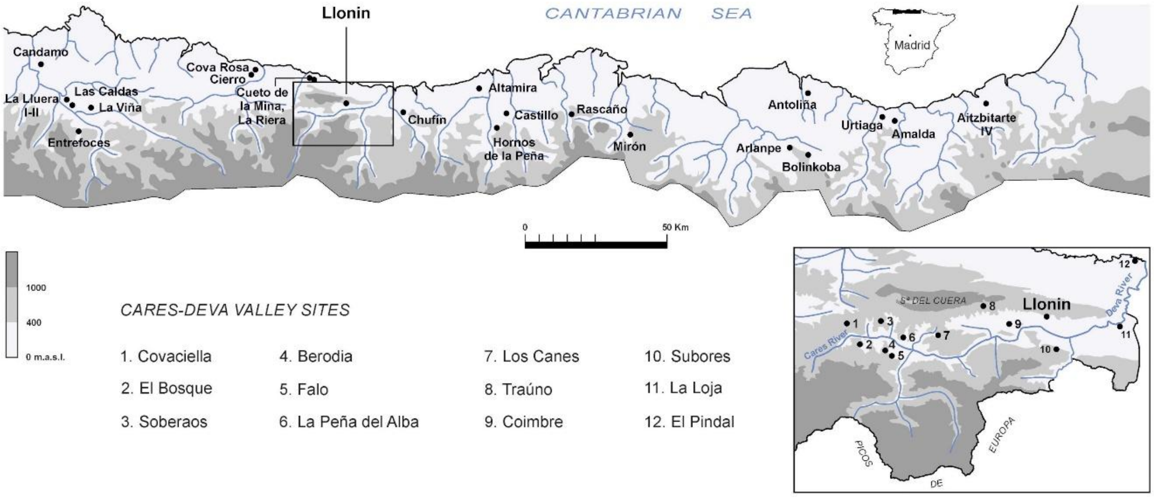


**S1 Fig.** Detail map of Llonín cave and other archaeological sites in the Cantabrian region and in Llonín immediate surroundings Photo: Elsa Duarte.

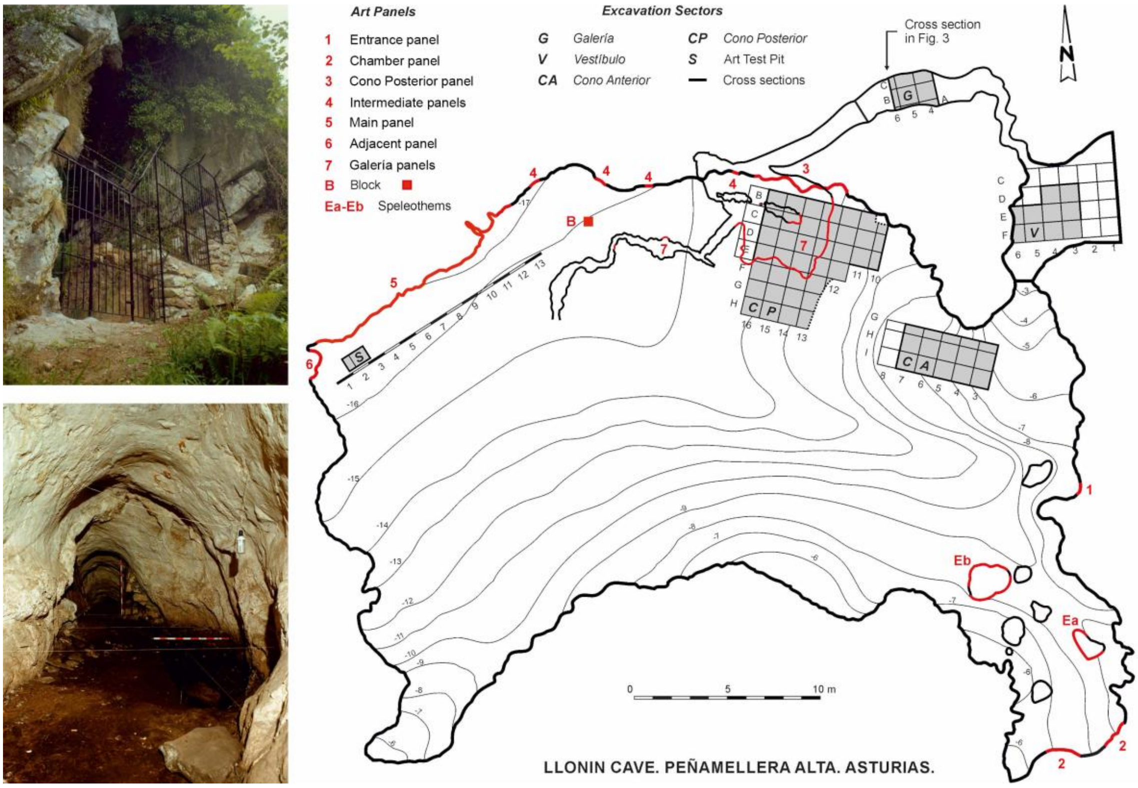


**S2 Fig.** Llonín cave fence, vestibule and Galería excavation area (left). Plan of the cave with the excavation sectors and rock art panels (right). Photos: Javier Fortea.

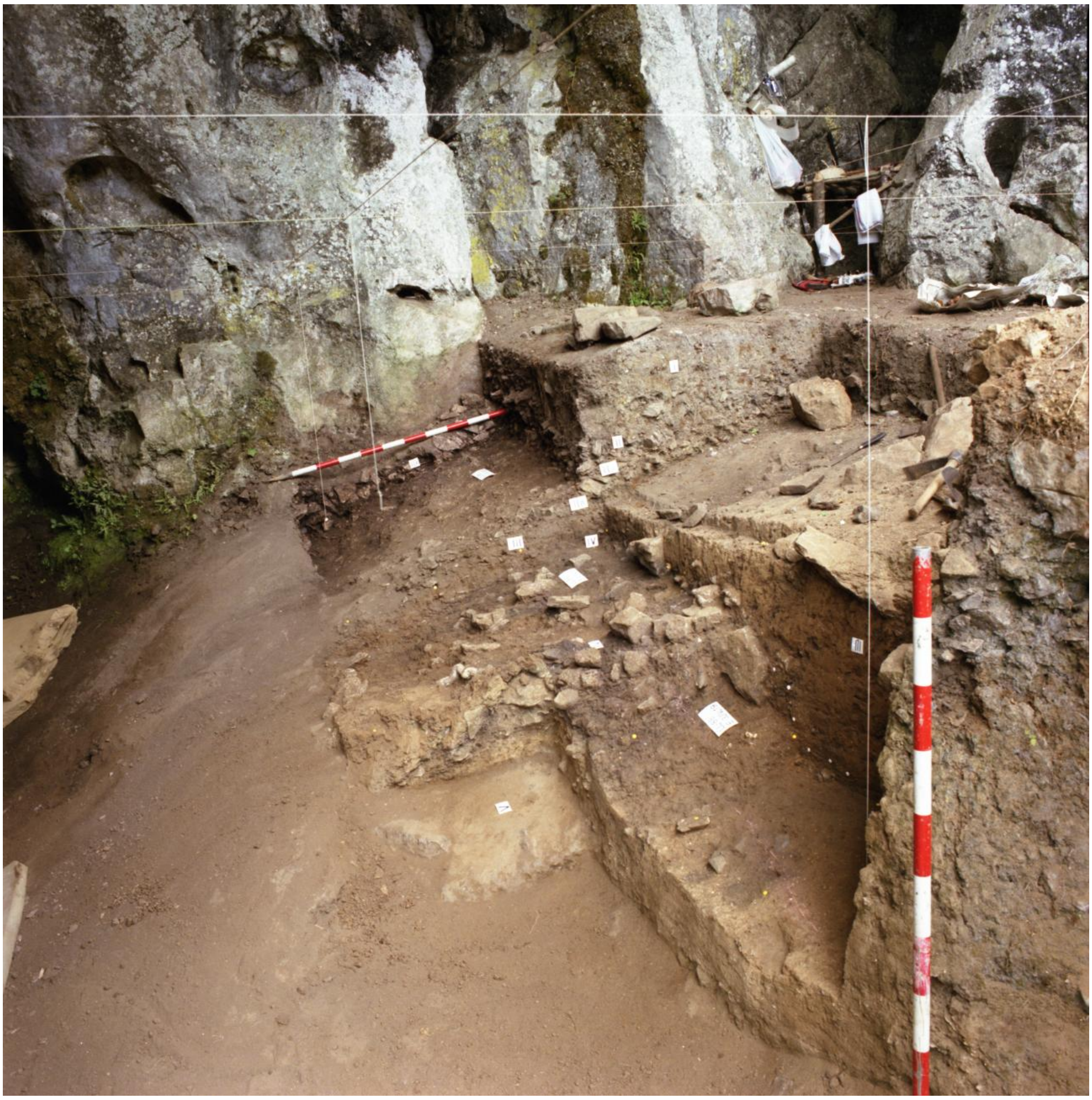

**S3 Fig.** General view of the vestibule (Vestíbulo). Photo: Javier Fortea.

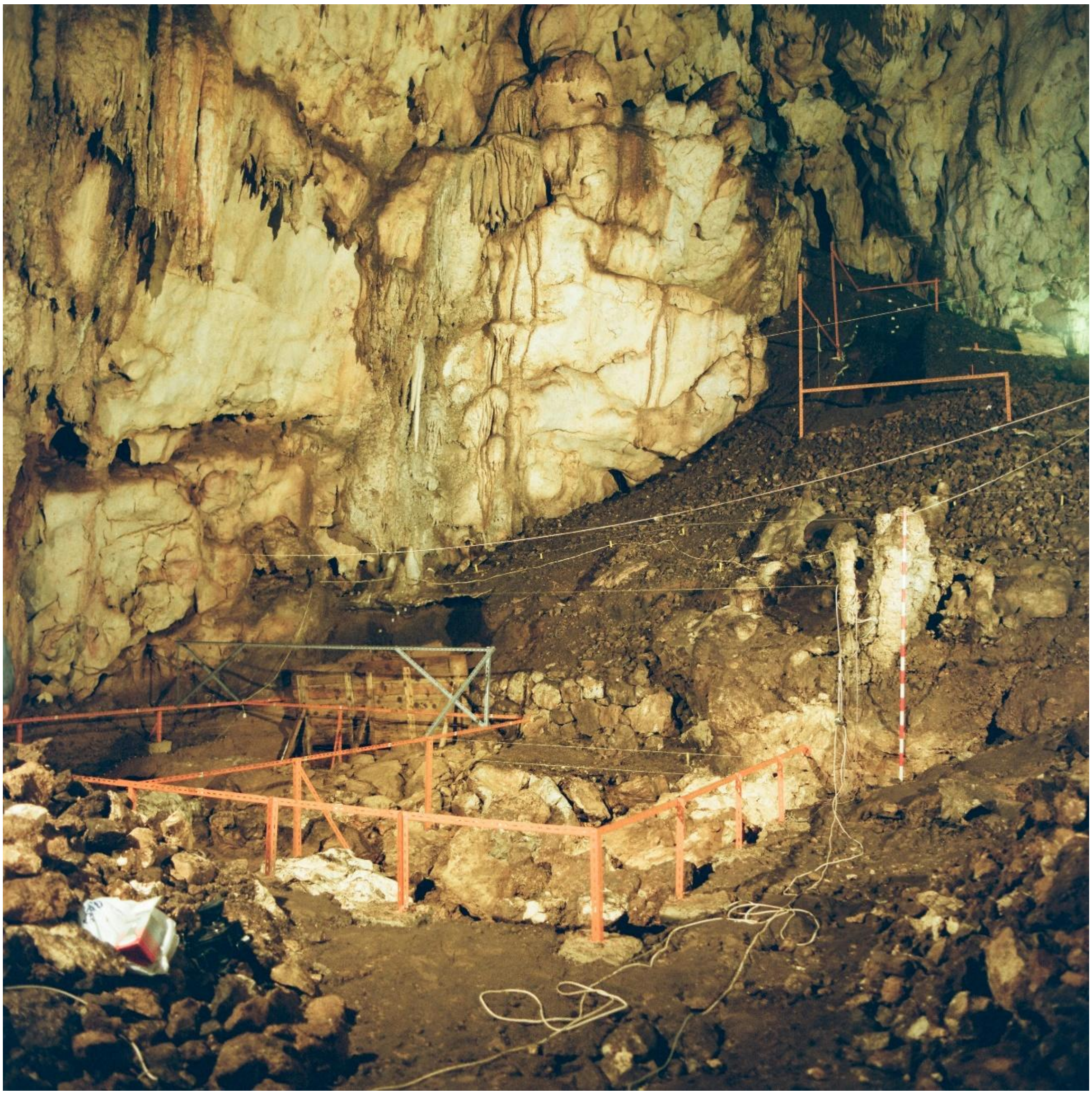

**S4 Fig.** View of the dejection cone, including the Cono Anterior (above) and Cono Posterior (below) excavation sectors. Photo: Javier Fortea.

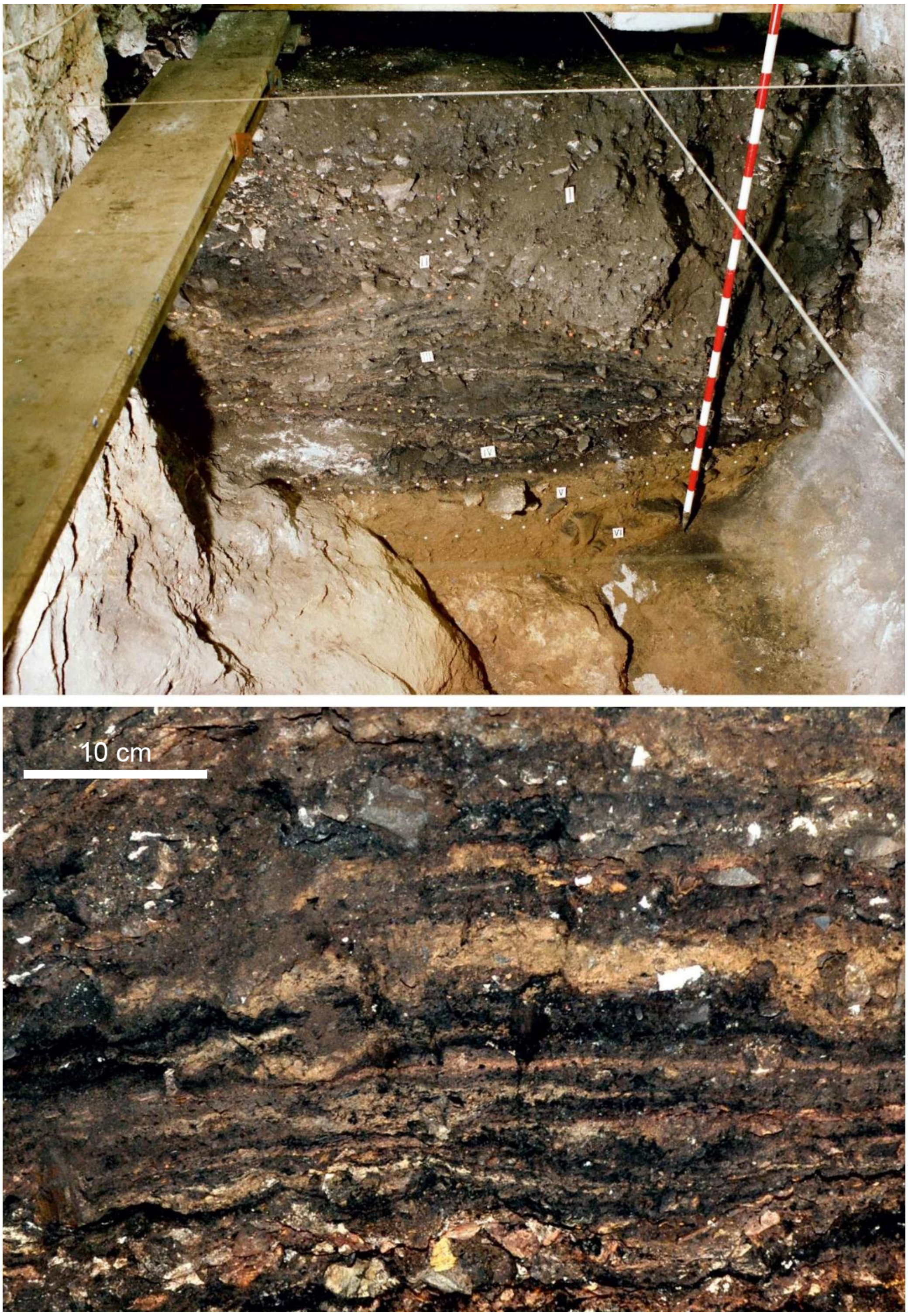


**S5 Fig.** General view of the stratigraphic section from Galería (above). Detail of Level III (below). Photos: Javier Fortea.

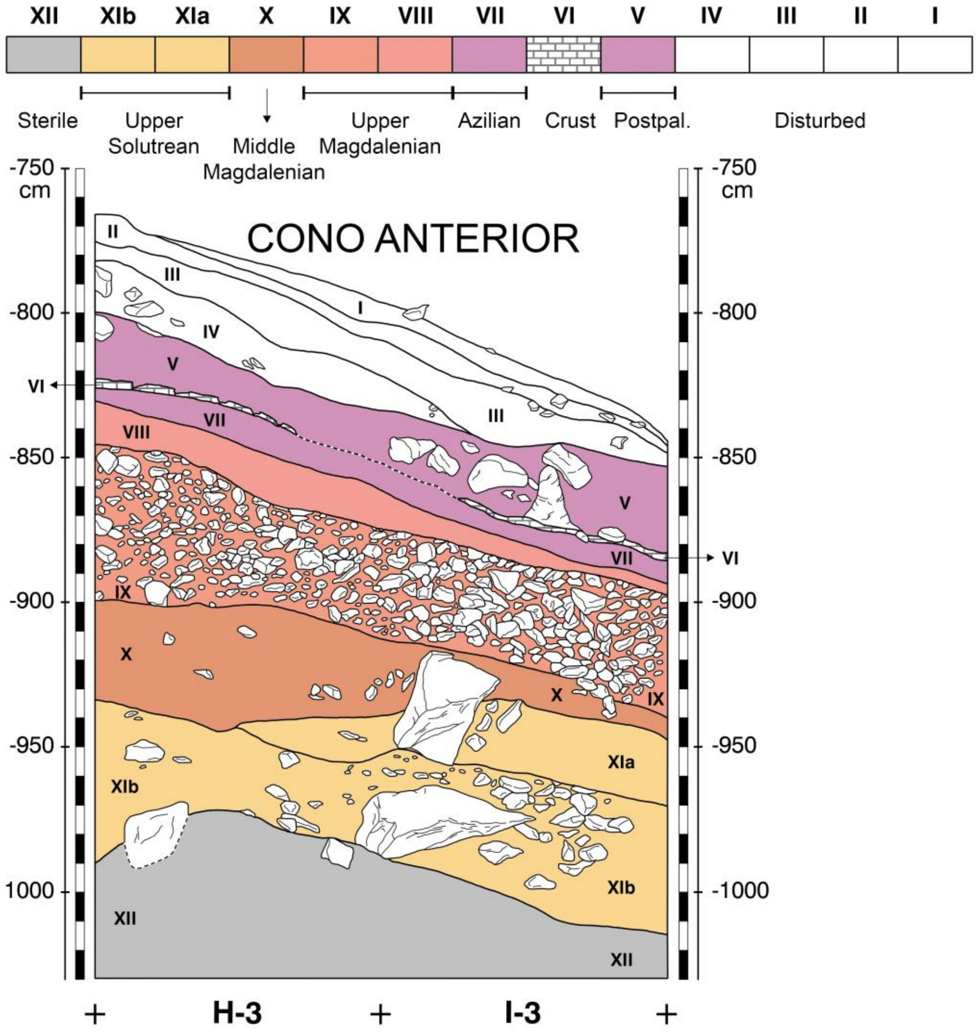
XII XIb XIa X IX VIII VII VI V IV III II I
Sterile
Upper Solutrean
Middle Magdalenian
Upper Magdalenian
Azilian
Crust
Postpal.
Disturbed
CONO ANTERIOR
-750 cm
-800
-850
-900
-950
1000
-1000
H-3
I-3

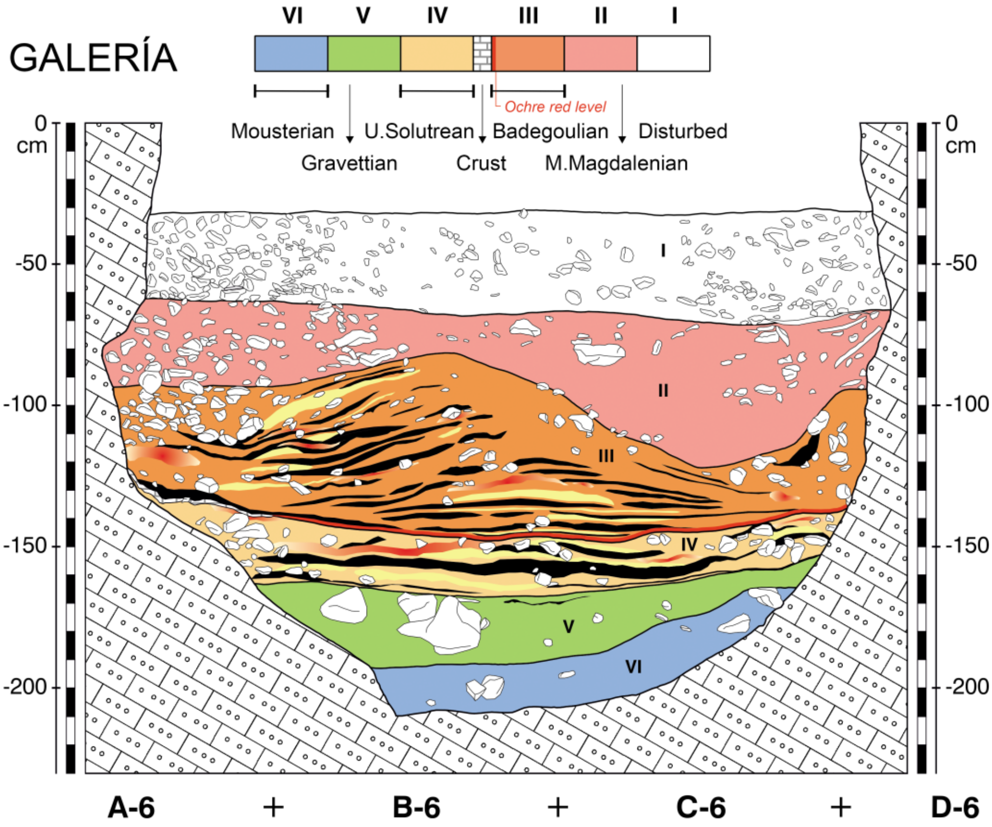
GALERÍA
VI V IV III II I
Mousterian
Gravettian
U.Solutrean
Crust
Ochre red level
Badegoulian
M.Magdalenian
Disturbed
0 cm
-50
-100
-150
-200
A-6
B-6
C-6
D-6

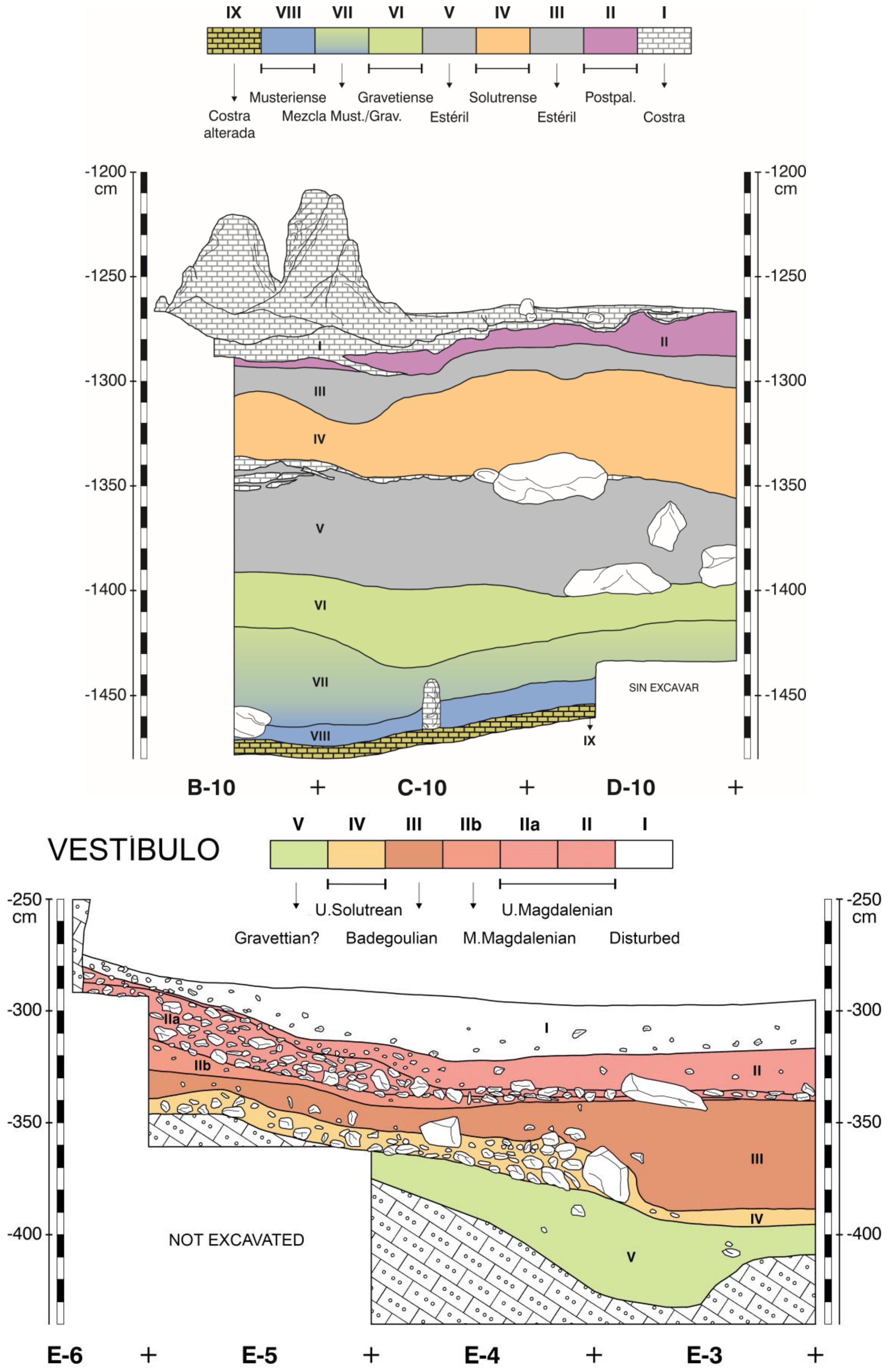


**S6 Fig.** Stratigraphic profiles of the four excavation sectors of Llonín cave. Photos: Elsa Duarte.

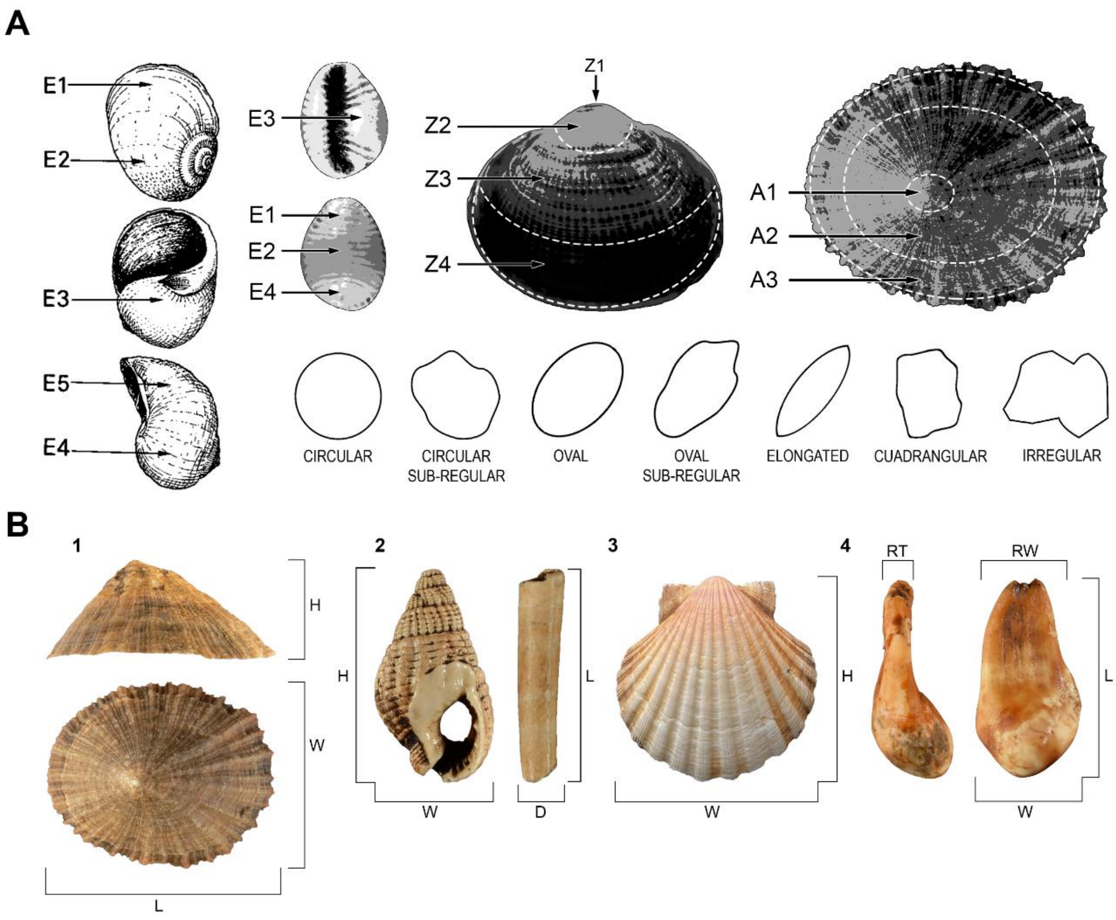


**S7 Fig.** Categories used to classify perforation location and morphology (A). Biometric criteria used to measure coiled and non-coiled gastropods, scaphopods, bivalves and red deer canines (B).

References:

Gutiérrez-Zugasti I. *La explotación de moluscos y otros recursos litorales en la región cantábrica durante el Pleistoceno final y el Holoceno inicial* [PhD thesis]. Santander: Universidad de Cantabria; 2009.

Taborin Y. Traces de façonnage et d'usage sur les coquillages perforés. In: Anderson PC, Beyries S, Otte M, Plisson H, editors. *Traces et fonction: les gestes retrouvés.* Vol. I. Liège: Service de Préhistoire; 1993. p. 255–267.

Tatá F, Cascalheira J, Marreiros J, Pereira T, Bicho N. Shell bead production in the Upper Paleolithic of Vale Boi (SW Portugal): an experimental perspective. *J Archaeol Sci.* 2014;42:29–41. https://doi.org/10.1016/j.jas.2013.10.029

Álvarez-Fernández E. *Los objetos de adorno-colgantes del Paleolítico superior y del Mesolítico en la Cornisa Cantábrica y en el Valle del Ebro: una visión europea* [PhD thesis]. Salamanca: Universidad de Salamanca; 2006.

HINDS

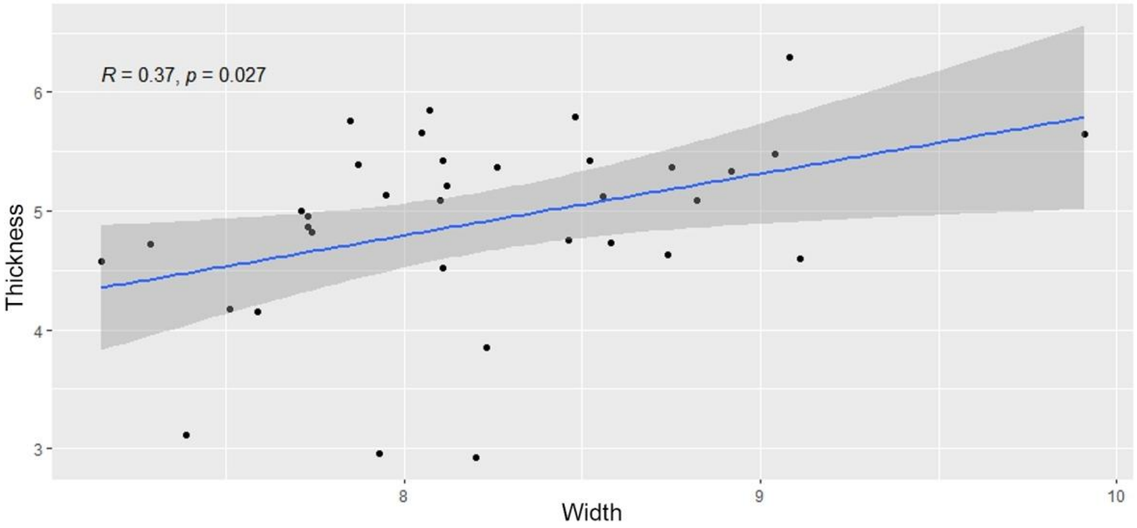


STAGS

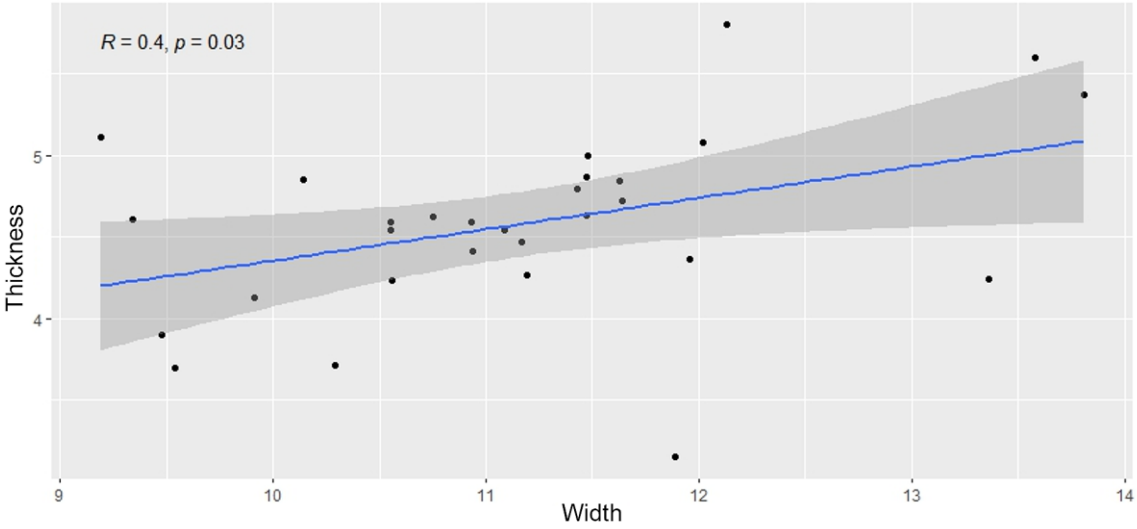


**S8 Fig.** Scatter plots showing the positive correlation between root width and root thickness among hind and stag canines from Llonín cave, proving its availability for sex estimation.
