# Supplementary material for: Identity and mobility through personal ornaments in Upper Paleolithic cantabrian hunter-gatherer societies: Insights from Llonín cave (Asturias, Spain)
Source: PLoS One. 2026 Jun 8;21(6):e0351170. doi: 10.1371/journal.pone.0351170 (PMC13245794; doi:10.1371/journal.pone.0351170)

S9 Fig. Images of the complete personal ornaments and raw materials assemblage of Llonín cave.

The acronyms employed for the identification of each piece have a tripartite code, with the different sections separated by dots (“.”) (example: CA.XI.100). The first section refers to the excavation sector (CA for Cono Anterior, CP for Cono Posterior, GA for Galería and VE for Vestíbulo), the second section refers to the stratigraphic level (UD stands for undetermined) and the third section is the identification number.

Trivia sp.

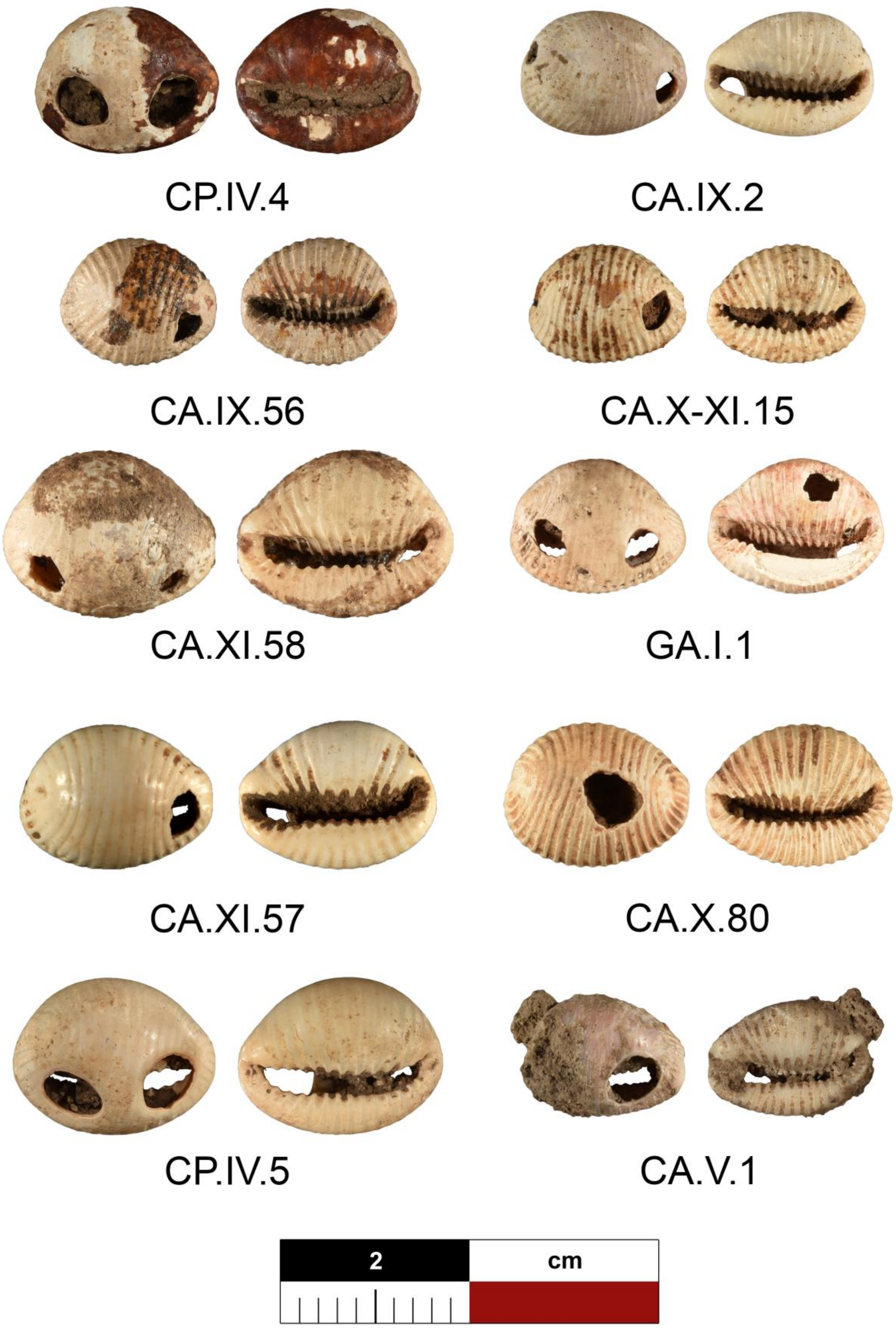
CP.IV.4
CA.IX.2
CA.IX.56
CA.X-XI.15
CA.XI.58
GA.I.1
CA.XI.57
CA.X.80
CP.IV.5
CA.V.1
2
cm

Turritellinella tricarinata

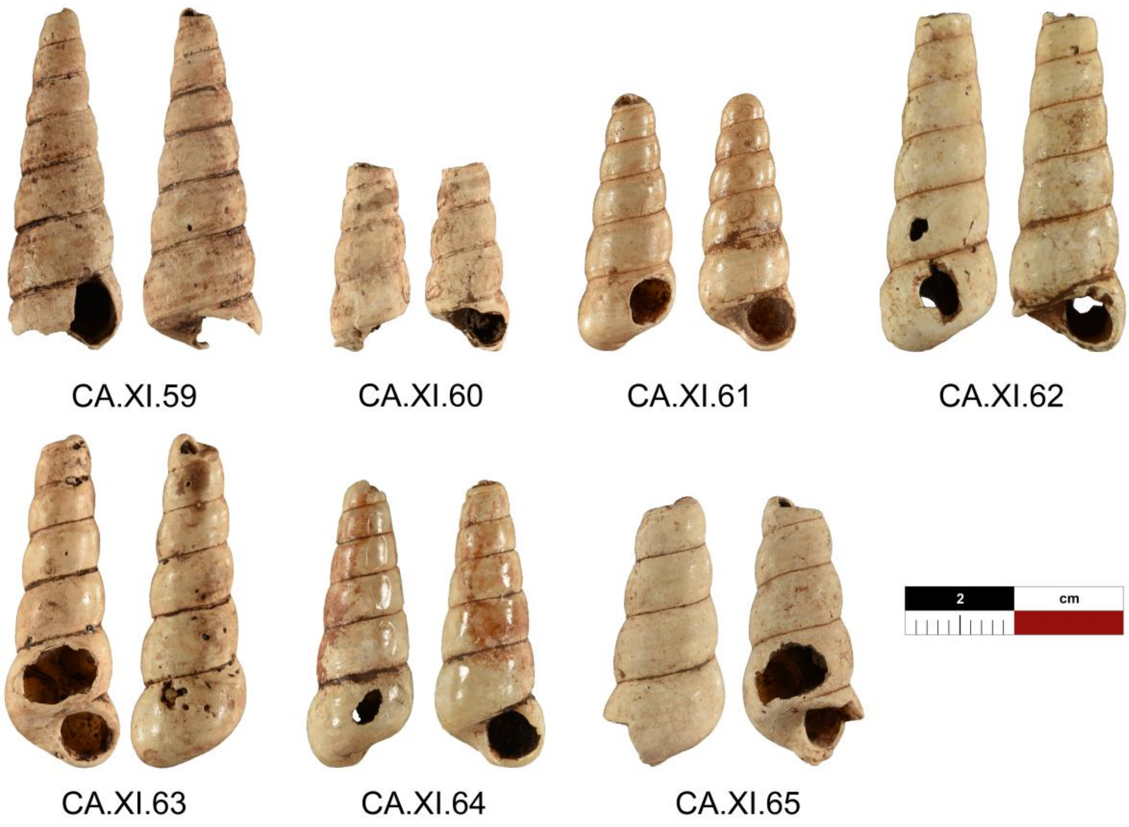

Tritia sp. (*T. reticulata* and *T. incrassata*)

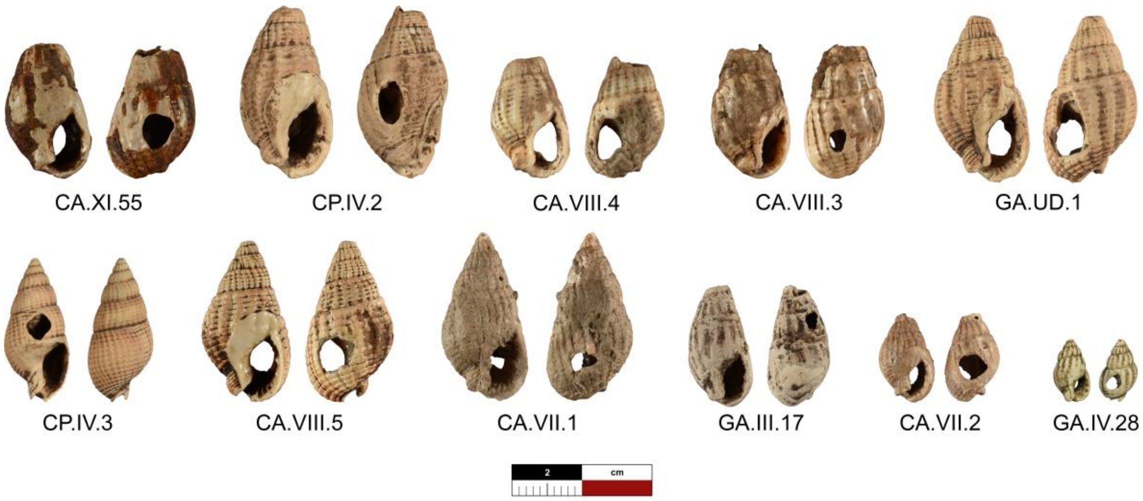

Tritia mutabilis

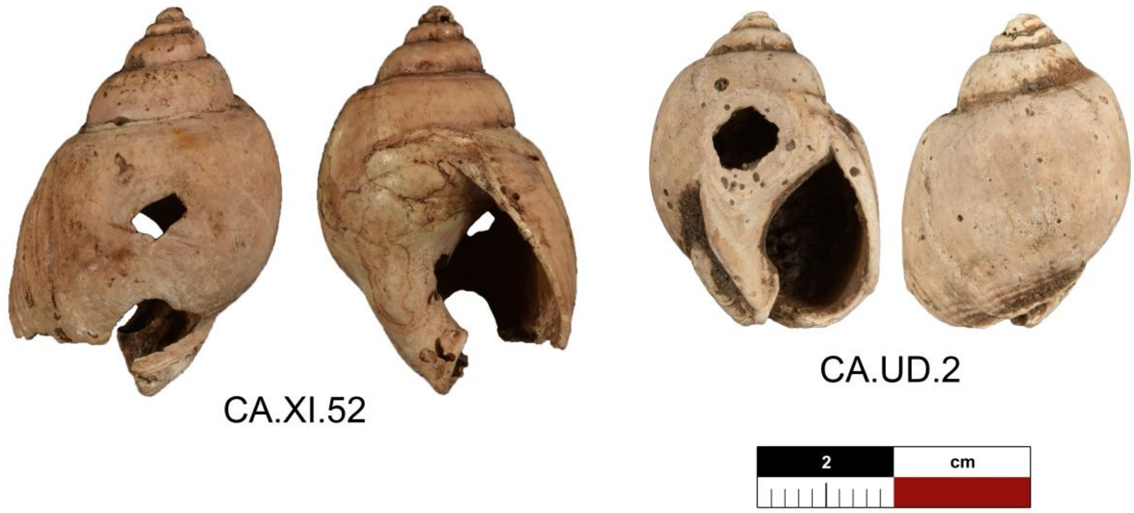

Nucella lapillus

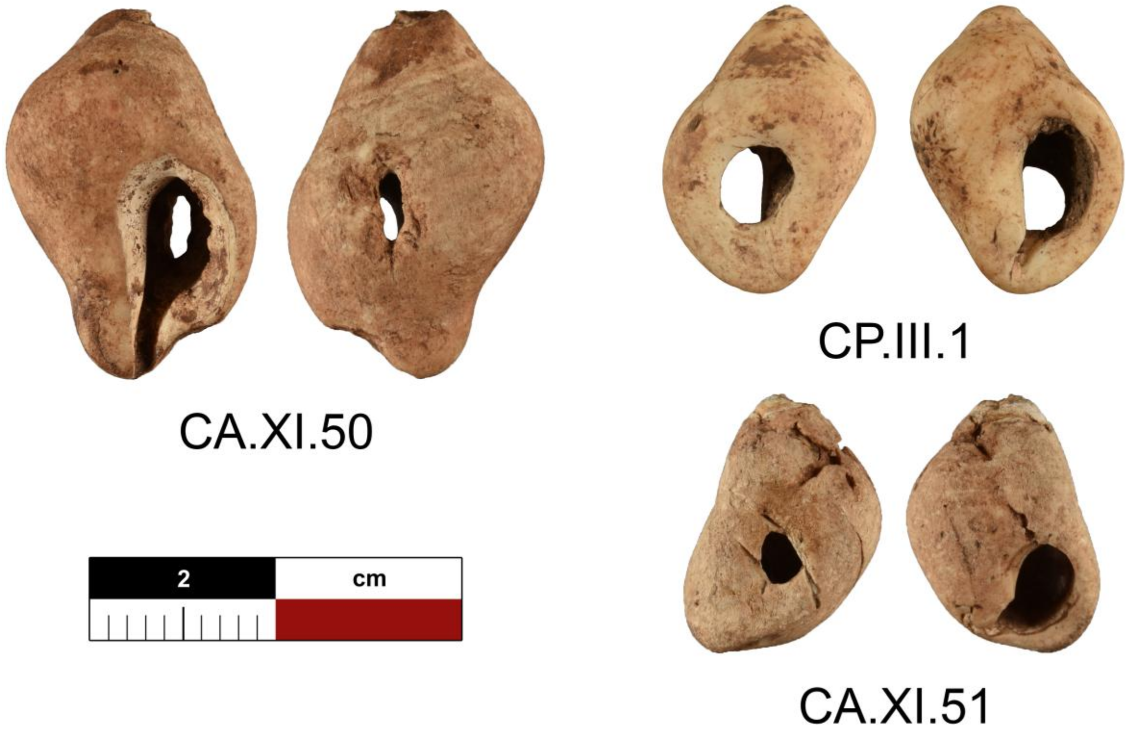

Salmo sp. vertebrae

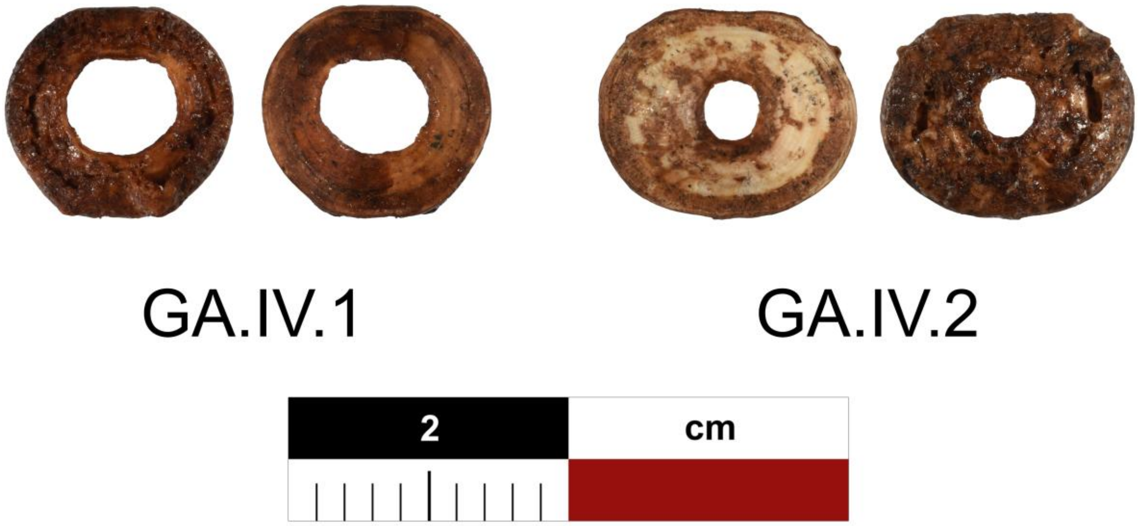

Solutrean *Littorina obtusata* and *Littorina saxatilis* (bottom row)

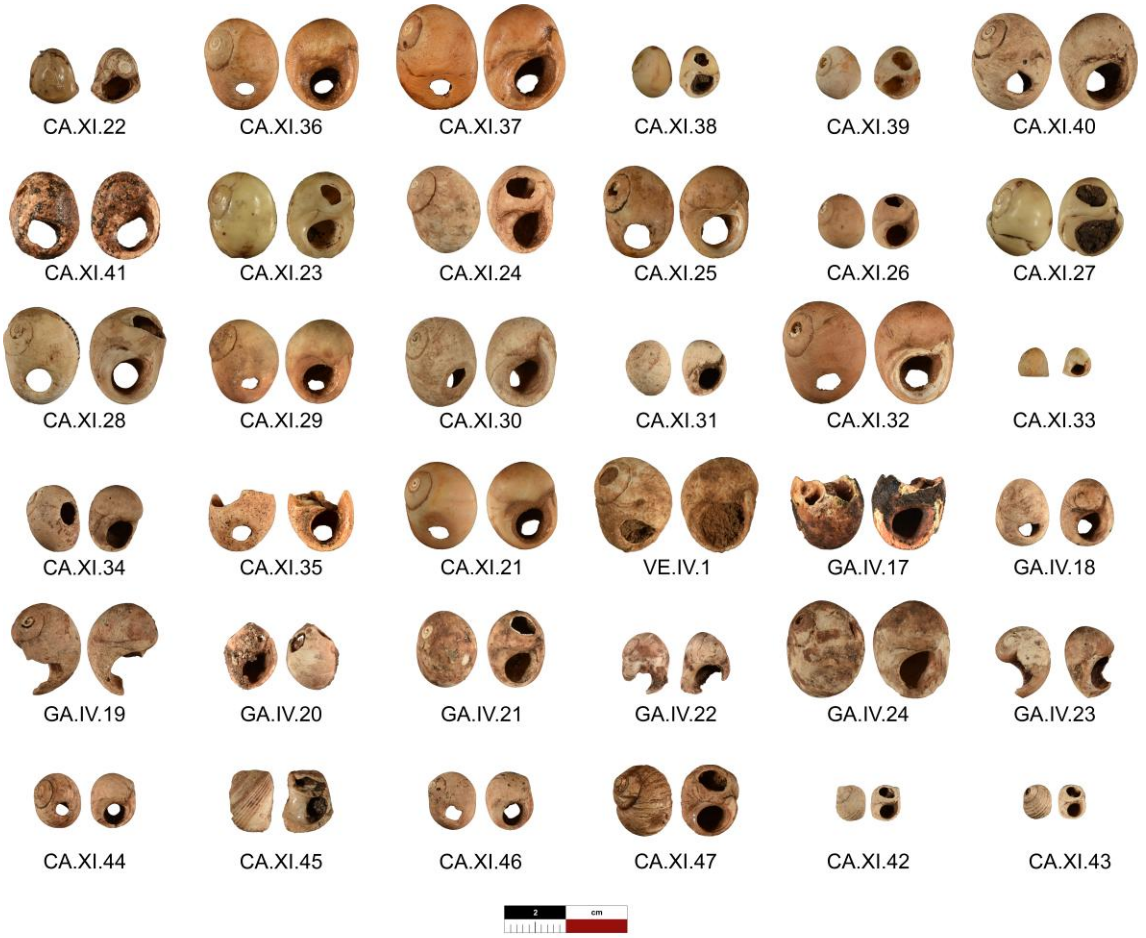

Magdalenian and Badegoulian *Littorina obtusata*

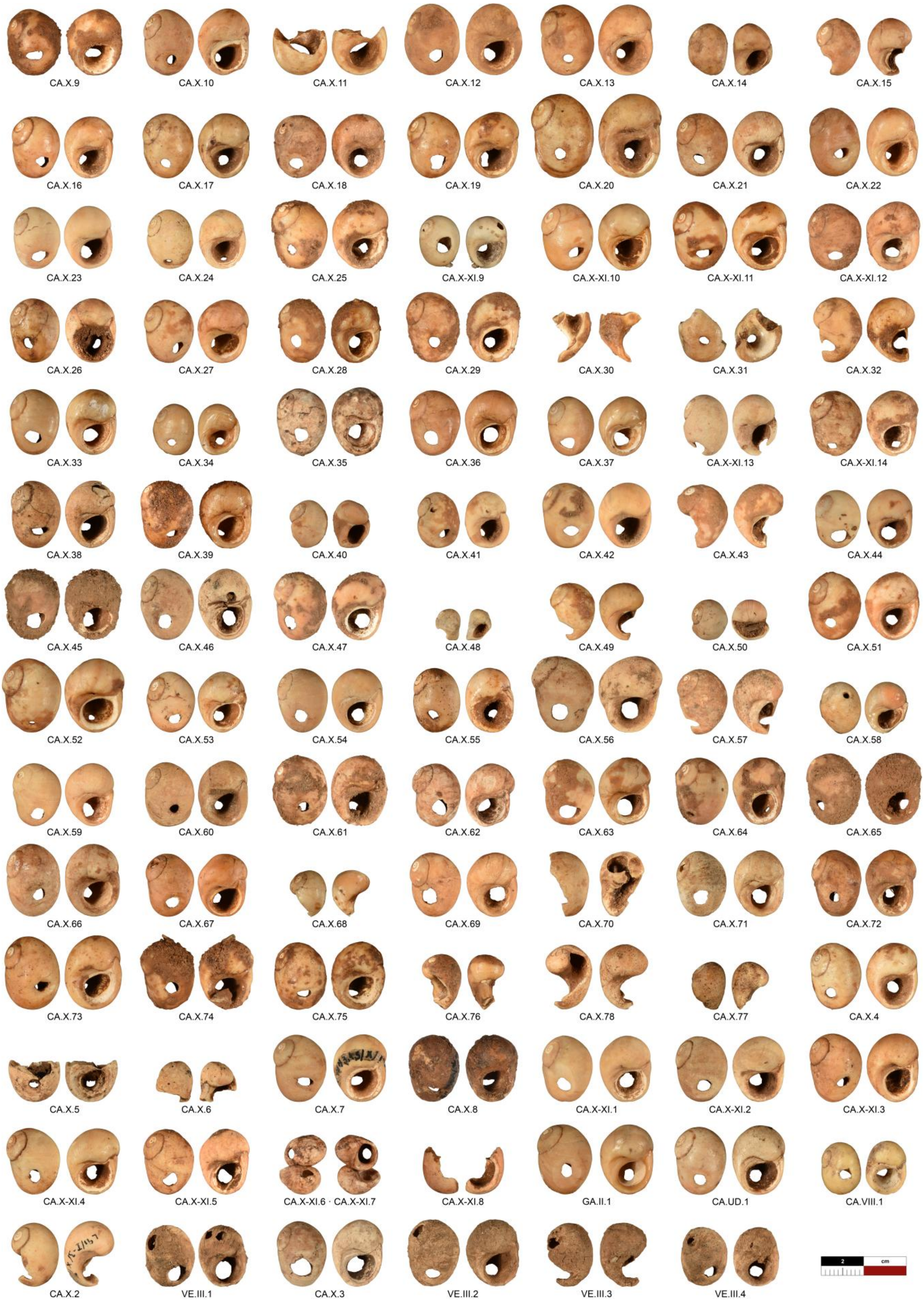

Patella sp. (*P. vulgata* and *P. depressa*)

CA.XI.53

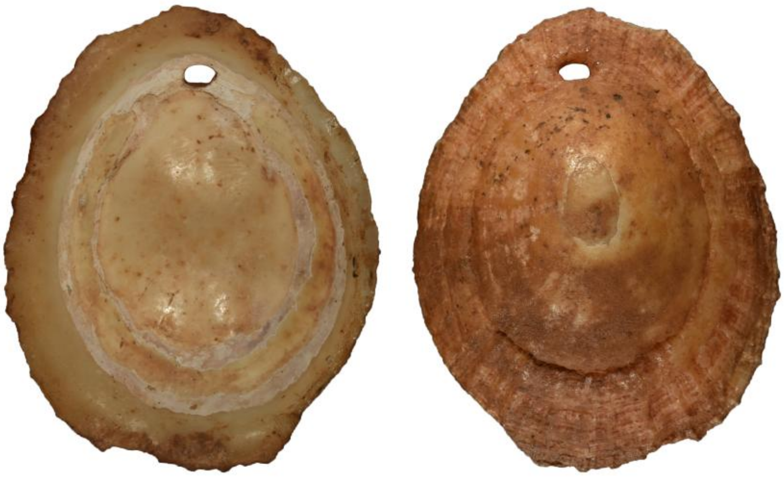

CA.IX.1

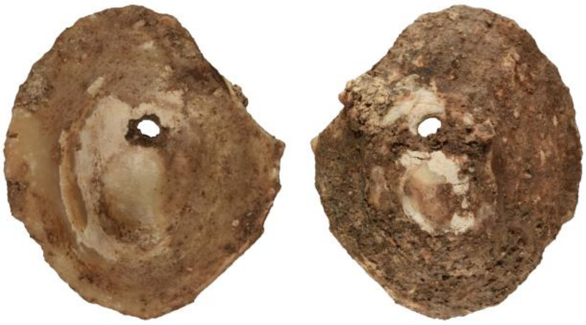

CA.VIII.2

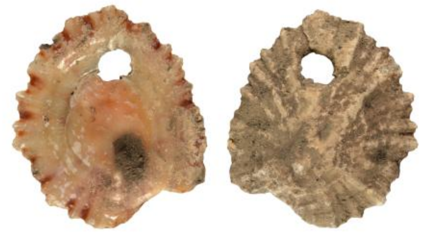

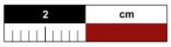

Antalis sp.

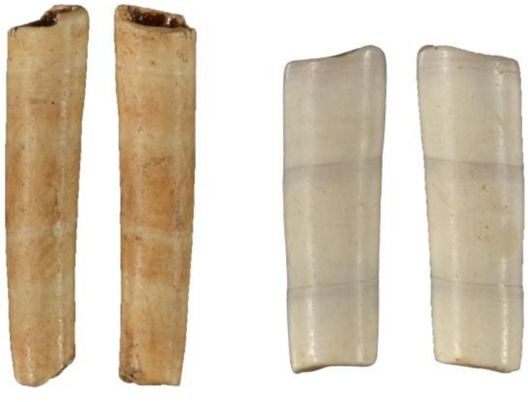

CA.XI.19

CA.XII.1

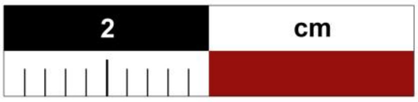

Bivalves (*Chlamys islandica*, *Glycymeris* sp. and *Veneridae*)

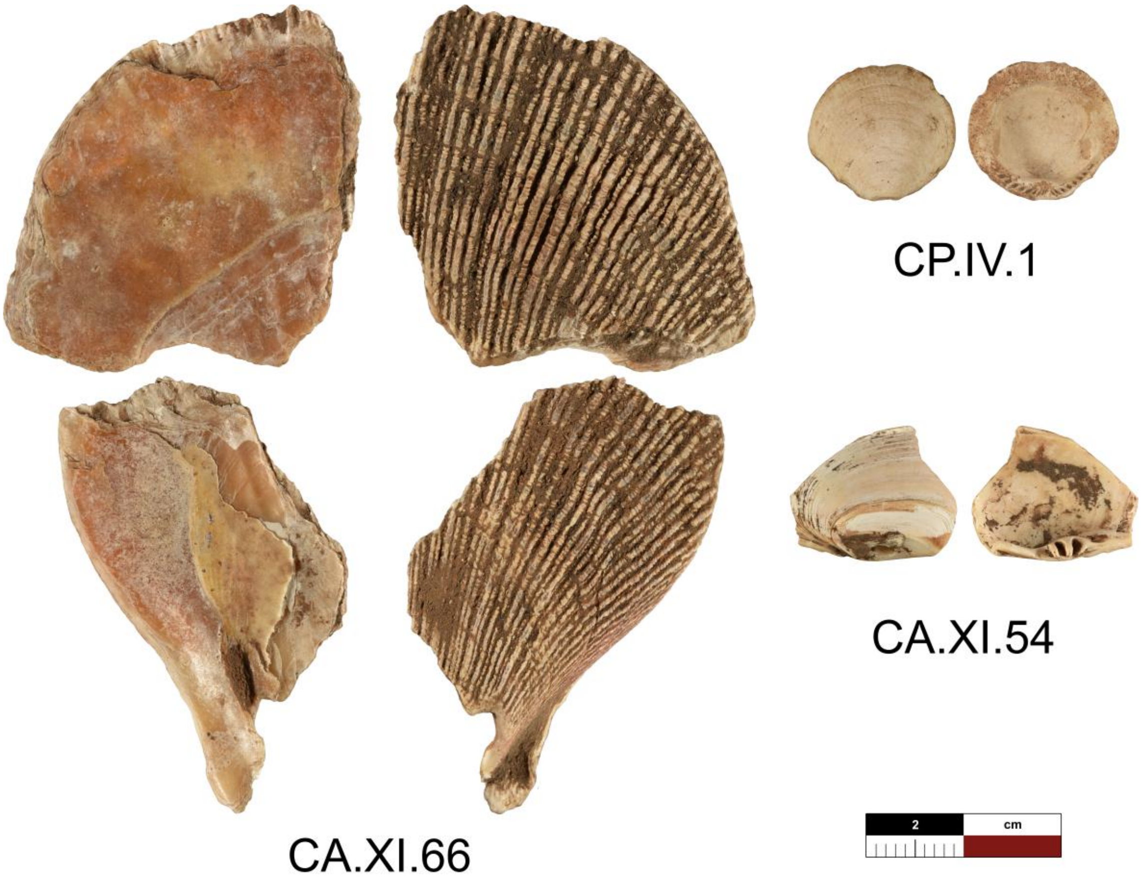

Rotularia sp. fossil bead

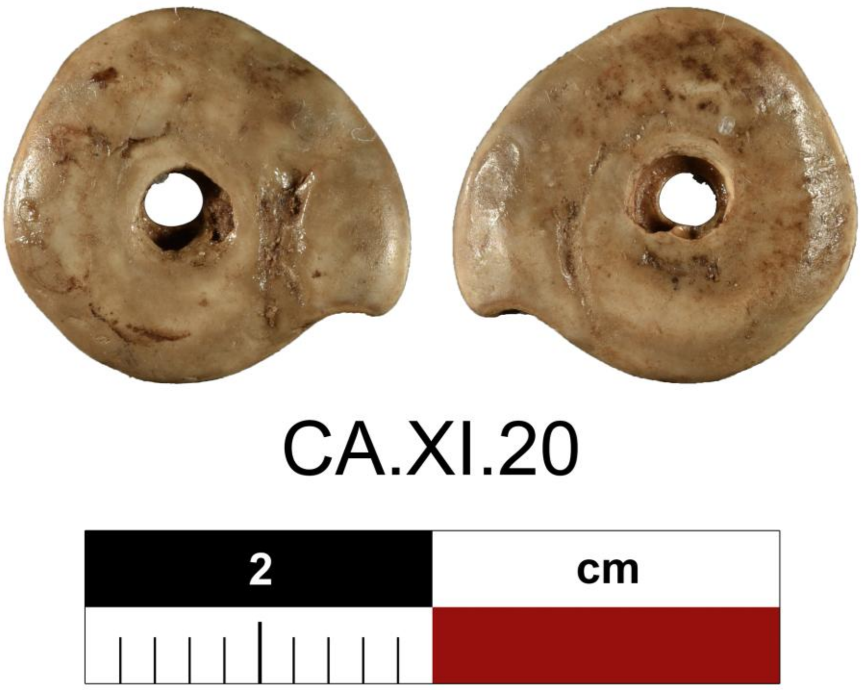

Upper Solutrean unperforated *Cervus elaphus* canines

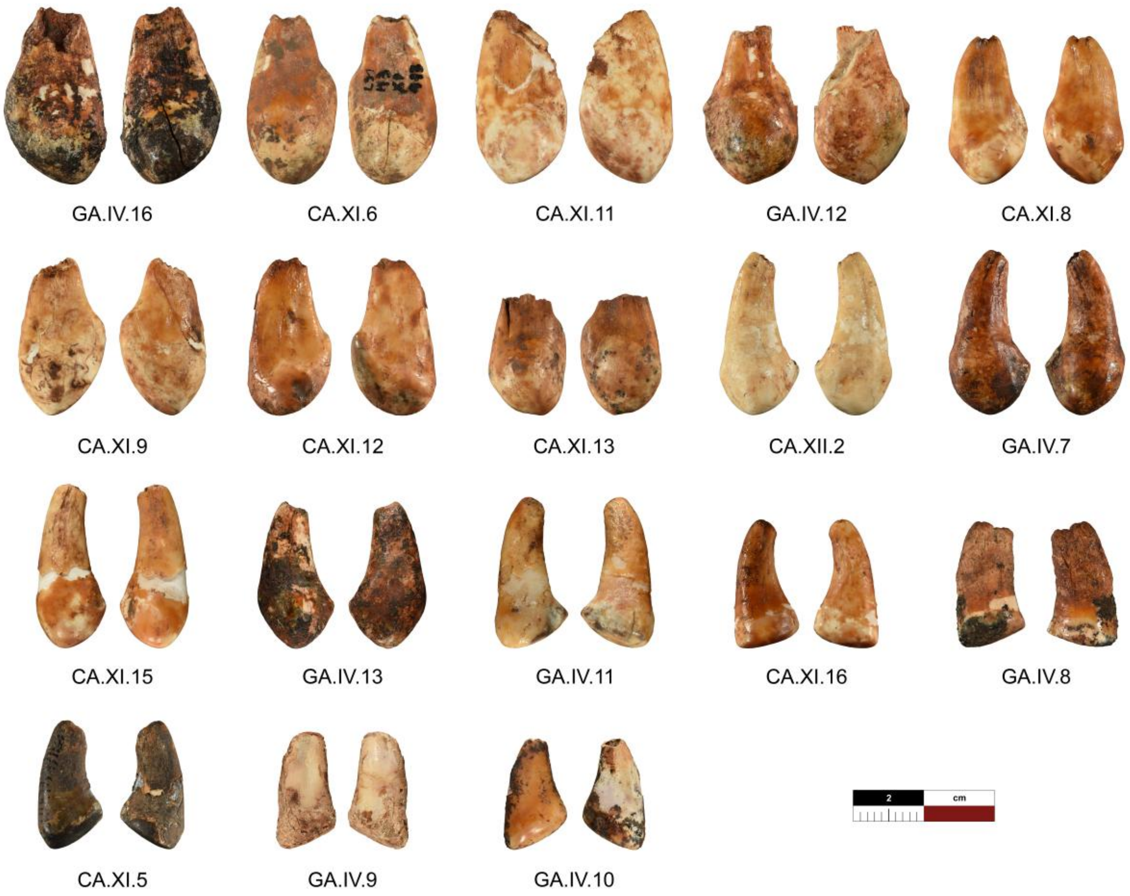

Magdalenian unperforated *Cervus elaphus* canines

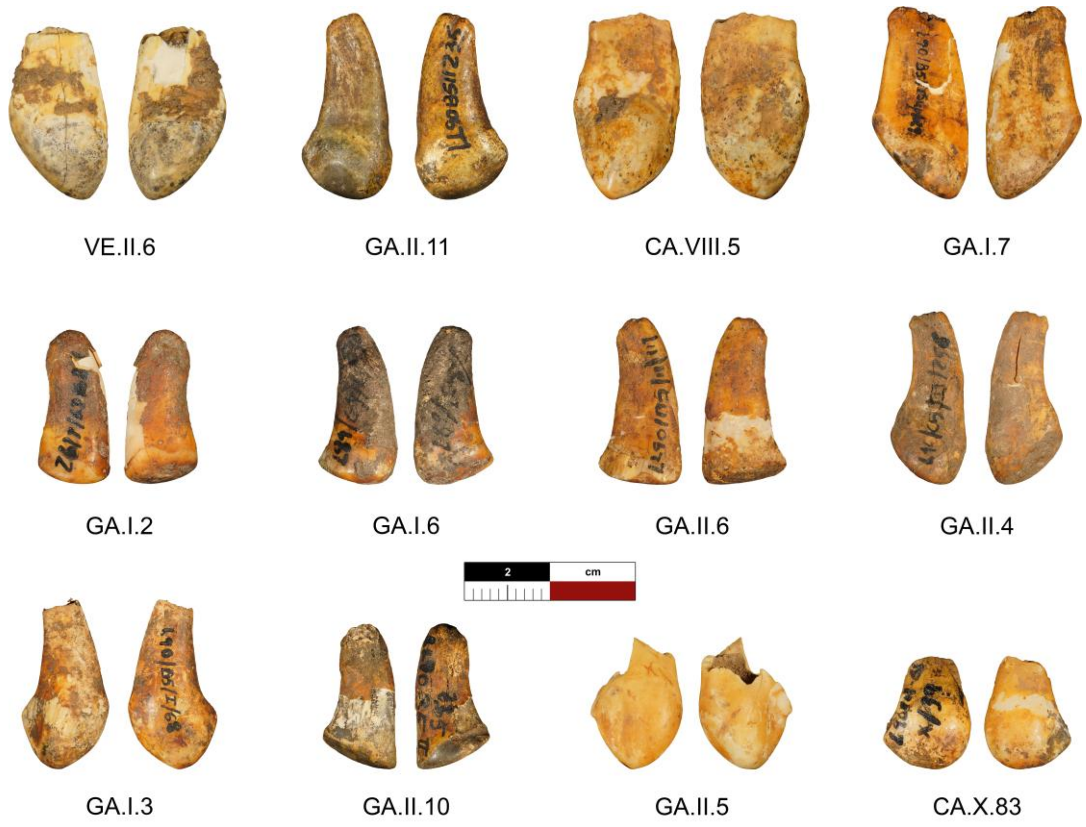

Badegoulian *Cervus elaphus* canines and *Bos/Bison* sp. incisors

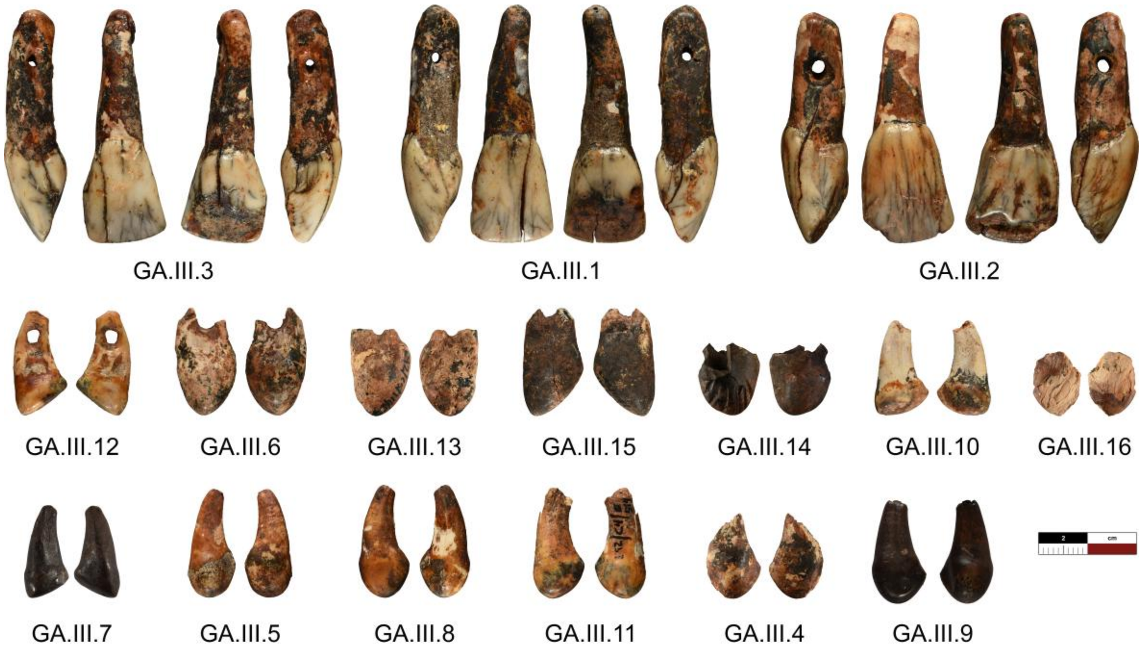

Middle Magdalenian *Canis lupus* canine

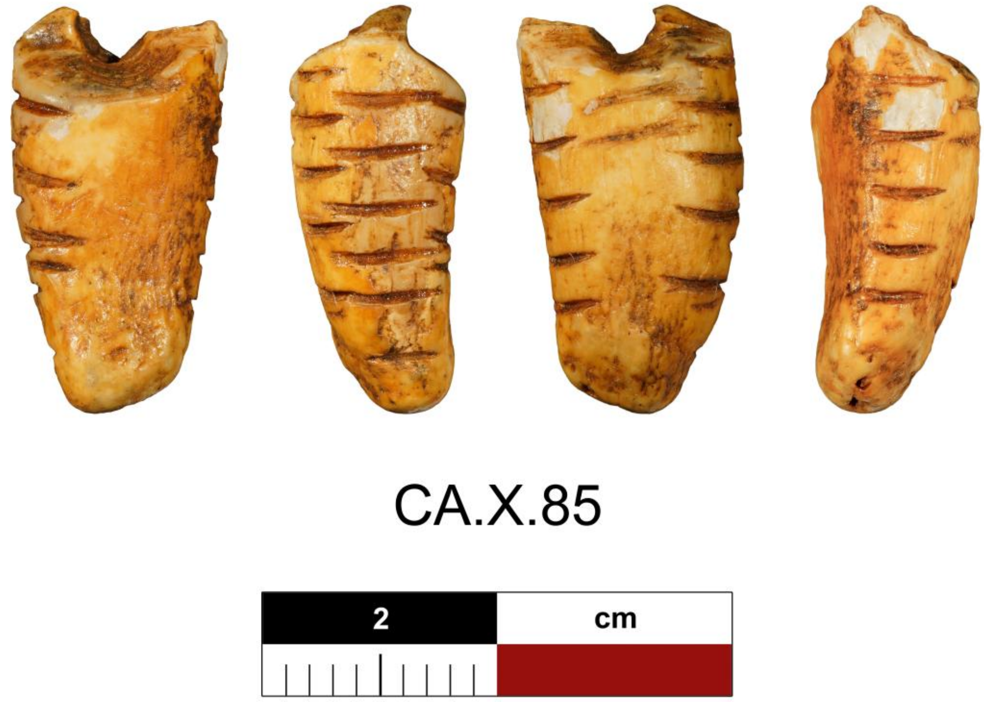

Upper Solutrean *Cervus elaphus* perforated canines, *Bos/Bison* sp. incisor, *Vulpes* sp. canine and *Capra pyrenaica* incisors.

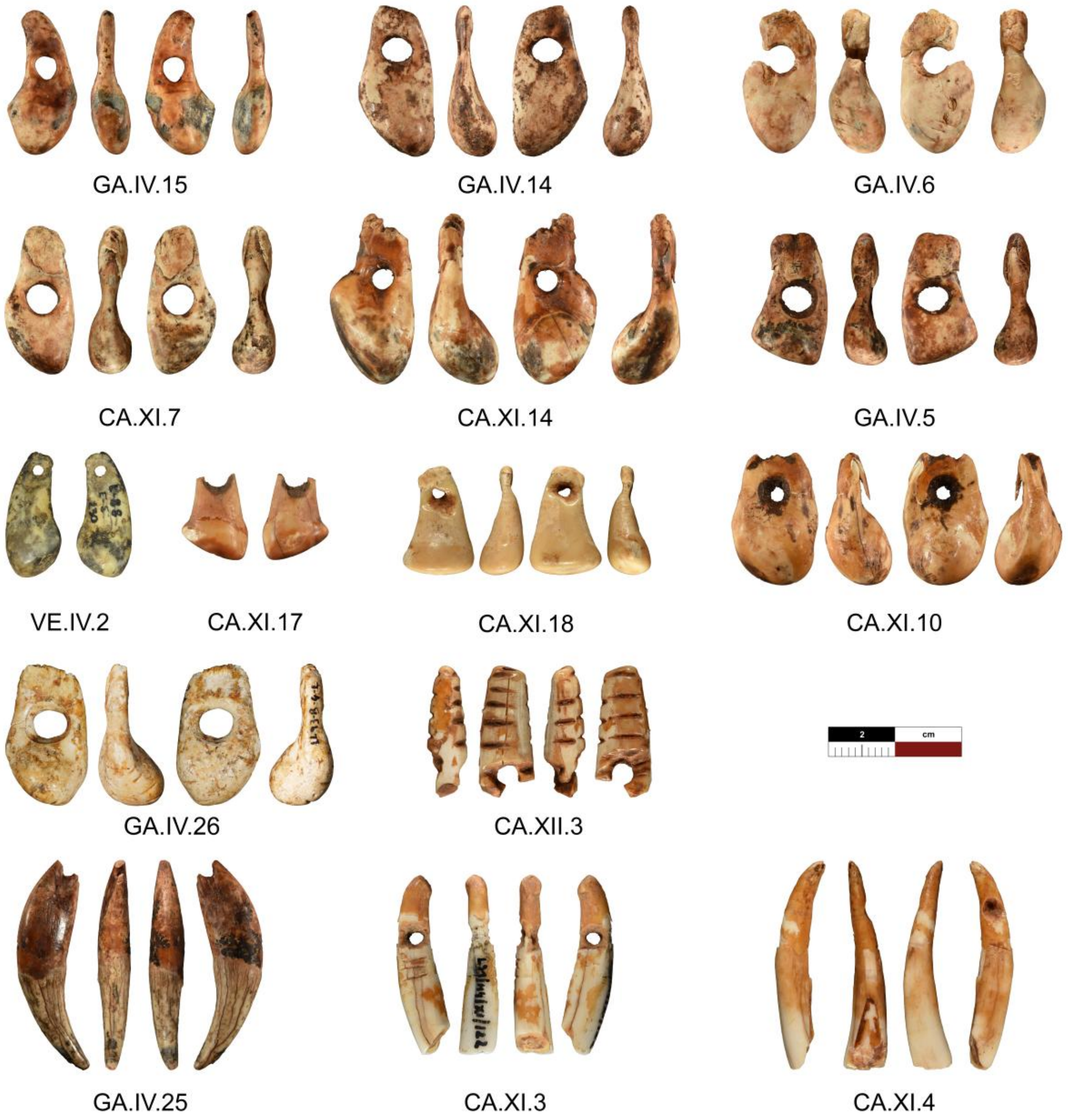

Magdalenian and Azilian (CA.VII.3) perforated *Cervus elaphus* canines

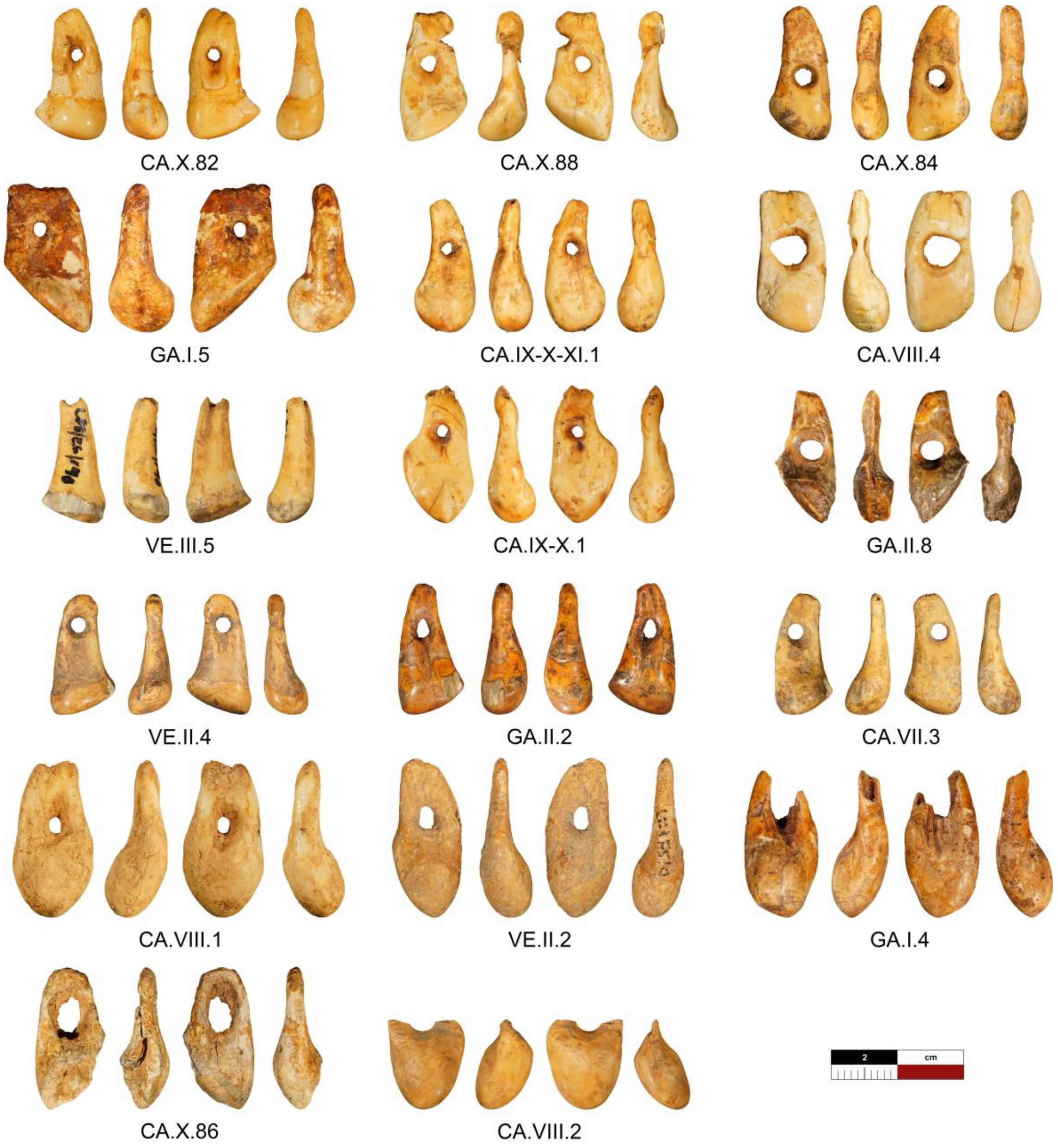

Upper Solutrean pierced *Cervus elaphus* scapula

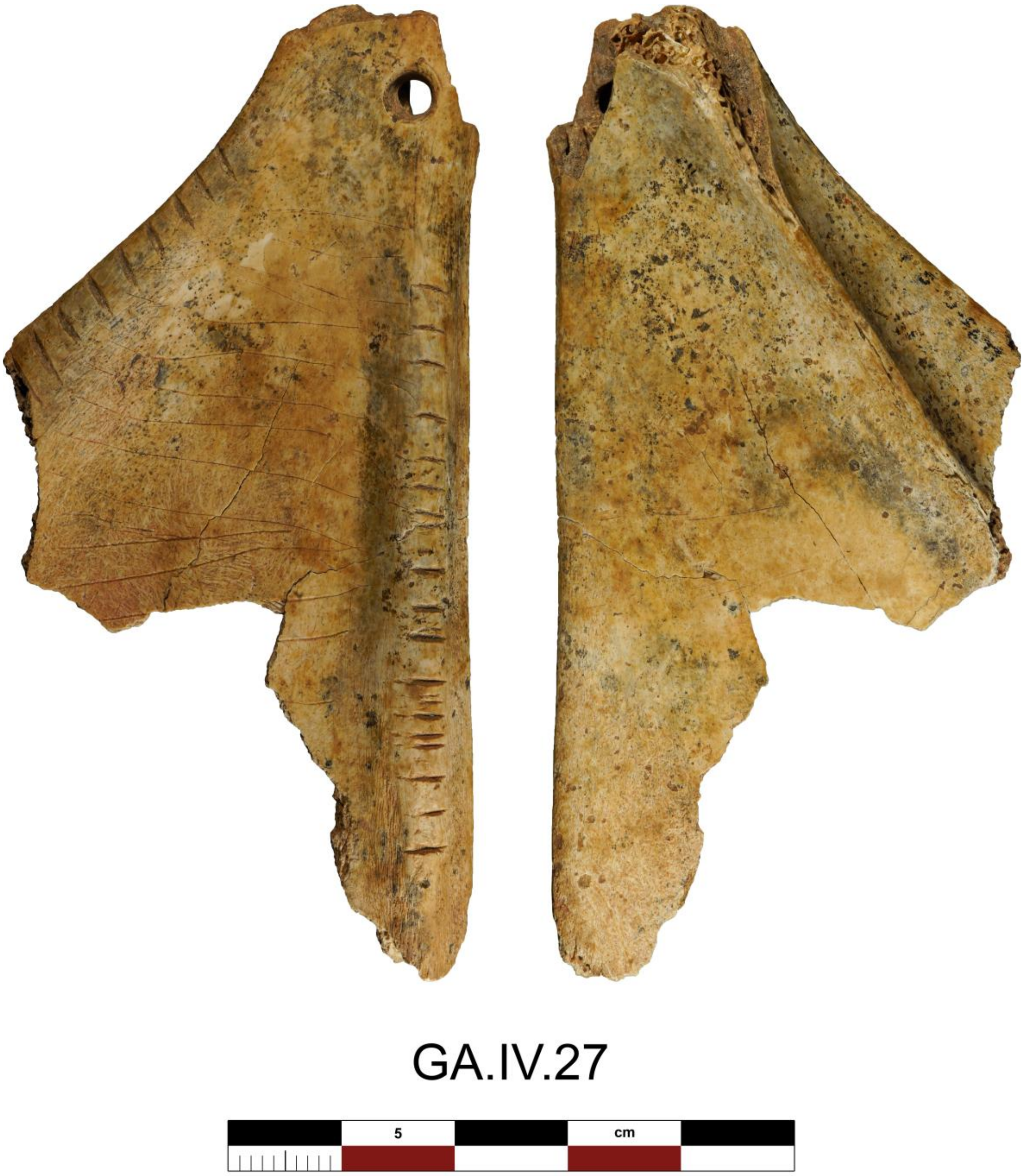

GA.IV.27

Upper Solutrean (top) and Middle Magdalenian (bottom) perforated *Equus ferus* hyoid bones

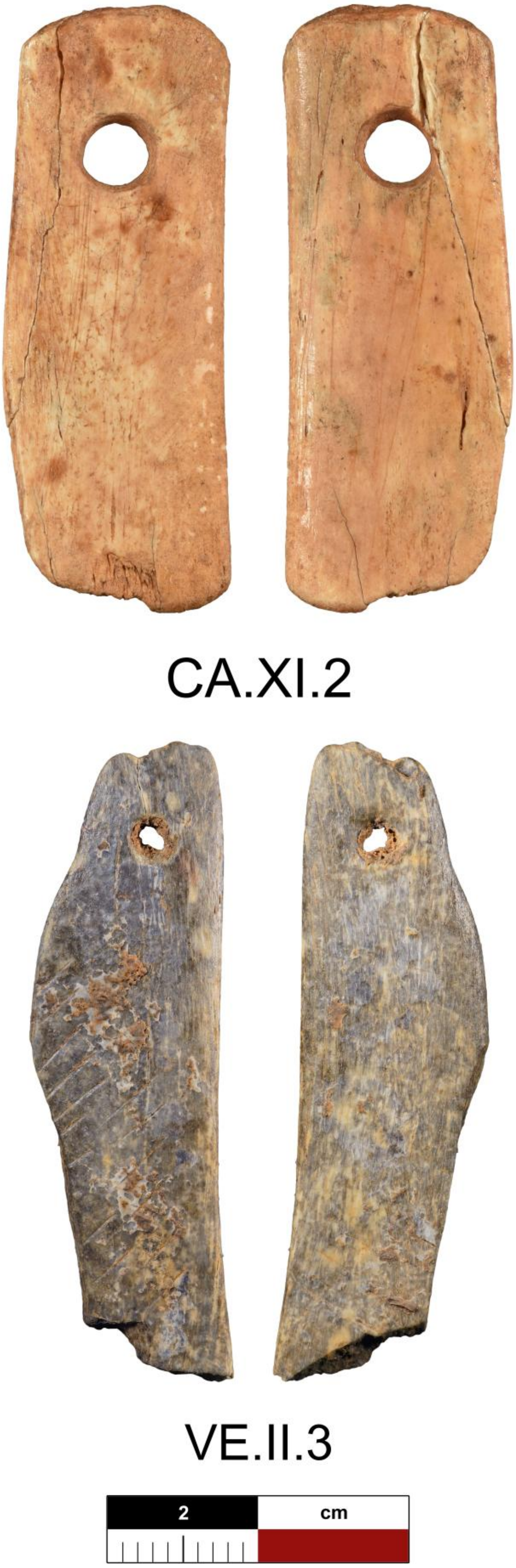

Middle Magdalenian bone disk

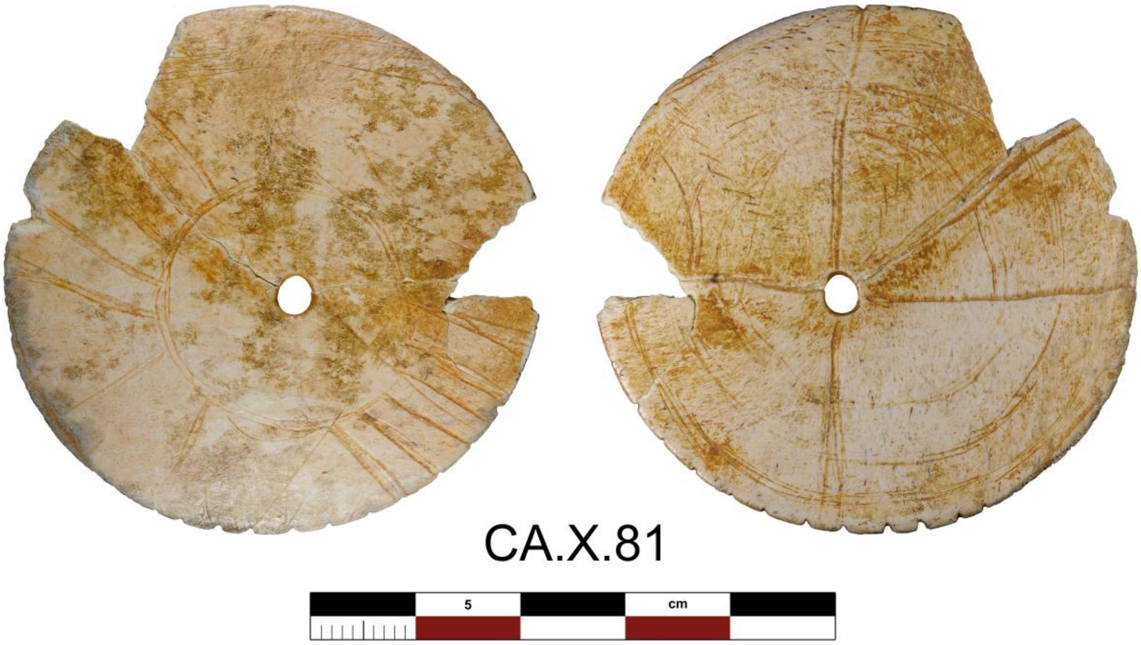

Capra pyrenaica premolar, pierced *Littorina littorea*, *Littorina* sp. fragments, *mammalia* pierced bone fragment, modified *pisces* vertebra

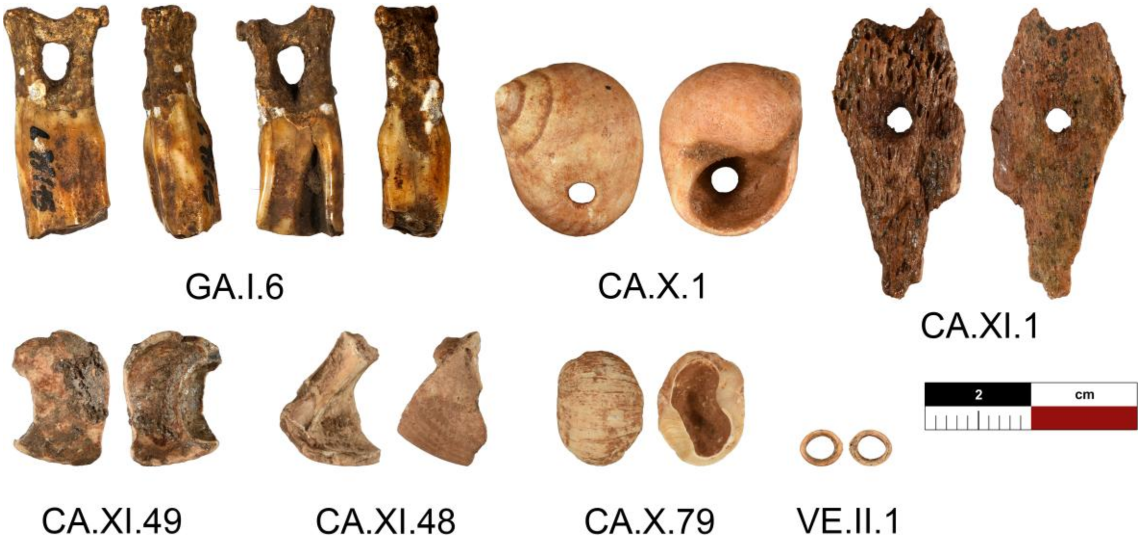

Supplement: S9 Fig — (PDF) [file pone.0351170.s007.pdf]
