# Supplementary material for: Identity and mobility through personal ornaments in Upper Paleolithic cantabrian hunter-gatherer societies: Insights from Llonín cave (Asturias, Spain)
Source: PLoS One. 2026 Jun 8;21(6):e0351170. doi: 10.1371/journal.pone.0351170 (PMC13245794; doi:10.1371/journal.pone.0351170)

S10 Fig. Graphic representation of use-wear and ochre distribution on personal ornaments from Llonín cave

The following illustrations present schematic representations of the location of use-wear traces and ochre staining on personal ornaments from Llonín Cave. Only those specimens with precise chrono-stratigraphic attribution and exhibiting clear or ambiguous use-wear traces have been included. Two shades of blue have been used to depict use-wear traces, with the darker tone indicating areas where such traces are more intensively developed. Regarding ochre, its representation has been omitted when it occurs as microscopic spots or when it is present on the inner surfaces of mollusk shells, which are difficult to render schematically. Arrows indicate areas of the ornaments where use has produced noticeable deformation or notching on perforations or on the surface of the pieces. The acronyms follow the same tripartite code explained for S9 Fig. and refer to the same specimens where applicable. The last figure shows the suspension modes inferable from the different distribution of use-wear traces on the specimens.

Littorina obtusata

Upper Solutrean

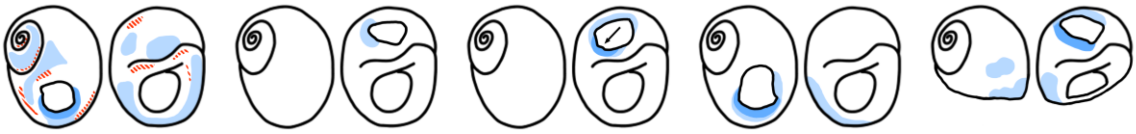

CA.XI.21 CA.XI.23 CA.XI.24 CA.XI.25 CA.XI.26

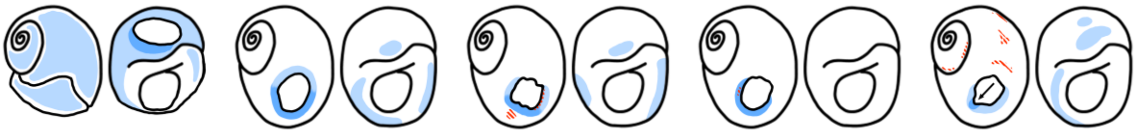

CA.XI.27 CA.XI.28 CA.XI.29 CA.XI.30 CA.XI.32

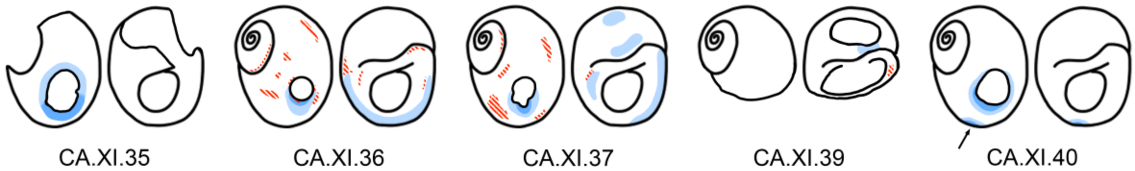

CA.XI.35 CA.XI.36 CA.XI.37 CA.XI.39 CA.XI.40

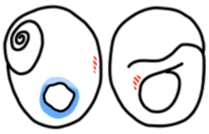

GA.IV.18

Badegoulian

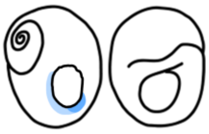

VE.III.1

Middle Magdalenian

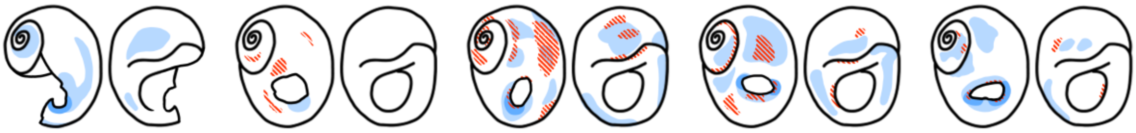

CA.X.2 CA.X.3 CA.X.4 CA.X.7 CA.X.9

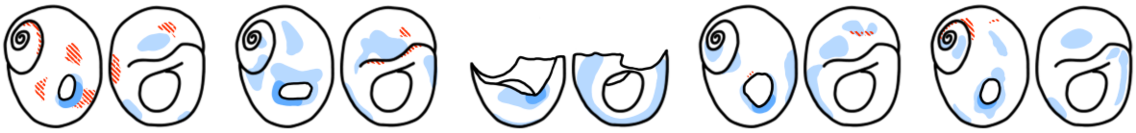

CA.X.10 CA.X.75 CA.X.11 CA.X.12 CA.X.13

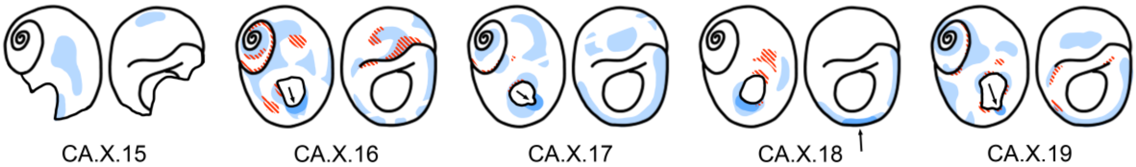

CA.X.15 CA.X.16 CA.X.17 CA.X.18 CA.X.19

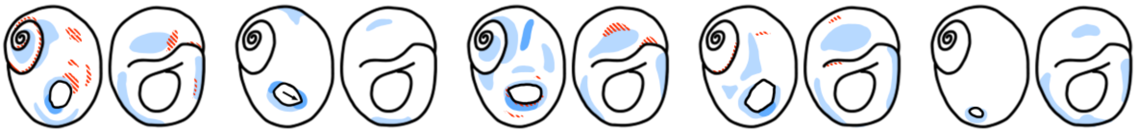

CA.X.20 CA.X.21 CA.X.22 CA.X.23 CA.X.24

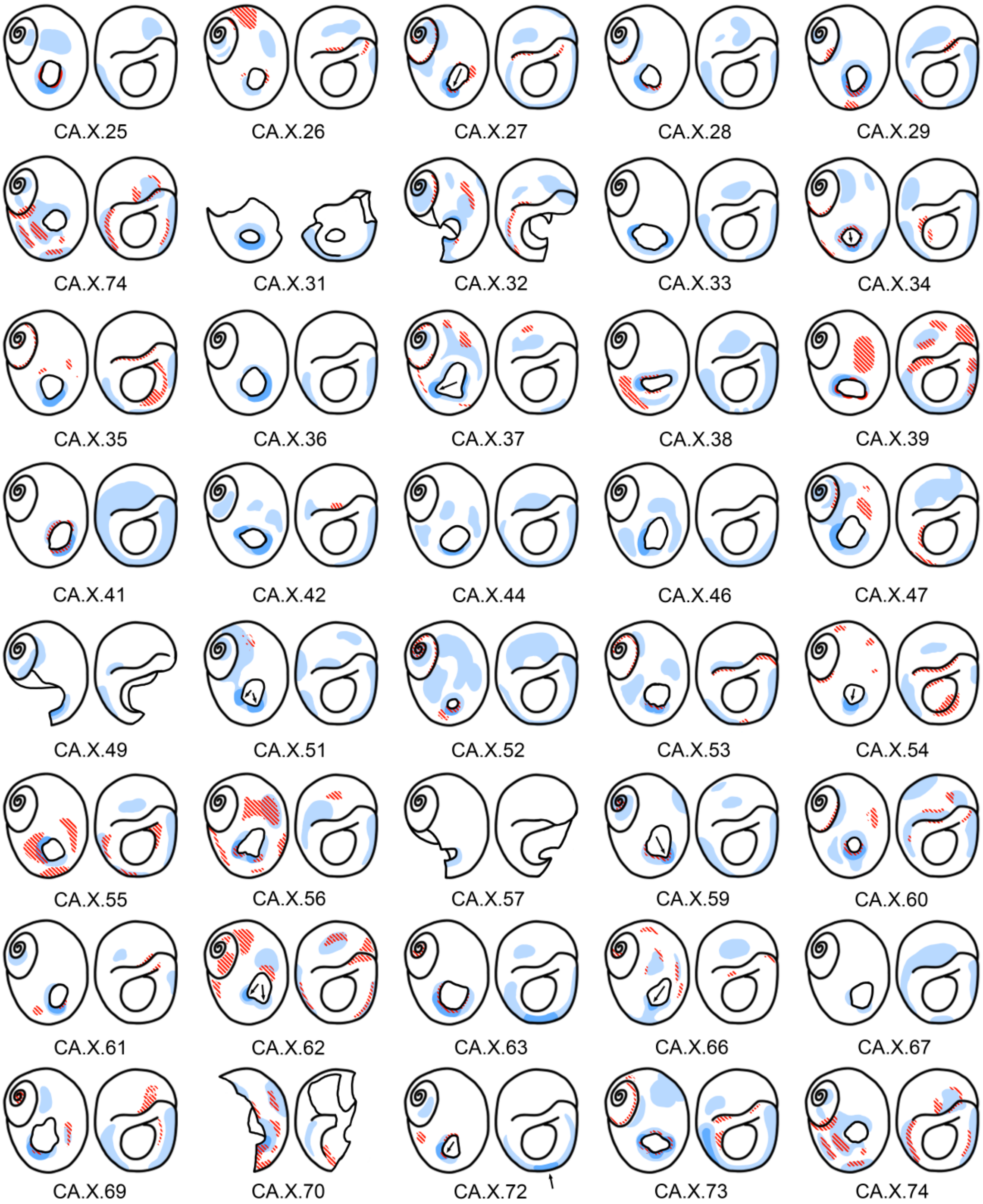

Upper Magdalenian

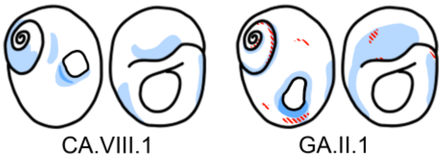

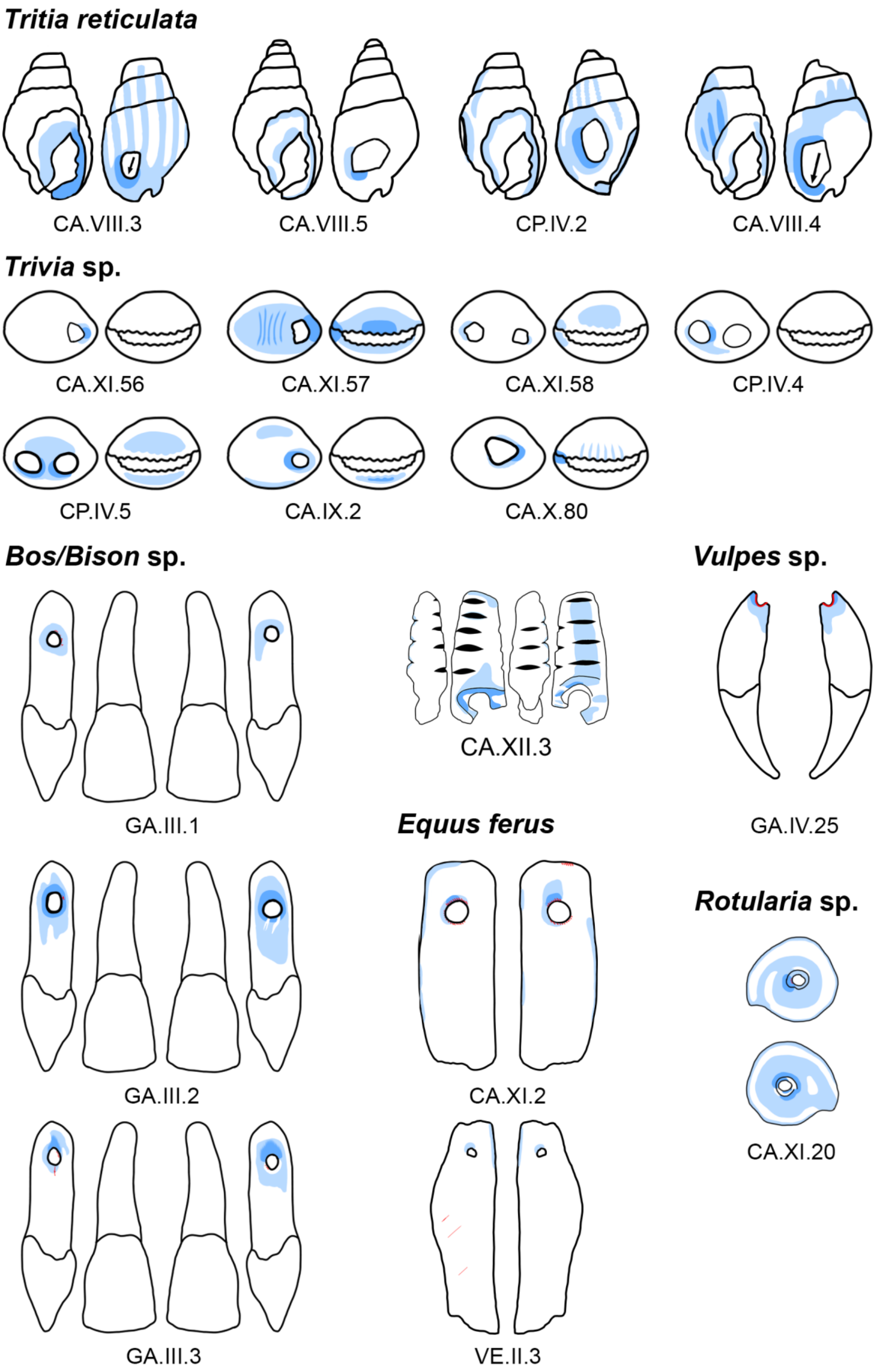
Tritia reticulata
CA.VIII.3
CA.VIII.5
CP.IV.2
CA.VIII.4
Trivia sp.
CA.XI.56
CA.XI.57
CA.XI.58
CP.IV.4
CP.IV.5
CA.IX.2
CA.X.80
Bos/Bison sp.
CA.XII.3
Vulpes sp.
GA.III.1
Equus ferus
GA.IV.25
Rotularia sp.
GA.III.2
CA.XI.2
CA.XI.20
GA.III.3
VE.II.3

Cervus elaphus canines

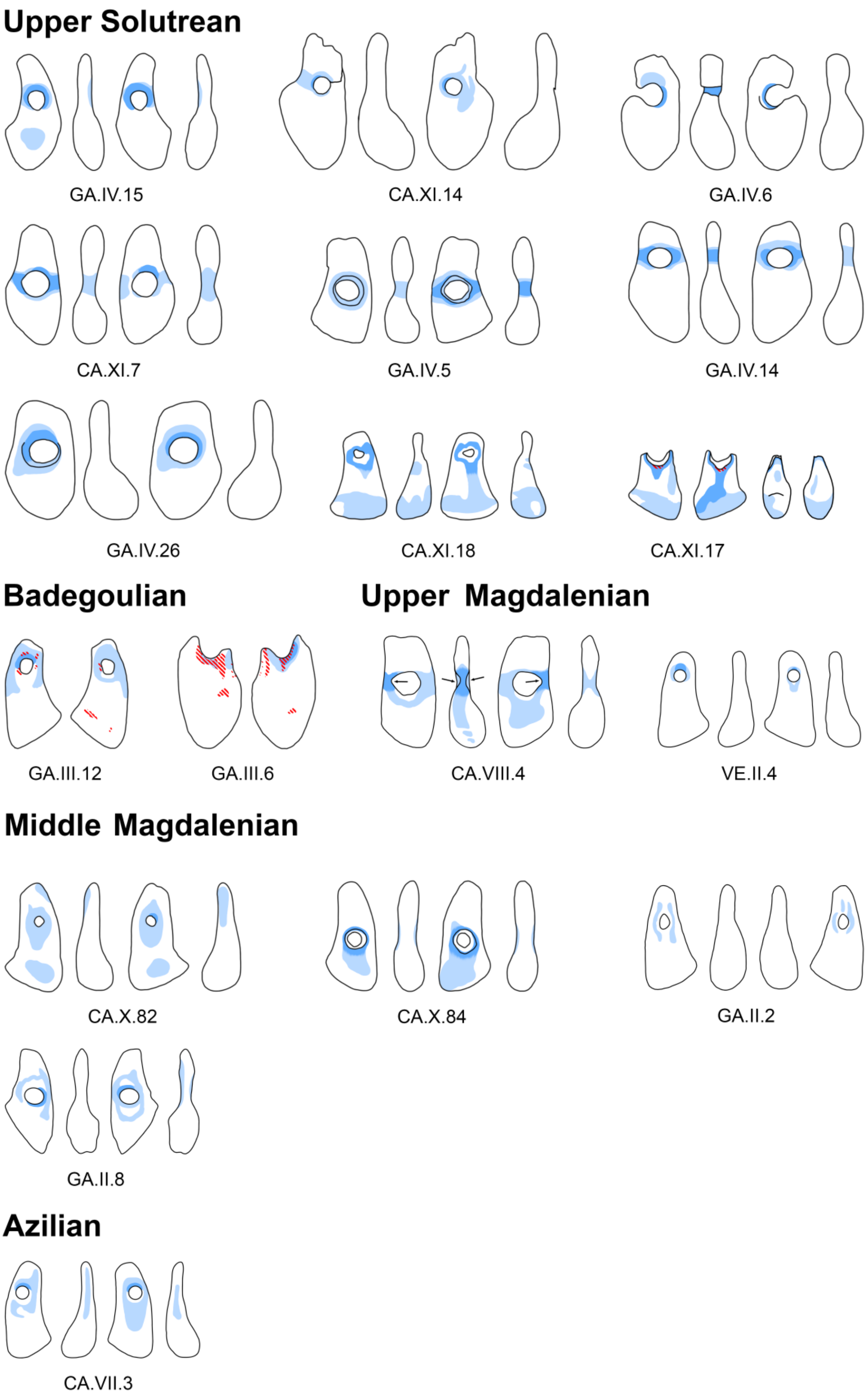

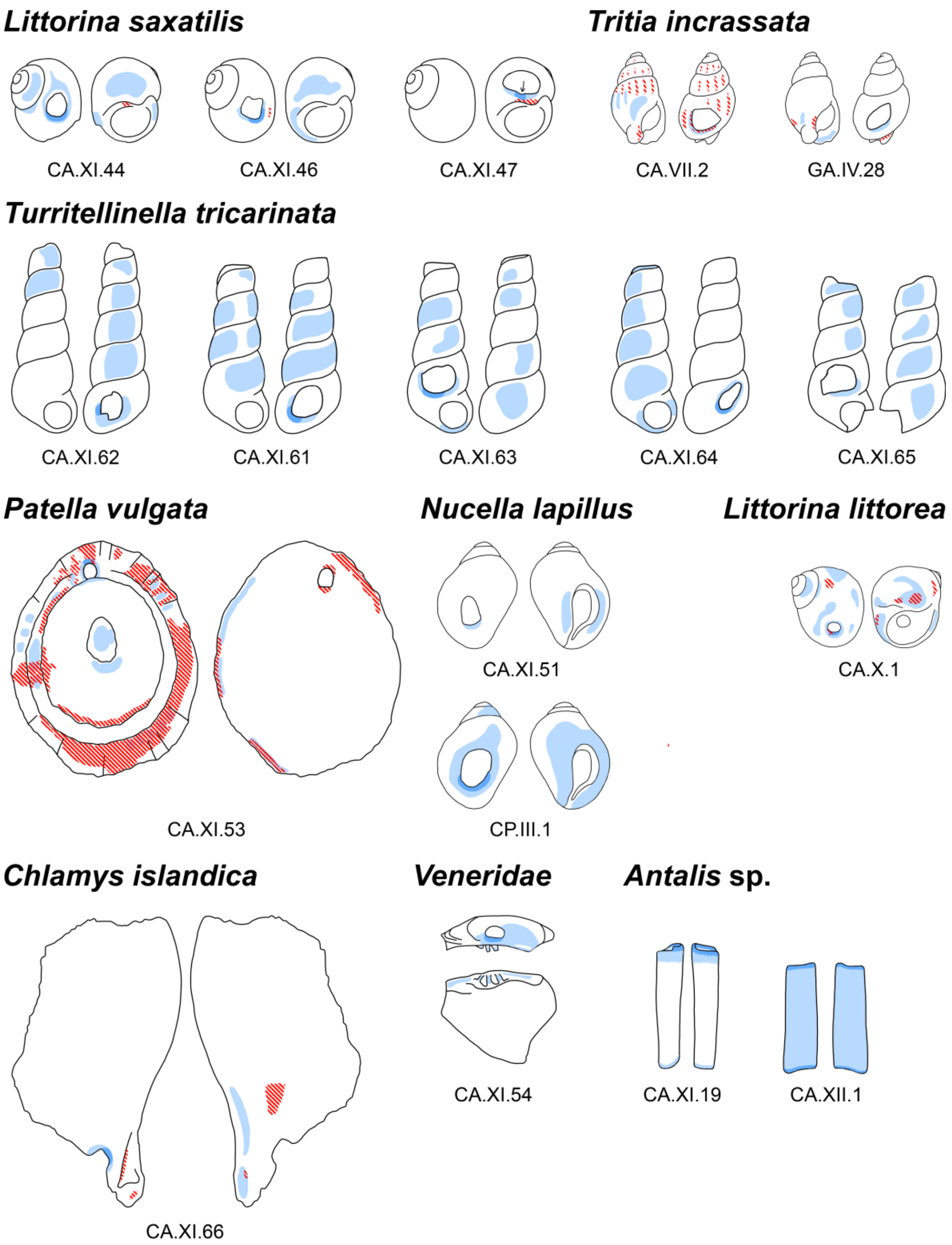
Littorina saxatilis
Tritia incrassata
CA.XI.44
CA.XI.46
CA.XI.47
CA.VII.2
GA.IV.28
Turritellinella tricarinata
CA.XI.62
CA.XI.61
CA.XI.63
CA.XI.64
CA.XI.65
Patella vulgata
Nucella lapillus
Littorina littorea
CA.XI.51
CA.X.1
CA.XI.53
CP.III.1
Chlamys islandica
Veneridae
Antalis sp.
CA.XI.54
CA.XI.19
CA.XII.1
CA.XI.66

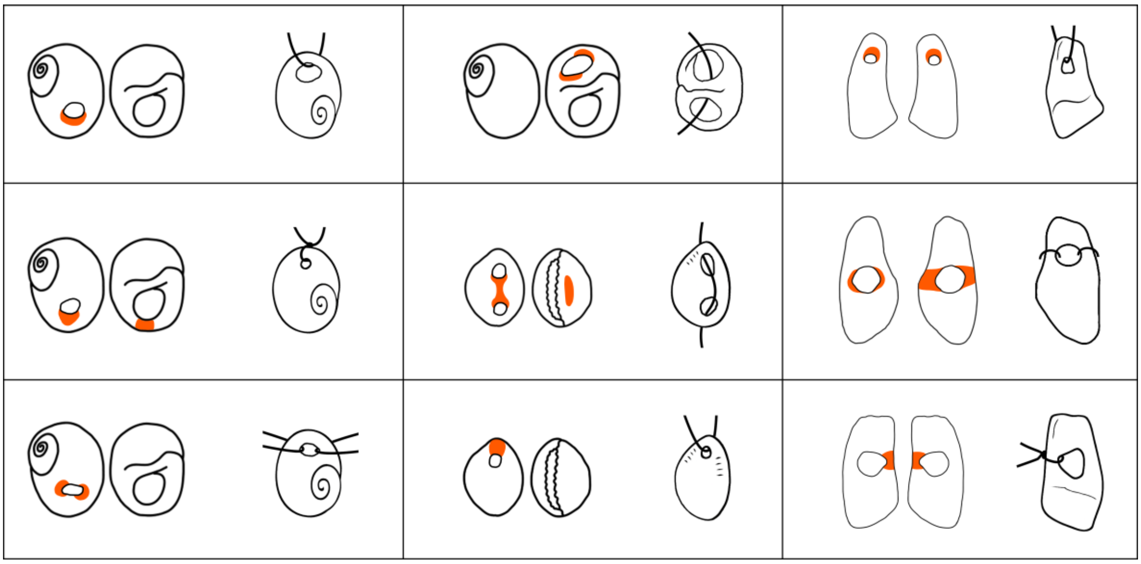

Supplement: S10 Fig — (ZIP) [file pone.0351170.s008.zip › S10 Fig.pdf]
