# Supplementary material for: Identity and mobility through personal ornaments in Upper Paleolithic cantabrian hunter-gatherer societies: Insights from Llonín cave (Asturias, Spain)
Source: PLoS One. 2026 Jun 8;21(6):e0351170. doi: 10.1371/journal.pone.0351170 (PMC13245794; doi:10.1371/journal.pone.0351170)
Supplement: S2 File — The figure below illustrates the suspension modes employed during the use-wear experiments. Suspension durations ranged from 1,000 hours to 6 months, depending on the specimen. In total, the manufacturing experiments comprised 260 beads, whereas the use-wear experiments comprised 373 beads arranged into 126 ornaments (S11 Fig). Detailed images of the manufacture traces of experimentally pierced teeth, shell and bone elements using the main techniques documented on the Llonín cave assemblage (S12 Fig). Evolution of use-wear in experimental shell and tooth ornaments, evidenced by polishing, rounding, enlargement, and deformation of perforation edges. Note how use-wear progressively obscures the technological traces of manufacture (S13 Fig). (ZIP) [file pone.0351170.s009.zip › S11-S13 Figs.pdf]

S11. Summary of the experimental collection employed during the analysis of the Llonín cave ornamental assemblage.

Experiments on manufacture techniques

SHELL									
		Abrasion	Rotation	Pressure	Percussion	Sawing	Flexion	Sawing + Flexion	Incision + Pressure
Gastropoda									
Littorina sp.	*L. obtusata/fabalis*	9	14	12	14				
	L. saxatilis								
	L. littorea								
Nucella sp.	*N. lapillus*	5	7	4	9				
Tritia sp.	*T. reticulata*	6	8	12	11				
	T. mutabilis	2	1	1	1				2
Trivia sp.	*Trivia sp.*	4	7	5	5				
Turritellinella sp.	*T. tricarinata*	5	5	5	5				
Patella sp.	*Patella sp.*		3	11	4				
Bivalvia									
Cerastoderma sp.	*C. edule*	5	5	5	5				
Ruditapes sp.	*R. decussatus*	5	5	5	5				
Scaphopoda									
Antalis sp.	*Antalis sp.*					7	9	7	

TEETH AND BONE							
		Abrasion	Rotation	Percussion + rotation	Sawing + rotation	Incision + rotation	Abrasion + rotation
Mammalia							
Canis sp.	*C. lupus*		3			2	1
Pisces							
Salmo sp.	*S. salar*	1		7	6		

Experiments on use-wear traces

USE-WEAR EXPERIMENTS					
		**Mechanical agitation**	**Natural suspension**	**Thread materials**	**Suspension modes**
Gastropoda				Leather	Paired (ventral/dorsal face)
Littorina sp.	*L. obtusata/fabalis*	164	96	Hemp	Separated by knots
Nucella sp.	*N. lapillus*	26		Horsehair	Free suspension (single bead)
Tritia sp.	*T. reticulata*	51		Braided sinew	Free suspension (3-4 beads)
Trivia sp.	*Trivia sp.*	27			Fixed (single bead)
Scaphopoda					Fixed (3-4 beads)
Antalis sp.	*Antalis sp.*	4	2		Braided from two points
Mammalia					Internal transversal thread (Trivia)
Canis sp.	*C. lupus*		3		External transversal thread (Trivia)

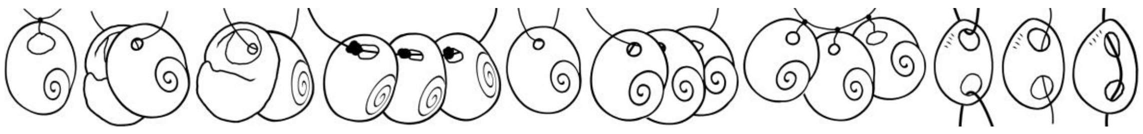

S12 Fig. Detailed images of the manufacture traces of experimentally pierced teeth, shell and bone elements using the techniques documented on the Llonín cave assemblage.

ABRASION

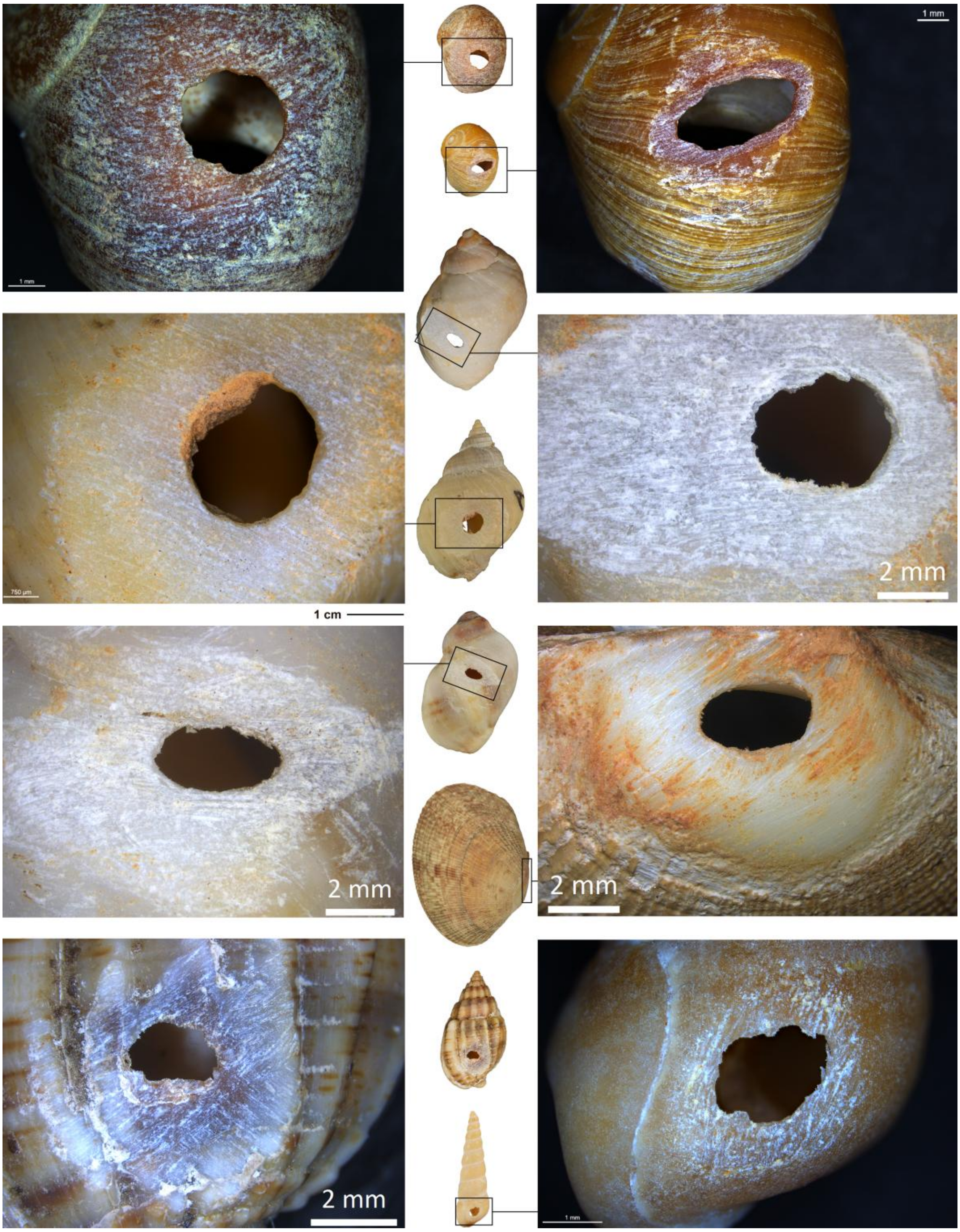

ROTATION

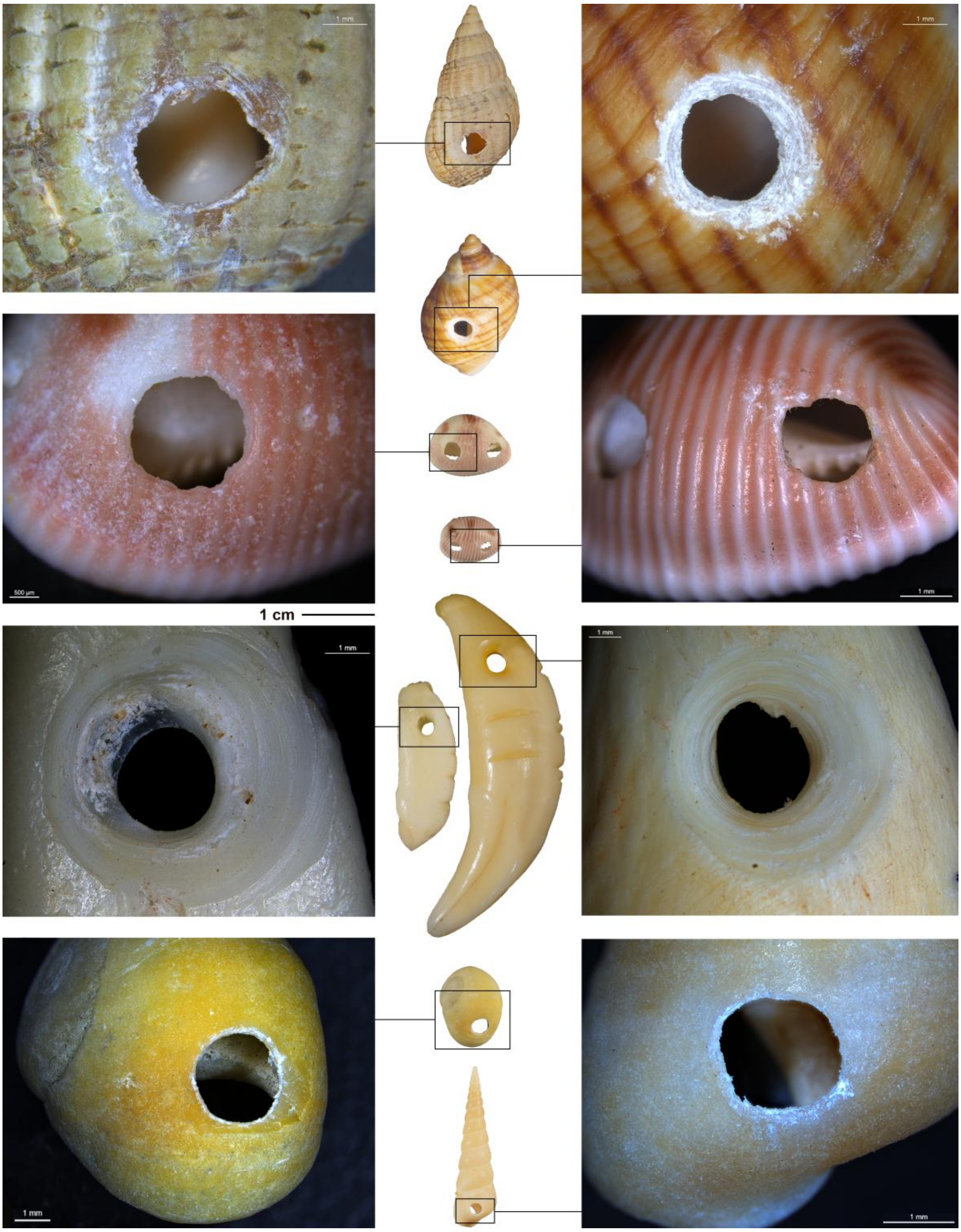

PRESSURE

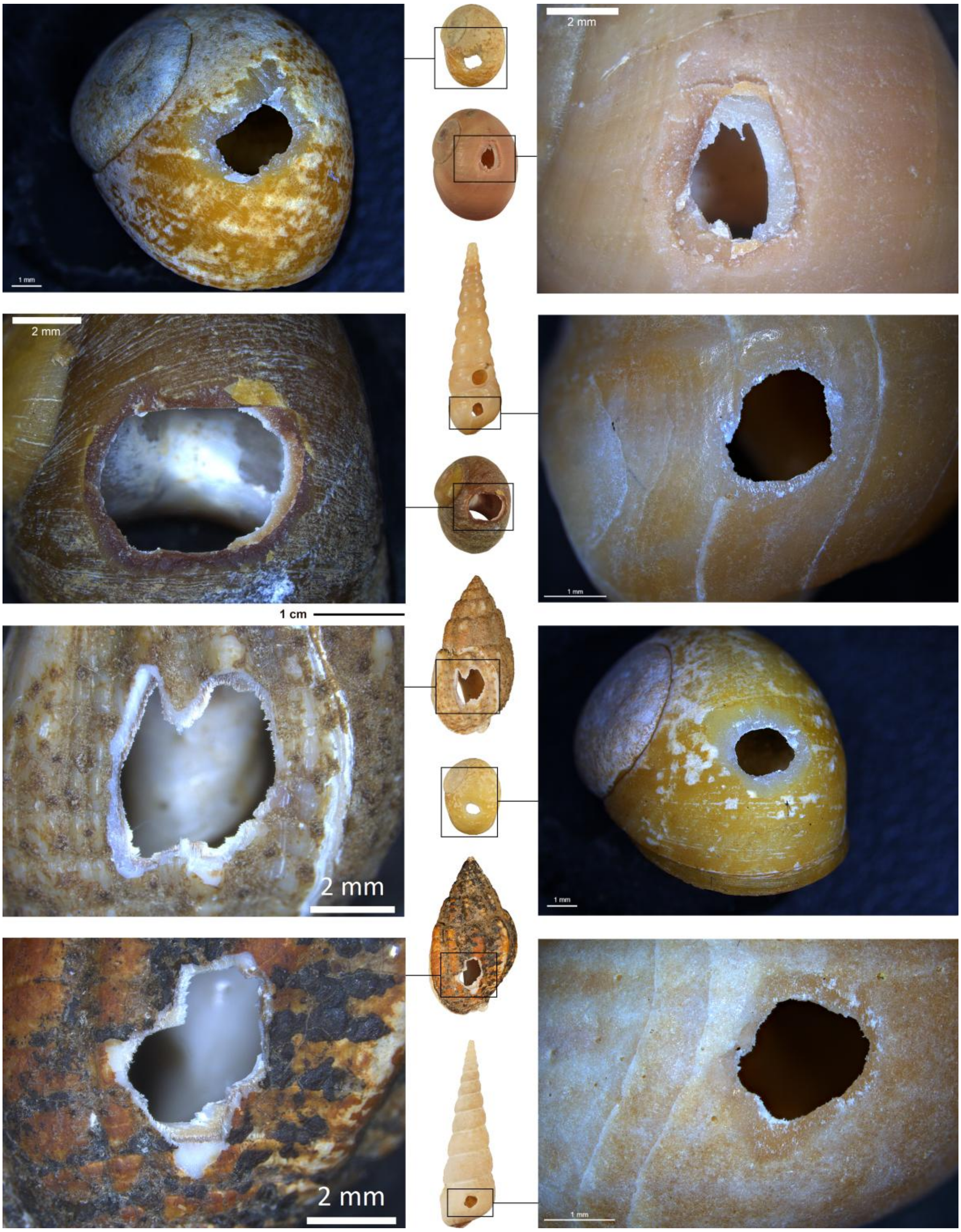

INDIRECT PERCUSSION

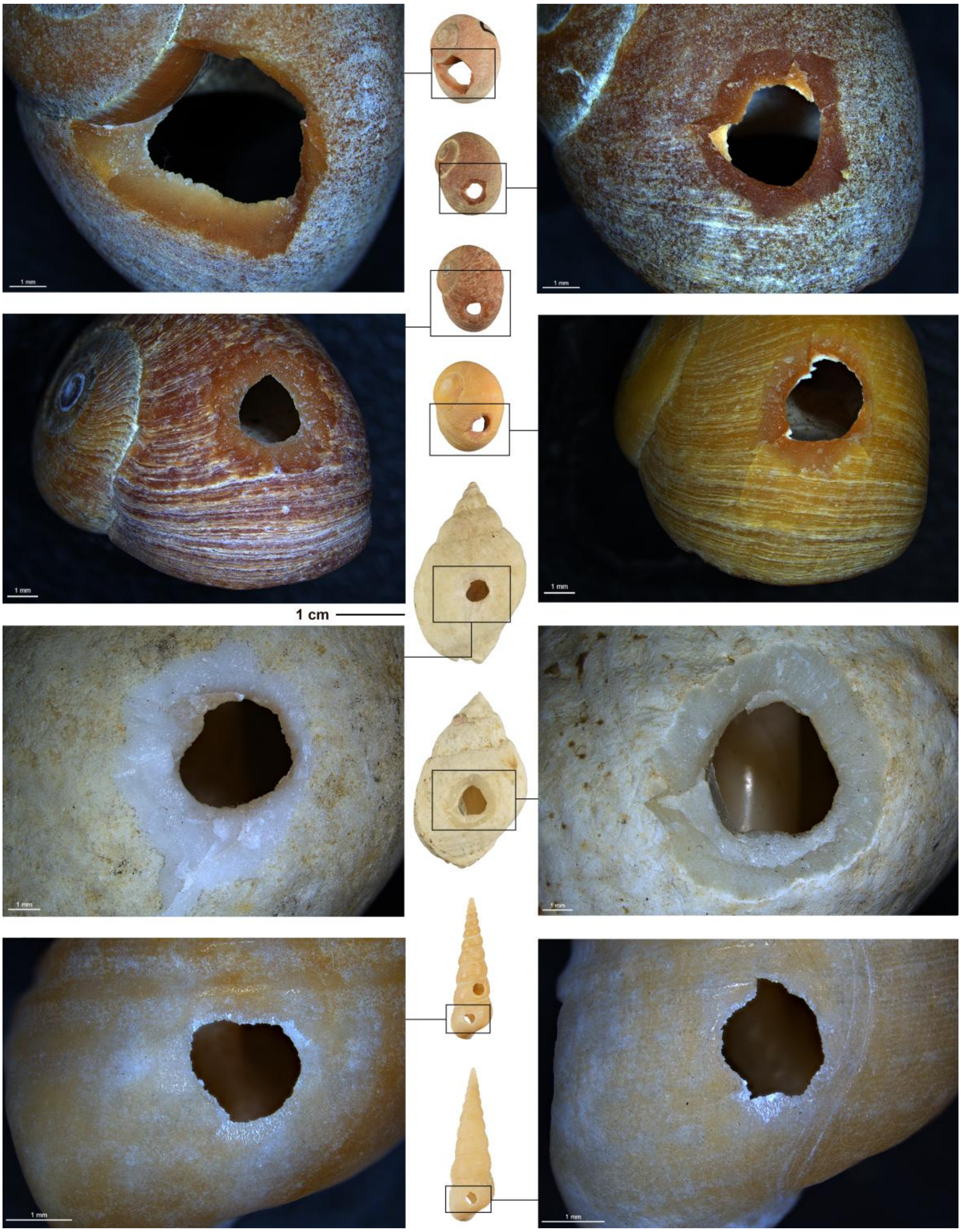

INCISION + PRESSURE

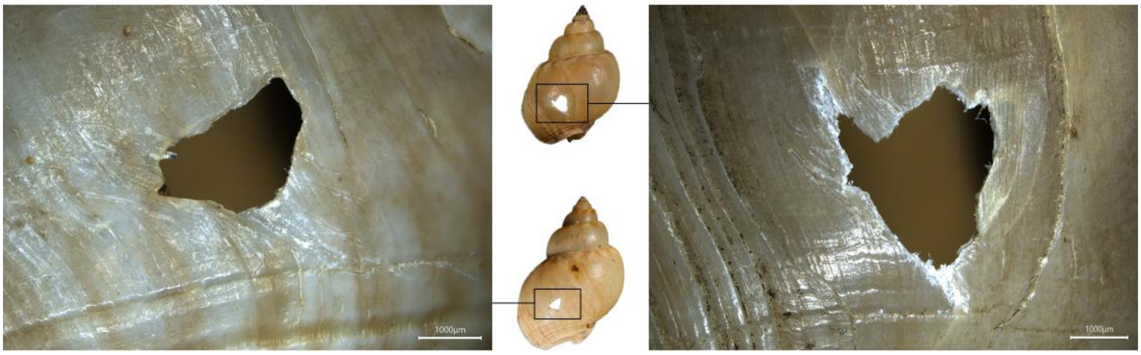

FLEXION

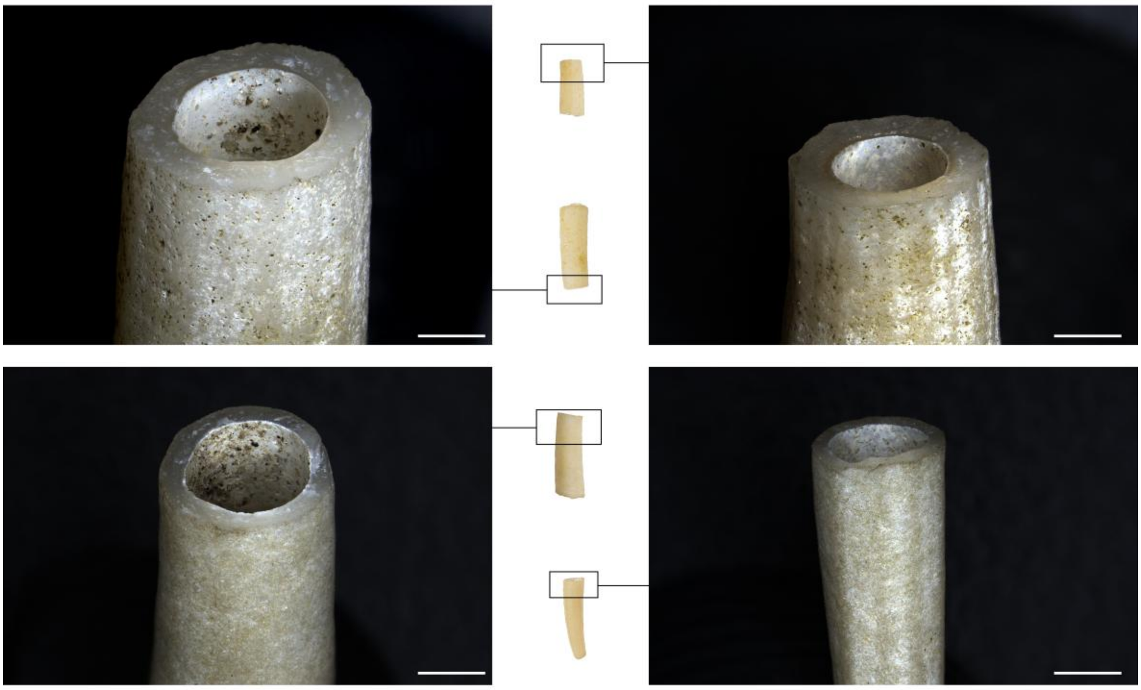

SAWING + FLEXION

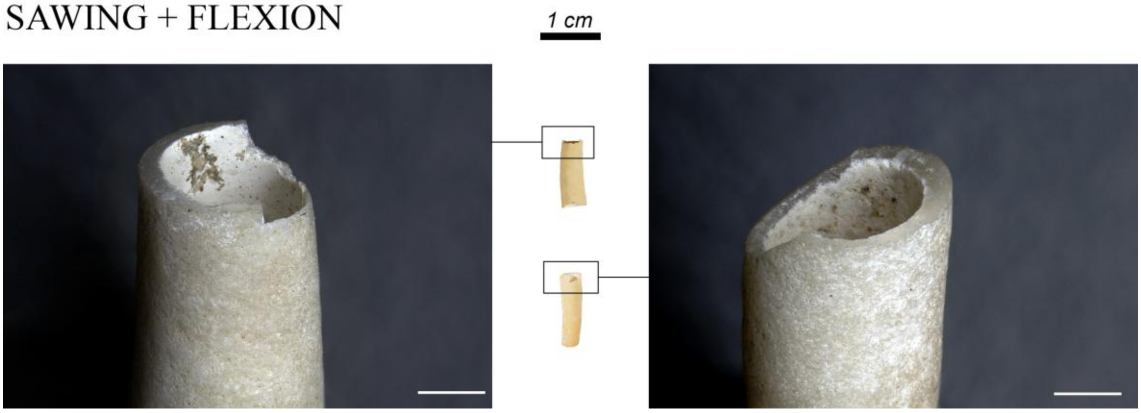

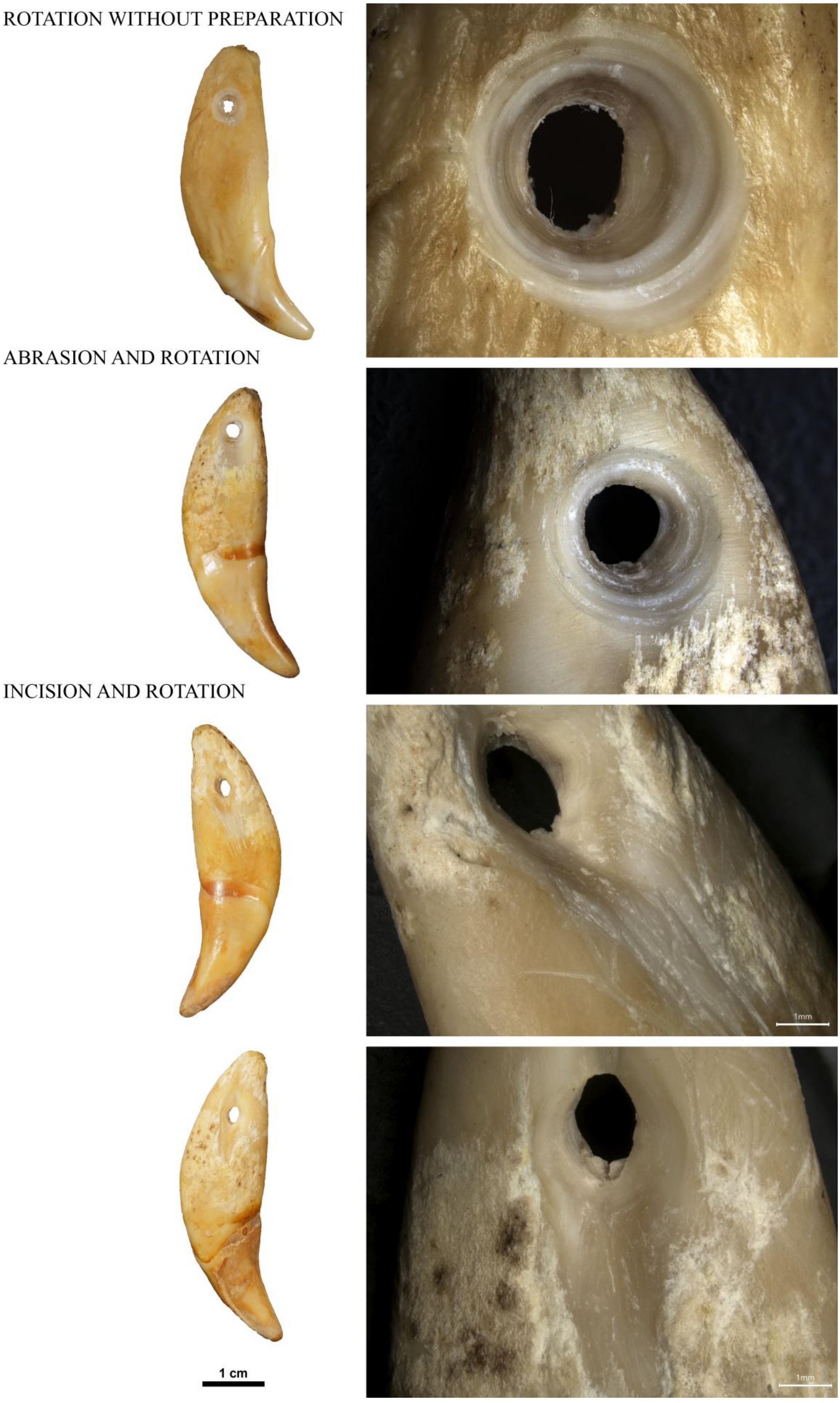
ROTATION WITHOUT PREPARATION
ABRASION AND ROTATION
INCISION AND ROTATION
1 cm
1mm
1mm

PERCUSSION + ROTATION

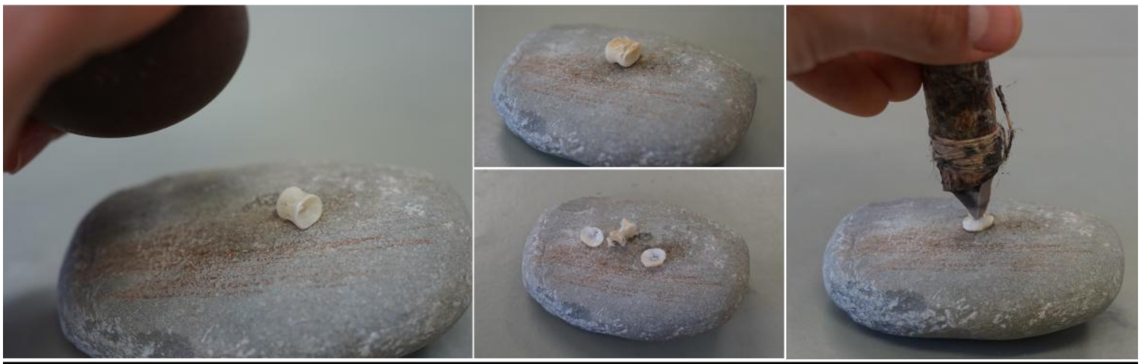

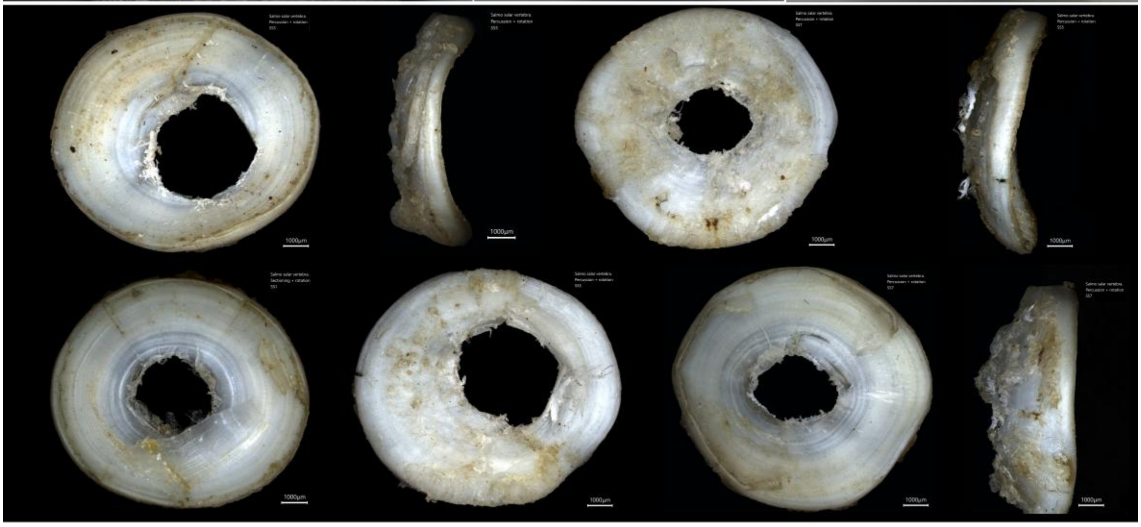

SAWING + ROTATION

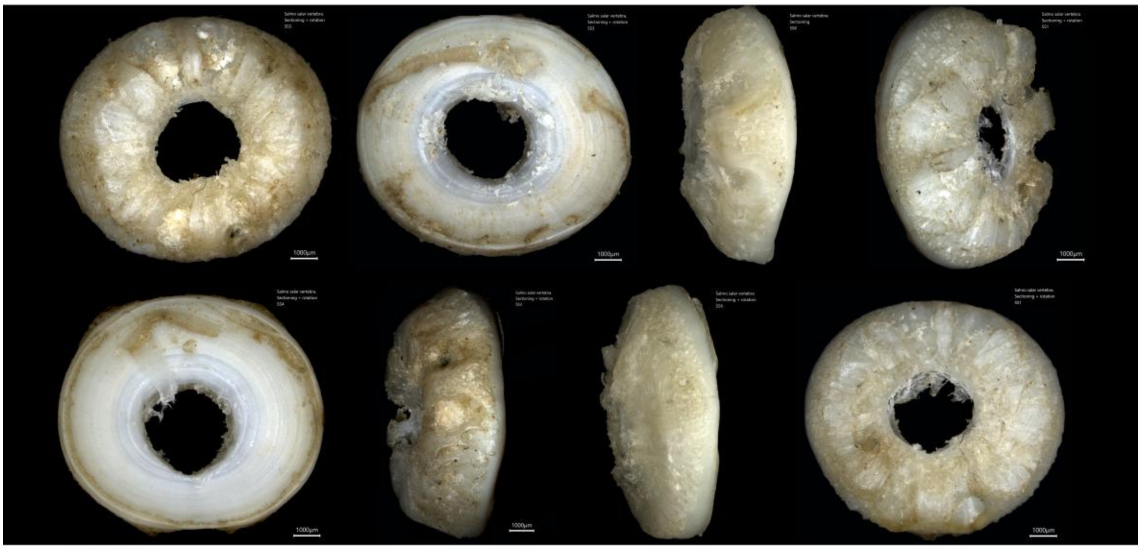

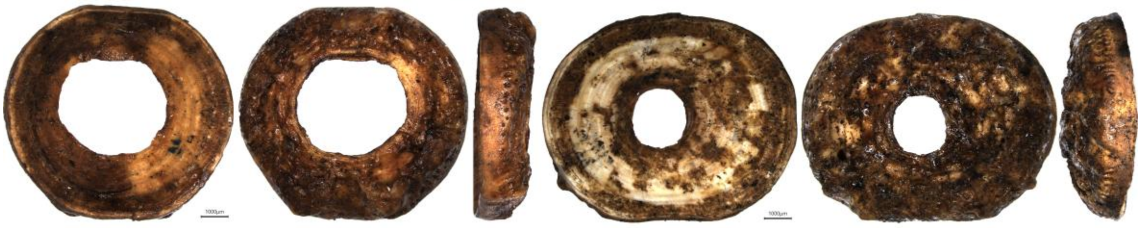

GA.IV.1 GA.IV.2

S13 Fig. Evolution of use-wear in experimental shell and tooth ornaments, evidenced by polishing, rounding, enlargement, and deformation of perforation edges. Note how use-wear progressively obscures the technological traces of manufacture.

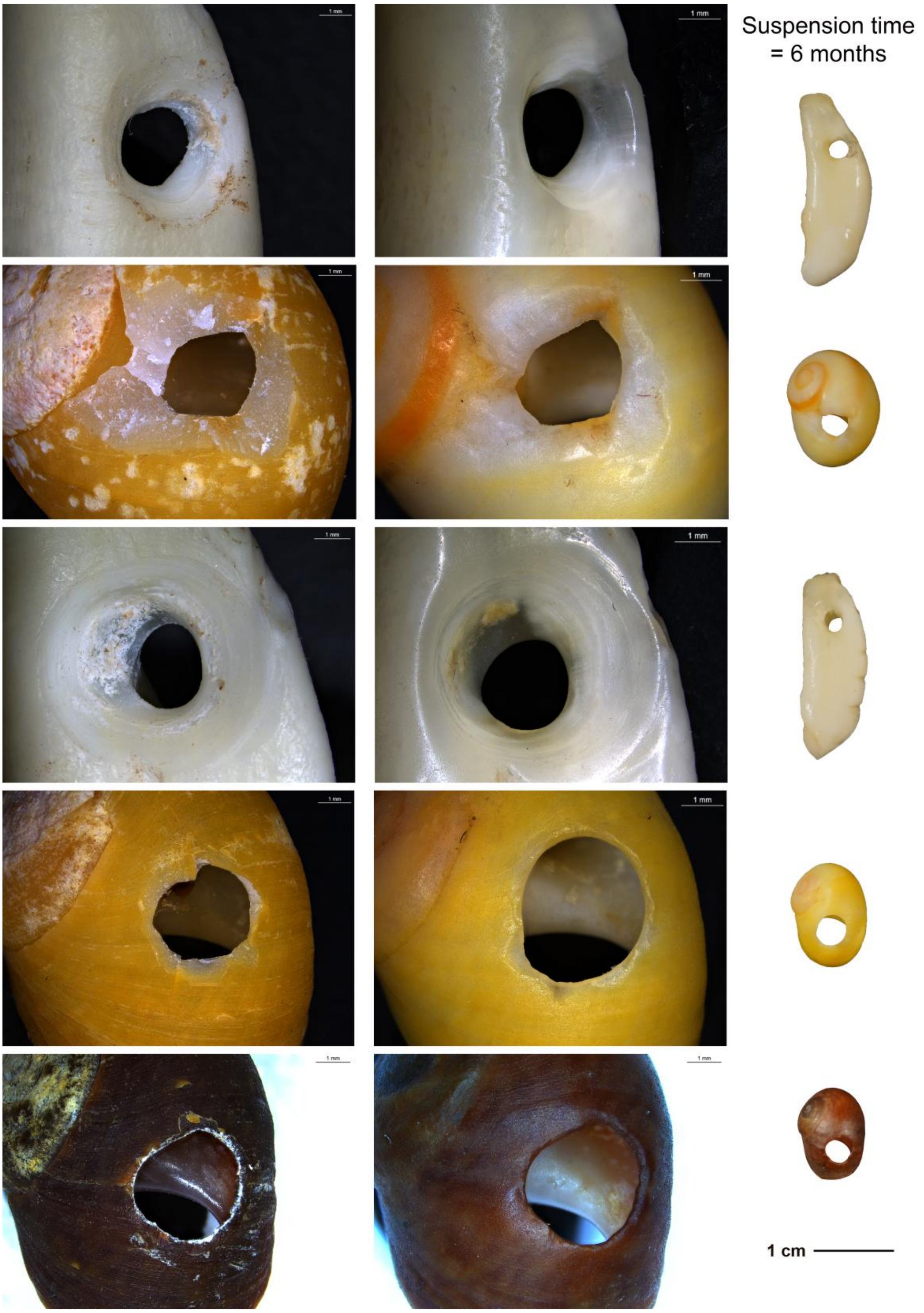

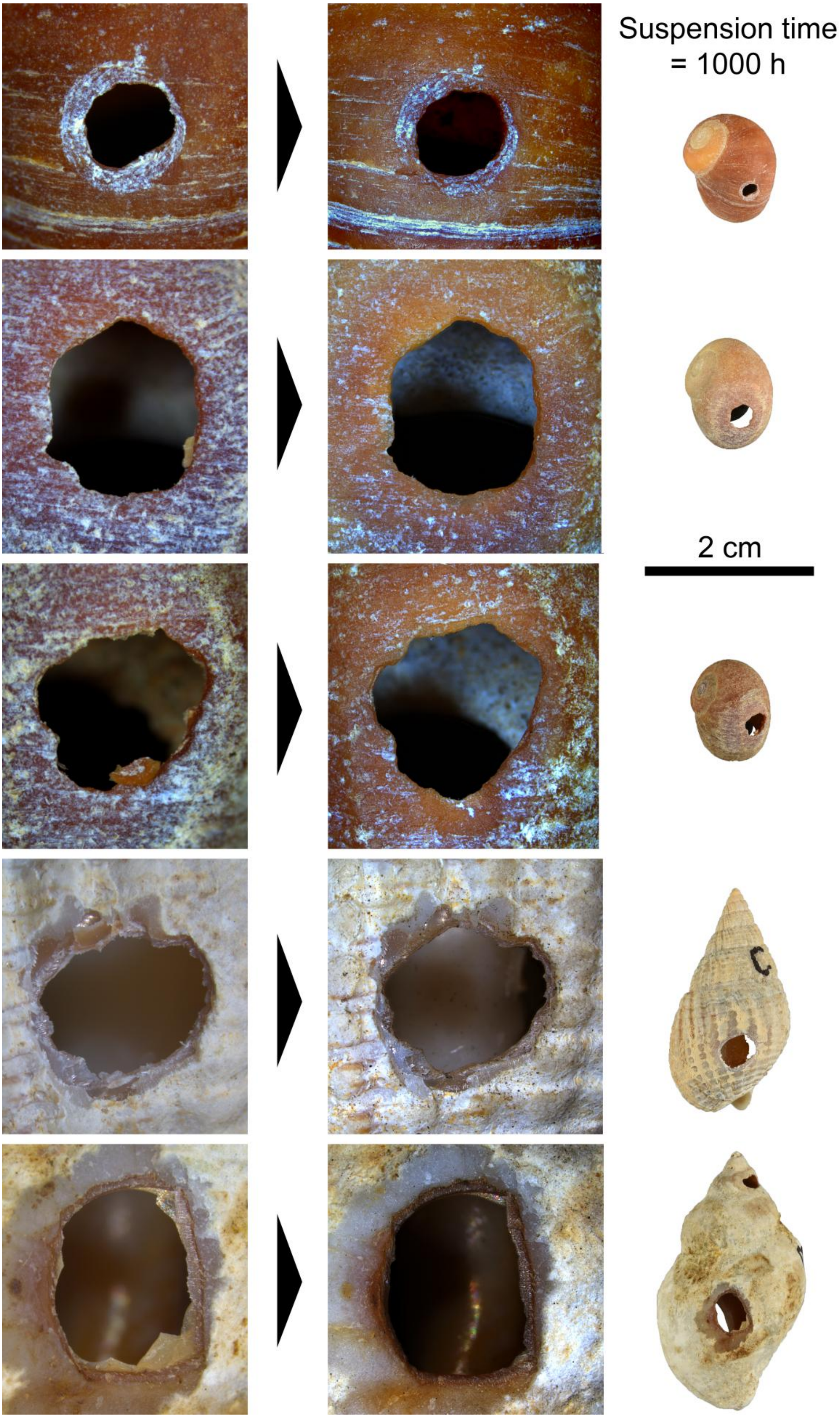
Suspension time
= 1000 h
2 cm
