# Supplementary material for: Identity and mobility through personal ornaments in Upper Paleolithic cantabrian hunter-gatherer societies: Insights from Llonín cave (Asturias, Spain)
Source: PLoS One. 2026 Jun 8;21(6):e0351170. doi: 10.1371/journal.pone.0351170 (PMC13245794; doi:10.1371/journal.pone.0351170)
Supplement: Attachment — Submitted filename: Response to Reviewer 2.pdf [file pone.0351170.s012.pdf]

RESPONSE TO REVIEWER #2 [PONE-D-25-62247]

We are deeply grateful for the reviewer's comments and suggestions, and for the great interest shown on our work. We consider that these observations have significantly contributed to improving the manuscript, and we sincerely hope that our responses, as well as the corresponding revisions made to the manuscript, figures, and Supporting Information, meet the expectations and requirements raised. We take this opportunity to report several updates regarding the materials under study. Two new items have been incorporated following a revision of the material collections: a fragmented specimen of Trivia and a perforated goat premolar, although the latter lacks a clear chronological attribution. The most noteworthy aspect concerns the reassessment of two specimens, now identified as Tritia mutabilis. This represents a relevant contribution to the discussion of mobility, as this taxon is exclusively Mediterranean. During the revision process, we were able to acquire several comparative specimens for our reference collection, which allowed us to confirm this attribution and to conduct a series of manufacturing experiments. Another aspect to be noted is the reorganization of the Supporting Data, carried out at the explicit request of the other reviewer. The S_data are now organized by material type, such that each table contains all the consolidated information (biometry, technology, taphonomy, etc.). A detailed, point-by-point response to the reviewer's original comments is provided below.

Review PONE-D-25-62247

This is a very interesting paper that documents the diachronic evolution of ornamental practices as well as their social role and the functional aspects of the site. The conclusions are very interesting and integrate the information from the symbolic sphere into the overall functioning of late Paleolithic societies, which gives this study significant scope. The supplementary materials are particularly valuable and provide access to all the raw data.

I recommend publishing this article with moderate revisions.

I find this work very rigorously done, but I have a few suggestions, particularly about the traceological section, which I think would benefit from a few clarifications. There are also some questions out of curiosity, the answers to which the authors can include in their paper if they wish to provide details.

<u>Methodes</u>
Taxonomic and biometric analyses

- L. 128: you indicate the use of "specialized literature", but there is no bibliography associated. Could you indicate the references?

The requested modifications have been implemented

Morphometric and use-wear analysis

- L. 180: you cite S2 Table following a statement about the experimental collection, but the table lists data on archaeological red deer canines.

The requested modifications have been implemented

<u>Results</u>
Taxonomic analysis

- L. 236-238: Can you specify which teeth were found for each species (canine/incisor/molar, etc.)?

The requested modifications have been implemented

Taphonomic analysis

- L. 266: same problem as in line 180, S2 table does not provide taphonomic information as it only lists data on archaeological red deer canines (I do not see a “taphonomic alteration” column).

The requested modifications have been implemented

- I think that some aspects discussed in the section on “taphonomic analysis” could be integrated differently:
    - L. 268-269: I think this is a matter of debate, and not everyone agrees. I completely understand if you disagree. However, I don't know if thanatocenosis alterations should be mixed with alterations related to diagenesis (in the context of an archeological study, which aims to understand the role of ornaments in past societies, it is obviously different in paleontology), since they do not provide information about the same stages in the life of objects. I'll leave it up to you to decide whether you want to include this or not.
    - L. 275-277: I believe that extraction marks on teeth should not be included in the category of taphonomy but rather in the manufacturing/acquisition process, as they are part of the technical actions that humans have used to transform animal material into symbolic objects.

In the Discussion section (“Raw material procurement”), cut marks are explicitly linked to acquisition processes (line 509 of the original manuscript). In addition, the second reviewer has specifically requested a clearer distinction between pre-collection taphonomic processes, anthropogenic modifications, and depositional processes, with cut marks included within the anthropogenic category, so, in order to ensure consistency and accommodate suggestions from both reviewers, we have opted to retain cut marks within the framework of anthropogenic taphonomic processes, while making their direct relationship with raw material procurement strategies explicit. Regarding the first comment, we have decided to rearrange the order of the taphonomic alterations, ordering them by these categories: pre-collection alterations, anthropic alterations, and post-depositional alterations.

- L. 271-275: Are you sure that this is sediment-related impregnation? In the photographs (which are very good quality!) that you have provided, iron oxides appear to be quite clearly under the sediment and encrustations. This may not be the case for all the pieces, but this might be evidence of deliberate coloration of some of the shells at the site. I am thinking in particular of photograph P, on which iron oxides are relatively concentrated in the natural depression of the shells, with encrustations (that does not contain oxydes) that partially cover the deposits. For my part, I would use these arguments to assert that certain elements have been colored, but if there are arguments against this interpretation, it could be added to the text.

Thank you for highlighting this point. The specific case you mention, *Patella vulgata* CA.XI.53, deserves particular attention. This specimen is heavily impregnated with ochre beneath the concretion, although not uniformly across its surface. We suggest that it may have been used as a tool for pigment processing—an activity documented at several Cantabrian sites studied by David Cuenca, such as El Juyo or Altamira—which could explain the strong presence of ochre. However, the preservation state of the specimen’s edges does not allow us to confirm or refute this interpretation, which is really unfortunate. Regarding the rest of the assemblage, it is true that, when considered as a whole—and largely thanks to the schematic drawings showing the distribution of ochre and use-wear—a tendency can be observed for ochre to accumulate within the grooves of the shell spirals, as well as on surface areas polished through use, where it has been preserved within the micro-pores of the shell surface. Since the artefacts were washed after recovery (during excavations in the 1980s–1990s), it is possible that some of the original ochre residues were lost. In any case, a brief note has been added to the taphonomy section to acknowledge this possibility.

- L. 277-279: It would be interesting to explain the characteristics that led you to conclude that this was accidental exposure (uneven color? Micro-flaking? Uneven cracked appearance? etc.). The item you show in fig. 6G appears to have changed color to a uniform shiny black, which I think could mean it is possible that it was deliberately heated to change color.

In the Llonín assemblage, three atrophied canines with comparable characteristics have been identified (GA.III.9, GA.III.14, and GA.III.7). One of these is a modified ornamental piece, while the other two lack any evidence of perforation. The ornamental specimen appears to have undergone fracturing as a result of thermal alteration, as the internal surfaces of the fractures display the same black coloration. By contrast, the other two specimens remain complete, although their external surfaces are notably irregular and exhibit small thermal pitting. Given that all three specimens derive from the same stratigraphic context—Level III, spit 14, squares B4 and B5—within an area characterized by a high density of hearths, we consider it more likely that this coloration results from incidental exposure rather than deliberate treatment. We nevertheless intend to address this issue in future work through experimental testing of thermal alteration in atrophied canines and other osseous elements.

Use-wear analysis

- L. 306-308: Are there signs of wear on these naturally perforated pieces? This would prove that paleolithic humans did indeed take advantage of these natural perforations. It would also be interesting to share the criteria you used to determine that the perforations were of natural origin (based on d'Errico et al., 1993?).

As shown in the supplementary dataset associated with the shell elements, of the three specimens perforated by lithophagous organisms, two appear to show no evidence of use. The third specimen, however, may exhibit use-wear traces; nevertheless, the presence of concretions within the perforation prevents a definitive assessment.

Production of ornaments

- L. 321: I do not understand technically how abrasion can be used to widen the medullary canal of the vertebra. The lack of photographs and of description of the traces makes it difficult to understand the technical gesture.

We acknowledge that the initial attribution of abrasion as the manufacturing technique for the salmonid vertebrae was based on personal communications regarding comparable beads from the Cantabrian region, such as those documented at Las Caldas cave. This review prompted us to develop an experimental protocol aimed at testing this hypothesis. The results demonstrate that abrasion is not a viable technique for producing morphologies consistent with those observed in the archaeological record. Based on these results, we conclude that the most likely manufacturing sequence involves transverse sawing combined with rotational perforation. We have also documented an alternative method for separating the articular surfaces of vertebrae through percussion; however, the morphologies produced by this technique do not match those observed in the archaeological specimens. Data and images derived from this experimental protocol have now been included in the Supplementary Information (S11–S12).

- L. 344: I'm not sure I understand what technical gesture you mean by "trimming." Could you elaborate?

We used the term "trimming" as the action of cutting or shaping the edges of an object to achieve its final form, or removing excess material to refine a piece. We considered it appropriate to describe the process of crafting a rondelle, given the experimental procedures shown in some publications like Needham et al (2019). However, if the reviewer considers it imprecise or inappropriate we would be happy to change it.

- Fig 7: There is an error in the caption; figure L is not the tooth of *Capra pyrenaica* (I assume it is M).

The requested modifications have been implemented

- L. 371-382: I think this description of your identification criteria should appear at the beginning of the section on perforation techniques so that readers know on what grounds the techniques are identified in the rest of the section. However, I believe that morphology alone is not sufficient to determine the techniques. I suggest that you include the section of perforations, striations, and removals as presented in (Tátá et al., 2014), and explain at the beginning of the section on "production of ornaments" which sets of criteria correspond to which techniques.

Indeed, there are numerous factors and characteristics that define each manufacturing technique and allow for their precise differentiation. We have relied extensively on the framework established by Tatá et al. (2014) regarding perforations, traces, striations, bevels, perforation profiles, morphologies, and related features. We considered it unnecessary to include all these details (such as the well-known photographs of perforation sections), as providing the bibliographic reference in the methods section was deemed sufficient. Nevertheless, we have addressed the reviewer's suggestion by adding a few lines in the results section on manufacturing techniques, highlighting the characteristic elements of the documented techniques, and linking them to the Supplementary Figures illustrating some of the experiments conducted.

- General question: given that the object are worn, as you mention later in your article, does this not interfere with the observation of manufacture traces? Could you add a sentence about this potential obstacle?

The effect of use-wear on our analyses has been limited for several reasons. First, Magdalenian ornaments generally exhibit low levels of use, so that diagnostic features indicative of the various manufacturing techniques are sufficiently preserved to allow their reliable identification. Second, even in those specimens showing more intensive use-wear, enough diagnostic elements—such as inward- or outward-facing bevels on the shells, scraping marks, and smoothed abrasion surfaces—remain to permit accurate reconstruction of the production techniques.

The most heavily used elements are certain atrophied canines, in which the biconical shape of the perforation is still retained, allowing us to confirm that rotational techniques were applied. Nevertheless, in these cases, unless faint traces of incisions are preserved, it has not been possible to determine the specific preparatory technique employed prior to rotation, if any.

Use and suspension systems of personal ornaments

- L. 389: I think that the attenuation and erasure of technological traces is an identification criterion that works for all types of materials, as suggested by S. Bonnardin or M. Mărgărit (Bonnardin, 2008 ; Mărgărit, 2016)

Indeed, and it's of course a criterion that we applied to all types of materials when we analyzed the assemblage. We have therefore specified this on the manuscript and also added the corresponding references.

- L. 405: When you say "no difference in use," does that mean that the type of traces and their organisation on the different material is identical, or simply that all types of materials show use-wear?

Our intention was to indicate that both materials show similar proportions of used and unused elements. The sentence has been revised to avoid potential misinterpretation.

- L. 407-409: Could you describe what led you to this conclusion? In other words, could you describe the traces in question in detail, explaining how they are more or less developed depending on the item considered?

Our intention was to indicate that, within the subset of specimens showing no evidence of use, more than 70% correspond to the Upper Solutrean, whereas in later periods such specimens occur only sporadically. Regarding the degree of use, for example, the Solutrean assemblage includes atrophic canines displaying a wide range of use-wear, from very lightly used specimens to others with perforations heavily deformed through prolonged use. In contrast, use-wear traces in the Middle Magdalenian canines are generally less developed (see S10 Fig). A similar, albeit less pronounced, pattern can be observed among *Littorina obtusata/fabalis* specimens. An exception to this trend is the canine CA.VIII.4 from the Upper Magdalenian, which likely exhibits the highest degree of use within the assemblage. Finally, concerning the quantitative aspect, we refer to the current inability to establish direct equivalences or to formulate robust hypotheses regarding the duration of suspension of the ornaments. Although experimental work aimed at quantifying use-wear is currently underway, it remains too preliminary to support reliable inferences.

- L. 411: The bibliography you present on this matter is limited. Even though the literature has greatly expanded in recent years, I think it is possible and preferable to cite a more comprehensive list of references (suggestions at the end of this document).

The bibliography has been expanded accordingly to the reviewer's request. Thank you very much for the recommended bibliography, it has been really useful, and I really enjoyed particularly the one written by Osipowicz et al 2020.

- L. 417-418: This may be a misinterpretation on my part, but it seems in this sentence that you are suggesting that *Antalis* beads can only be arranged in one type of composition. I agree that there is only one way to thread a string through a tubular bead such as *Antalis*, however there are many ways to integrate this into various compositions such as sawing as suggested in (Vanhaeren et D'Errico, 2001) or individually tied as tested in (Hoareau et Beyries, 2022).

We meant that the only way to thread a string is through the hollow part of the shell, but we acknowledge that the formulation was confusing, so we have added a sentence clarifying this issue, also with the necessary references attached.

- L. 422: Why buttons? Why not just a simple knot or sawing, with only one side of the tooth against the wearer? I don't know of any experiments involving buttons, but I would assume that the friction of the element that closes the button would affect the entire surface, not only one side.

In the specific case of specimen CA.VIII.4, we have proposed its possible use as a button due to the extremely pronounced deformation along the horizontal axis of the perforation, which significantly alters its morphology, giving it a tear-drop shape. Such a localized and marked deformation suggests a relatively rigid fastening system subjected to greater tension than is typically observed in other archaeological or experimental specimens, leading us to consider that an element functioning as a button would logically be exposed to increased tension and friction. We are currently conducting use-wear experiments on dental elements and, hopefully, these will allow for more precise inferences in the future. For the time being, we have slightly expanded the explanation regarding this specimen and have made the hypothetical nature of this interpretation more explicit in the manuscript.

- Fig. 10: Some of the photographs do not allow the reader to follow the reasoning leading from the traces to the reconstruction hypothesis. May I suggest at least including supplementary materials showing the array of traces that lead to each of the reconstructions? I am thinking in particular of hypotheses D, E, and F, which must be based on a set of relatively complex evidence that are difficult to summarize in one photograph. Furthermore, are these hypotheses

based on comparisons with the experimental collection? Can you show photographs of the experimental traces?

Following a comprehensive re-examination of the material during the preparation of the use-wear distribution drawings, we have reorganized figure 8 by adding some new photographs and slightly simplifying the proposed suspension modes. After reconsideration, and due to insufficient supporting evidence, we have discarded suspension mode F previously hypothesized for one of the Trivia specimens. We have also opted to retain a fixed coding system for suspension modes (A–J), as this facilitates direct correlation with the archaeological specimens, which share the same lettering system. We acknowledge that it is challenging to condense all this information into a single figure; therefore, we have selected between one and three photos to support each case (also considering the constraints on image format and size). We believe that the revised set of drawings, specifying the location of use-wear traces, allows for a clearer and more accurate interpretation of the figure.

- General: Are there suspension modes linked to a particular type of shell? How many elements correspond to each suspension mode? What is the array of evidence that lead to one interpretation rather than another?

Most of the gastropods documented exhibit free suspension through perforations located on the labral edge, with the exception of a few specimens perforated in position E3 or showing a fixed, knotted suspension system. We indicate in the manuscript that certain taxa are associated with specific suspension systems, as is the case for Antalis (due to its tubular morphology) or Trivia specimens with double perforations. Regarding the exact number of specimens per suspension system, we refrain from providing precise figures, as in many cases—although use-wear is clearly observable—the complete characterization of the perforations is hindered by the presence of concretions, fractures, and other alterations. Accordingly, we provide general guidelines for the assemblage as a whole and incorporate these considerations into the final assessment presented in the use-wear section (Results).

Decorated elements

- L. 442: Could the depth and length of the incisions be distinctive criteria?
    - Fig. 11 and l. 469: About the incisions on *Chlamys islandica* : it's up to you if you want to argue further that the incisions were not random, but I don't think the organization is very clear. Have you observed the bottom of the incisions? Secondary striations could indicate the use of a stone tool and help settle the question. If you don't want to expand on this part, I don't think it's a problem, as the photographic documentation is very good and should be sufficient for readers to develop their own interpretation on whether the arrangement is random or not.

We prefer, for the time being, to leave the interpretation of the potentially decorative nature of this *Chlamys islandica* valve open to the reader, supported by the photographs and tracing, as well as by reference to the decorated valve of the same species documented at Santa Catalina (Berganza et al., 2012). We do, however, intend to carry out a surface analysis using confocal microscopy in order to characterize and quantify the grooves (depth, length, profiles…) and their distribution as precisely as possible. As this analysis has not yet been completed, it will be addressed in future work.

<u>Discussion</u>

- L. 499: Here too, the dataset you refer to is that of archaeological deer teeth.

The requested modifications have been implemented

- L. 558-560: I disagree somewhat, as some of the visual arrangements can be approached through traceology (through experiments + ethnography, as in the bibliography at the end of the

document). I believe that more conclusive demonstrations could be achieved by conducting specific experiments based on the type of traces observed in the archeological assemblage (is that a perspective for the study of this site ?). On the other hand, I completely agree that it is impossible to know which part of the body was adorned without a well-preserved funerary context.

We fully agree with the first statement. In the manuscript, however, we were referring to the latter point, namely that, although the arrangement of the shells can be inferred through traceological analysis, it remains impossible to determine how they were positioned on the body or on objects without very specific preservation conditions. If the current wording is considered unclear, we are fully willing to revise it accordingly.

- L. 580-582: Considering the possible acquisition of objects through exchange, would this imply that objects circulated in their raw form, not perforated? Does this indicate a segmentation of the production chain that only existed during the Solutrean period?

We thank the reviewer for this insightful suggestion. It is indeed possible that some of the perforated—and also unperforated—atrophic canines documented in the Solutrean assemblage may have been acquired through exchange with other groups, particularly given the relatively high MNI, which exceeds that reported in archeozoological studies of long-term occupations in comparable contexts such as La Viña. In the case of unperforated specimens, this could imply a degree of segmentation within the production sequence. However, this pattern does not appear to be exclusive to the Solutrean, as unpaired, unperforated canines are also present in the Badegoulian (n = 8) and Magdalenian levels, albeit in smaller numbers. That said, the phenomenon is more pronounced during the Solutrean, which is also the only period for which clear evidence of in situ ornament manufacture has been documented. We present it as a hypothesis, but it's not possible to confirm it without doing isotopic analysis on the pieces themselves, an option we are contemplating for future works.

- L. 590: Is the type of functional use-wear the same? This could be decisive in identifying them as coming from a single composition. It also makes me think that, if possible, a dataset detailing the types of wear and their distribution on the pieces would give a precise idea of the compositions.

A new set of diagrams has been added to the supplementary information (S10 Fig) which includes the location of use-wear and ochre on each archaeological piece. For reasons of space and practicality, we have excluded from this set of schematic representations those specimens lacking clear chrono-stratigraphic attribution, as well as those without perforations, without evidence of use, or for which use could not be determined. Regarding the question about the bovine incisors, the use-wear is indeed coincident on the three specimens, which I have noted as well in the manuscript. Thank you for pointing it out!

- L. 593: To support either hypothesis, is there any data on lithic raw materials? I don't think you develop the topic of lithic material circulation for the badegoulian in the « cultural network and mobility » section.

The Discussion has been expanded to incorporate data on lithic raw material circulation, thereby complementing the interpretations previously developed for other aspects, including graphic representations, bone artefacts, and, ultimately, personal ornaments.

- L. 595: I think it's possible to expand on this a little further. I understand from this sentence that climatic conditions were theoretically just as difficult in the late Solutrean and Badegoulian periods, so there would be no environmental reasons to explain this difference. But there might be some evolutions at the local scale? Was there a change in the local landscape between the late Solutrean and Badegourian periods? Depending on whether it is possible to identify specific

environmental obstacles, this raises questions about cultural choices and the organization of networks: was there a reduction in the network of material circulation linked to adornment, but was the same true for other areas such as lithics?

The Badegoulian occupation at Llonín corresponds to GS-2.1c, at the end of the Last Glacial Maximum. However, the available climatic data for this and the other GS-2.1 sub-events remain relatively coarse for the Cantabrian region, and notable discrepancies can be observed between archaeological site records and natural proxies. In addition, variability in humidity has been documented not only between sites but also between different levels within the same site (e.g., Kiputz or Cobrante; García-Ibaibarriaga et al., 2019). From a sedimentological perspective, an increase in humidity during the Lascaux interstadial (Hoyos, 1995) likely led to karstic reactivation processes and inter-stratigraphic mixing, which complicate the techno-typological characterization of the Badegoulian in the Cantabrian region. In this context, the Badegoulian Level III of the Galería sector at Llonín constitutes a particularly well-preserved deposit, as it appears to have remained isolated from such reactivation processes, thereby explaining the relatively good representation of this period at the site. Regarding the lower number of ornaments during the Badegoulian, we suggest (and now explicitly state in the manuscript) that this pattern is more likely related to the function of the cave during each occupation phase rather than to climatic factors. While the Solutrean occupation is interpreted as more complex and residential in nature, the Badegoulian phase appears to correspond to a more specialized use of the site, closely associated with fire-related activities (e.g., smoking, drying, hide processing). A more detailed discussion of this aspect has been included in the S1 Text.

- L. 636 – 638: Is this stable throughout the entire chronology that interests you here?

The circulation of raw materials following this east-west axis from western Asturias to the Pyrenees occurs during the second half of the Upper Palaeolithic. As stated, we have expanded on the lithic raw material circulation in the section devoted to mobility, and we have cited several examples of this phenomenon in the different chronologies linked to the occupation of Llonín cave.

- L. 657: If you have any data on species evolution available, it would be interesting to cite it here, or even to discuss the contemporaneity of the evolution of species present on the coastline vs. those used by paleolithic groups.

In the Cantabrian region, the mollusk species used as personal ornaments remain remarkably consistent from the early Upper Paleolithic through to the end of the Mesolithic, largely corresponding to taxa that are still present today along Atlantic coastlines. It should be noted, however, that recent warming of the Cantabrian Sea has made some taxa, such as *Littorina obtusata* and *Trivia*, less readily available today. Throughout Cantabrian prehistory, the genus *Littorina* predominates, accompanied by a broader range of species present in lower frequencies, particularly *Trivia*, *Antalis*, and *Tritia*. In this sense, variation within ornament assemblages appears to reflect climatic fluctuations more clearly than taxonomic change, as indicated by the presence or absence of specific elements. For instance, ivory from *Elephas* (not represented at Llonín) is characteristic of colder, steppe-like conditions and disappears from the region during the Magdalenian. At the level of mollusks, the main climatic indicators in the Llonín assemblage are the presence of *Chlamys islandica*, a species currently associated with Arctic latitudes, and *Patella depressa*, which inhabits more temperate waters. Accordingly, during the Last Glacial Maximum in the Cantabrian region, only *Patella vulgata* is documented, whereas *Patella depressa* appears later, during more transitional phases towards the Holocene. In Llonín this fluctuation is clearly visible, as *Chlamys* occurs during the Upper Solutrean, and *Patella depressa* during the Upper Magdalenian. To address this topic in greater detail, we have added a Supplementary Text (S2 Text) providing information on the ecological niches, feeding habits, and related aspects of the mollusk species identified at Llonín.

Notes on the figures and Supplementary Materials

- Fig. 3: I would suggest adding the number of pieces for each graph

The figure has been subjected to several changes, including colors and the addition of some extra graphics regarding manufacturing techniques. We have also included the number of pieces for each graph as suggested, and we thank you for the suggestion, it's true that, given the high number of species, especially for the Solutrean, it was difficult to correctly interpret the graphics.

- S1 dataset: I suppose the code of the column is indicated in the fig. 4 caption, but I think it would be easier for the reader to have a second sheet with a caption indicating what each abbreviation means

The requested modifications have been implemented

- As mentioned above, I believe you are referring to S2 several times, when in fact you are referring to another document.

The requested modifications have been implemented

Suggested bibliography on experiments aiming to identify ornamental composition:

d'Errico F., Jardón Giner P., Soler Mayor B. (1993) – Critères à base expérimentale pour l'étude des perforations naturelles et intentionnelles sur coquillages, *in* P. Anderson, S. Beyries, M. Otte, et H. Plisson (dir.), *Traces et fonctions : les gestes retrouvés. Actes du colloque international de Liège, 8-10 décembre*, , Liège, ERAUL : Études et Recherches Archéologique de l'Université de Liège p.243-254.

Hoareau L., Beyries S. (2022) – Insights into use-wear development on shell beads through macro- and microanalysis of experimental ornaments, *in* M. Mărgărit et A. Boroneanţ (dir.), *Recreating artefacts and ancient skills : from experiment to interpretation*, , Târgovişte, Cetatea de scaun p.183-200.

Langley M.C., O'Connor S. (2016) – An Enduring Shell Artefact Tradition from Timor-Leste: *Oliva* Bead Production from the Pleistocene to Late Holocene at Jerimalai, Lene Hara and Matja Kuru 1 and 2, *PLOS ONE*, 11, 8, p. 1-25.

Mărgărit M. (2016) – Testing the endurance of prehistoric adornments: Raw materials from the aquatic environment, Journal of Archaeological Science, 70, p. 66-81.

Schechter H.C. (2023) – Experimenting with Levantine Neolithic shell-bead production and use – A low magnification perspective, *Journal of Archaeological Science: Reports*, 52, p. 104231.

Torres H.R., Groman-Yaroslavski I., Weinstein-Evron M., Yeshurun R. (2020) – A micro-wear analysis of Natufian gazelle phalanx beads from el-Wad Terrace, Mount Carmel, Israel, Journal of Archaeological Science: Reports, 31, p. 1-11.

Osipowicz G., Piličiauskienė G., Justyna O., Piličiauskas G. (2020) – An occasional ornament, part of clothes or just a gift for ancestors? The results of traceological studies of teeth pendants from the Subneolithic sites in Šventoji, Lithuania, *Journal of Archaeological Science: Reports*, 29, 2020, p. 102-130.

Mouclier P., Schiefenhövel W., Vanhaeren M. (2025) – A Study of a Traditional *Nassarius* Shell Bead Headband from the Mek Culture, Highlands of Western New Guinea: Ethnographic and Archaeological Perspectives, *Ethnoarchaeology*, p. 1-38.
