# Supplementary material for: Identity and mobility through personal ornaments in Upper Paleolithic cantabrian hunter-gatherer societies: Insights from Llonín cave (Asturias, Spain)
Source: PLoS One. 2026 Jun 8;21(6):e0351170. doi: 10.1371/journal.pone.0351170 (PMC13245794; doi:10.1371/journal.pone.0351170)
Supplement: Attachment — Submitted filename: Review_PONE-D-25-62247.docx [file pone.0351170.s010.docx]

Review PONE-D-25-62247

This is a very interesting paper that documents the diachronic evolution of ornamental practices as well as their social role and the functional aspects of the site. The conclusions are very interesting and integrate the information from the symbolic sphere into the overall functioning of late Paleolithic societies, which gives this study significant scope. The supplementary materials are particularly valuable and provide access to all the raw data.

I recommend publishing this article with moderate revisions.

I find this work very rigorously done, but I have a few suggestions, particularly about the traceological section, which I think would benefit from a few clarifications. There are also some questions out of curiosity, the answers to which the authors can include in their paper if they wish to provide details.

**Methodes**

**Taxonomic and biometric analyses**

- L. 128: you indicate the use of “specialized literature”, but there is no bibliography associated. Could you indicate the references?

**Morphometric and use-wear analysis**

- L. 180: you cite S2 Table following a statement about the experimental collection, but the table lists data on archaeological red deer canines.

**Results**

**Taxonomic analysis**

- L. 236-238: Can you specify which teeth were found for each species (canine/incisor/molar, etc.)?

**Taphonomic analysis**

- L. 266: same problem as in line 180, S2 table does not provide taphonomic information as it only lists data on archaeological red deer canines (I do not see a “taphonomic alteration” column).
- I think that some aspects discussed in the section on “taphonomic analysis” could be integrated differently:
  - L. 268-269: I think this is a matter of debate, and not everyone agrees. I completely understand if you disagree. However, I don't know if thanatocenosis alterations should be mixed with alterations related to diagenesis (in the context of an archeological study, which aims to understand the role of ornaments in past societies, it is obviously different in paleontology), since they do not provide information about the same stages in the life of objects. I'll leave it up to you to decide whether you want to include this or not.
  - L. 275-277: I believe that extraction marks on teeth should not be included in the category of taphonomy but rather in the manufacturing/acquisition process, as they are part of the technical actions that humans have used to transform animal material into symbolic objects.
- L. 271-275: Are you sure that this is sediment-related impregnation? In the photographs (which are very good quality!) that you have provided, iron oxides appear to be quite clearly under the sediment and encrustations. This may not be the case for all the pieces, but this might be evidence of deliberate coloration of some of the shells at the site. I am thinking in particular of photograph P, on which iron oxides are relatively concentrated in the natural depression of the shells, with encrustations (that does not contain oxydes) that partially cover the deposits. For my part, I would use these arguments to assert that certain elements have been colored, but if there are arguments against this interpretation, it could be added to the text.
- L. 277-279: It would be interesting to explain the characteristics that led you to conclude that this was accidental exposure (uneven color? Micro-flaking? Uneven cracked appearance? etc.). The item you show in fig. 6G appears to have changed color to a uniform shiny black, which I think could mean it is possible that it was deliberately heated to change color.

**Use-wear analysis**

- L. 306-308: Are there signs of wear on these naturally perforated pieces? This would prove that paleolithic humans did indeed take advantage of these natural perforations. It would also be interesting to share the criteria you used to determine that the perforations were of natural origin (based on d’Errico et al., 1993? ).

**Production of ornaments**

- L. 321: I do not understand technically how abrasion can be used to widen the medullary canal of the vertebra. The lack of photographs and of description of the traces makes it difficult to understand the technical gesture.
- L. 344: I'm not sure I understand what technical gesture you mean by “trimming.” Could you elaborate?
- Fig 7: There is an error in the caption; figure L is not the tooth of *Capra pyrenaica* (I assume it is M).
- L. 371-382: I think this description of your identification criteria should appear at the beginning of the section on perforation techniques so that readers know on what grounds the techniques are identified in the rest of the section. However, I believe that morphology alone is not sufficient to determine the techniques. I suggest that you include the section of perforations, striations, and removals as presented in (Tátá et al., 2014), and explain at the beginning of the section on “production of ornaments” which sets of criteria correspond to which techniques.
- General question: given that the object are worn, as you mention later in your article, does this not interfere with the observation of manufacture traces? Could you add a sentence about this potential obstacle?

**Use and suspension systems of personal ornaments**

- L. 389: I think that the attenuation and erasure of technological traces is an identification criterion that works for all types of materials, as suggested by S. Bonnardin or M. Mărgărit (Bonnardin, 2008 ; Mărgărit, 2016)
- L. 405: When you say “no difference in use,” does that mean that the type of traces and their organisation on the different material is identical, or simply that all types of materials show use-wear?
- L. 407-409: Could you describe what led you to this conclusion? In other words, could you describe the traces in question in detail, explaining how they are more or less developed depending on the item considered?
- L. 411: The bibliography you present on this matter is limited. Even though the literature has greatly expanded in recent years, I think it is possible and preferable to cite a more comprehensive list of references (suggestions at the end of this document)
- L. 417-418: This may be a misinterpretation on my part, but it seems in this sentence that you are suggesting that *Antalis* beads can only be arranged in one type of composition. I agree that there is only one way to thread a string through a tubular bead such as *Antalis*, however there are many ways to integrate this into various compositions such as sawing as suggested in (Vanhaeren et D’Errico, 2001) or individually tied as tested in (Hoareau et Beyries, 2022)
- L. 422: Why buttons? Why not just a simple knot or sawing, with only one side of the tooth against the wearer? I don't know of any experiments involving buttons, but I would assume that the friction of the element that closes the button would affect the entire surface, not only one side.
- Fig. 10: Some of the photographs do not allow the reader to follow the reasoning leading from the traces to the reconstruction hypothesis. May I suggest at least including supplementary materials showing the array of traces that lead to each of the reconstructions? I am thinking in particular of hypotheses D, E, and F, which must be based on a set of relatively complex evidence that are difficult to summarize in one photograph. Furthermore, are these hypotheses based on comparisons with the experimental collection? Can you show photographs of the experimental traces?
- General: Are there suspension modes linked to a particular type of shell? How many elements correspond to each suspension mode? What is the array of evidence that lead to one interpretation rather than another?

**Decorated elements**

- L. 442: Could the depth and length of the incisions be distinctive criteria?
  - Fig. 11 and l. 469: About the incisions on *Chlamys islandica* : it's up to you if you want to argue further that the incisions were not random, but I don't think the organization is very clear. Have you observed the bottom of the incisions? Secondary striations could indicate the use of a stone tool and help settle the question. If you don't want to expand on this part, I don't think it's a problem, as the photographic documentation is very good and should be sufficient for readers to develop their own interpretation on whether the arrangement is random or not

**Discussion**

- L. 499: Here too, the dataset you refer to is that of archaeological deer teeth.
- L. 558-560: I disagree somewhat, as some of the visual arrangements can be approached through traceology (through experiments + ethnography, as in the bibliography at the end of the document). I believe that more conclusive demonstrations could be achieved by conducting specific experiments based on the type of traces observed in the archeological assemblage (is that a perspective for the study of this site ?). On the other hand, I completely agree that it is impossible to know which part of the body was adorned without a well-preserved funerary context.
- L. 580-582: Considering the possible acquisition of objects through exchange, would this imply that objects circulated in their raw form, not perforated? Does this indicate a segmentation of the production chain that only existed during the Solutrean period?
- L. 590: Is the type of functional use-wear the same? This could be decisive in identifying them as coming from a single composition. It also makes me think that, if possible, a dataset detailing the types of wear and their distribution on the pieces would give a precise idea of the compositions.
- L. 593: To support either hypothesis, is there any data on lithic raw materials? I don’t think you develop the topic of lithic material circulation for the badegoulian in the « cultural network and mobility » section
- L. 595: I think it's possible to expand on this a little further. I understand from this sentence that climatic conditions were theoretically just as difficult in the late Solutrean and Badegoulian periods, so there would be no environmental reasons to explain this difference. But there might be some evolutions at the local scale? Was there a change in the local landscape between the late Solutrean and Badegourian periods? Depending on whether it is possible to identify specific environmental obstacles, this raises questions about cultural choices and the organization of networks: was there a reduction in the network of material circulation linked to adornment, but was the same true for other areas such as lithics?
- L. 636 – 638: Is this stable throughout the entire chronology that interests you here?
- L. 657: If you have any data on species evolution available, it would be interesting to cite it here, or even to discuss the contemporaneity of the evolution of species present on the coastline vs. those used by paleolithic groups.

**Notes on the figures and Supplementary Materials**

- Fig. 3: I would suggest adding the number of pieces for each graph
- S1 dataset: I suppose the code of the column is indicated in the fig. 4 caption, but I think it would be easier for the reader to have a second sheet with a caption indicating what each abbreviation means
- As mentioned above, I believe you are referring to S2 several times, when in fact you are referring to another document.

**Suggested bibliography on experiments aiming to identify ornamental composition:**

d’Errico F., Jardón Giner P., Soler Mayor B. (1993) ‒ Critères à base expérimentale pour l’étude des perforations naturelles et intentionnelles sur coquillages, *in* P. Anderson, S. Beyries, M. Otte, et H. Plisson (dir.), *Traces et fonctions : les gestes retrouvés. Actes du colloque international de Liège, 8-10 décembre*, , Liège, ERAUL : Études et Recherches Archéologique de l’Université de Liège  p.243‑254.

Hoareau L., Beyries S. (2022) ‒ Insights into use-wear development on shell beads through macro- and microanalysis of experimental ornaments, *in* M. Mărgărit et A. Boroneanţ (dir.), *Recreating artefacts and ancient skills : from experiment to interpretation*, , Târgovişte, Cetatea de scaun  p.183‑200.

Langley M.C., O’Connor S. (2016) ‒ An Enduring Shell Artefact Tradition from Timor-Leste: *Oliva* Bead Production from the Pleistocene to Late Holocene at Jerimalai, Lene Hara and Matja Kuru 1 and 2, *PLOS ONE*, 11, 8, p. 1‑25.

Mărgărit M. (2016) ‒ Testing the endurance of prehistoric adornments: Raw materials from the aquatic environment, Journal of Archaeological Science, 70, p. 66‑81.

Osipowicz G., Piličiauskienė G., Justyna O., Piličiauskas G. (2020) ‒ An occasional ornament, part of clothes or just a gift for ancestors? The results of traceological studies of teeth pendants from the Subneolithic sites in Šventoji, Lithuania, *Journal of Archaeological Science: Reports*, 29, 2020, p. 102‑130.

Schechter H.C. (2023) ‒ Experimenting with Levantine Neolithic shell-bead production and use – A low magnification perspective, *Journal of Archaeological Science: Reports*, 52, p. 104231.

Torres H.R., Groman-Yaroslavski I., Weinstein-Evron M., Yeshurun R. (2020) ‒ A micro-wear analysis of Natufian gazelle phalanx beads from el-Wad Terrace, Mount Carmel, Israel, Journal of Archaeological Science: Reports, 31, p. 1‑11.

Mouclier P., Schiefenhövel W., Vanhaeren M. (2025) ‒ A Study of a Traditional *Nassarius* Shell Bead Headband from the Mek Culture, Highlands of Western New Guinea: Ethnographic and Archaeological Perspectives, *Ethnoarchaeology*, p. 1‑38.
