# Supplementary material for: Identity and mobility through personal ornaments in Upper Paleolithic cantabrian hunter-gatherer societies: Insights from Llonín cave (Asturias, Spain)
Source: PLoS One. 2026 Jun 8;21(6):e0351170. doi: 10.1371/journal.pone.0351170 (PMC13245794; doi:10.1371/journal.pone.0351170)
Supplement: Peer review history

# Peer review: Decision Letter 0

**DOI:** 10.1371/journal.pone.0351170.r001

**Authors:** Marco Peresani

23 Feb 2026

PONE-D-25-62247

Identity and Mobility through Personal Ornaments in Upper Paleolithic Cantabrian Hunter-Gatherer Societies: insights from Llonín cave (Asturias, Spain)

PLOS One

Dear Dr. Pérez-García de los Salmones,

Thank you for submitting your manuscript to PLOS ONE. After careful consideration, we feel that it has merit but does not fully meet PLOS ONE’s publication criteria as it currently stands.

===================================

Both Reviewers have raised several critical points on your study despite they diverge from recommendation. Particularly R.1, recommends rejection although in the meantime recognizes the valuable contribution of your work in shedding light on unpublished pendants and beads recovered in one of the most important Palaeolithic sites in Cantabria. The main raised points cast doubts on the stratigraphic position of some areas of Llonín cave where the items came from, the methodology used, the way the results are presented and your conclusions which need improvements. Relationship between the objects, the volume of excavated sediment, the archaeological level, and the sector of the cave must be investigated to assess the degree of possible significant differences between the areas. Eventually, you are requested to arrive to a selection of those sectors expressing the greatest stratigraphic integrity like Galería, from where the highest number of pendants have been documented. In addition to this pivotal aspect, R.1 raises many points to be managed in all sections of the manuscript, with no exclusion for tables and figures. About R.2, request is for minor revisions despite the long list of rebuttals, dealing mostly with the taphonomy. R.2’s reasons are presented in the report attached to this email.

To summarize, given the archaeological relevance of Llonín cave and the invaluable work carried out by your team, my final though is to request major revisions at place of rejection of the manuscript.

Therefore, we invite you to submit a thoroughly revised version of the manuscript that addresses the points raised during the review process.

===================================

Please submit your revised manuscript by Apr 09 2026 11:59PM. If you will need more time than this to complete your revisions, please reply to this message or contact the journal office at plosone@plos.org. When you're ready to submit your revision, log on to https://www.editorialmanager.com/pone/ and select the 'Submissions Needing Revision' folder to locate your manuscript file.

Please include the following items when submitting your revised manuscript:

- A letter that responds to each point raised by the academic editor and reviewer(s). You should upload this letter as a separate file labeled 'Response to Reviewers'.
- A marked-up copy of your manuscript that highlights changes made to the original version. You should upload this as a separate file labeled 'Revised Manuscript with Track Changes'.
- An unmarked version of your revised paper without tracked changes. You should upload this as a separate file labeled 'Manuscript'.

If you would like to make changes to your financial disclosure, please include your updated statement in your cover letter. Guidelines for resubmitting your figure files are available below the reviewer comments at the end of this letter.

If applicable, we recommend that you deposit your laboratory protocols in protocols.io to enhance the reproducibility of your results. Protocols.io assigns your protocol its own identifier (DOI) so that it can be cited independently in the future. For instructions see: https://journals.plos.org/plosone/s/submission-guidelines#loc-laboratory-protocols. Additionally, PLOS ONE offers an option for publishing peer-reviewed Lab Protocol articles, which describe protocols hosted on protocols.io. Read more information on sharing protocols at https://plos.org/protocols?utm_medium=editorial-email&utm_source=authorletters&utm_campaign=protocols.

We look forward to receiving your revised manuscript.

Kind regards,

Marco Peresani

Academic Editor

PLOS One

Journal Requirements:

When submitting your revision, we need you to address these additional requirements.

1. Please ensure that your manuscript meets PLOS ONE's style requirements, including those for file naming. The PLOS ONE style templates can be found at

https://journals.plos.org/plosone/s/file?id=wjVg/PLOSOne_formatting_sample_main_body.pdf and https://journals.plos.org/plosone/s/file?id=ba62/PLOSOne_formatting_sample_title_authors_affiliations.pdf

2. In your manuscript, please provide additional information regarding the specimens used in your study. Ensure that you have reported human remain specimen numbers and complete repository information, including museum name and geographic location.

If permits were required, please ensure that you have provided details for all permits that were obtained, including the full name of the issuing authority, and add the following statement:

'All necessary permits were obtained for the described study, which complied with all relevant regulations.'

If no permits were required, please include the following statement:

'No permits were required for the described study, which complied with all relevant regulations.'

For more information on PLOS One's requirements for paleontology and archeology research, see https://journals.plos.org/plosone/s/submission-guidelines#loc-paleontology-and-archaeology-research.

3. We note that the grant information you provided in the ‘Funding Information’ and ‘Financial Disclosure’ sections do not match.

When you resubmit, please ensure that you provide the correct grant numbers for the awards you received for your study in the ‘Funding Information’ section.

4. Please amend either the title on the online submission form (via Edit Submission) or the title in the manuscript so that they are identical.

5. We note that Figure 1, S1, S2 in your submission contain [map/satellite] images which may be copyrighted. All PLOS content is published under the Creative Commons Attribution License (CC BY 4.0), which means that the manuscript, images, and Supporting Information files will be freely available online, and any third party is permitted to access, download, copy, distribute, and use these materials in any way, even commercially, with proper attribution. For these reasons, we cannot publish previously copyrighted maps or satellite images created using proprietary data, such as Google software (Google Maps, Street View, and Earth). For more information, see our copyright guidelines: http://journals.plos.org/plosone/s/licenses-and-copyright.

We require you to either (1) present written permission from the copyright holder to publish these figures specifically under the CC BY 4.0 license, or (2) remove the figures from your submission:

a. You may seek permission from the original copyright holder of Figure 1, S1, S2 to publish the content specifically under the CC BY 4.0 license.

We recommend that you contact the original copyright holder with the Content Permission Form (http://journals.plos.org/plosone/s/file?id=7c09/content-permission-form.pdf) and the following text:

“I request permission for the open-access journal PLOS ONE to publish XXX under the Creative Commons Attribution License (CCAL) CC BY 4.0 (http://creativecommons.org/licenses/by/4.0/). Please be aware that this license allows unrestricted use and distribution, even commercially, by third parties. Please reply and provide explicit written permission to publish XXX under a CC BY license and complete the attached form.”

Please upload the completed Content Permission Form or other proof of granted permissions as an "Other" file with your submission.

In the figure caption of the copyrighted figure, please include the following text: “Reprinted from [ref] under a CC BY license, with permission from [name of publisher], original copyright [original copyright year].”

b. If you are unable to obtain permission from the original copyright holder to publish these figures under the CC BY 4.0 license or if the copyright holder’s requirements are incompatible with the CC BY 4.0 license, please either i) remove the figure or ii) supply a replacement figure that complies with the CC BY 4.0 license. Please check copyright information on all replacement figures and update the figure caption with source information. If applicable, please specify in the figure caption text when a figure is similar but not identical to the original image and is therefore for illustrative purposes only.

The following resources for replacing copyrighted map figures may be helpful:

USGS National Map Viewer (public domain): http://viewer.nationalmap.gov/viewer/

The Gateway to Astronaut Photography of Earth (public domain): http://eol.jsc.nasa.gov/sseop/clickmap/

Maps at the CIA (public domain): https://www.cia.gov/library/publications/the-world-factbook/index.html and https://www.cia.gov/library/publications/cia-maps-publications/index.html

NASA Earth Observatory (public domain): http://earthobservatory.nasa.gov/

Landsat: http://landsat.visibleearth.nasa.gov/

USGS EROS (Earth Resources Observatory and Science (EROS) Center) (public domain): http://eros.usgs.gov/#

Natural Earth (public domain): http://www.naturalearthdata.com/

6. We note Figure(s) 1, 2, 6, 7, 8, 9, 10, 11, S1, S2, S3, S4, S5, S6, S8, S9, S10 in your submission contain copyrighted images. All PLOS content is published under the Creative Commons Attribution License (CC BY 4.0), which means that the manuscript, images, and Supporting Information files will be freely available online, and any third party is permitted to access, download, copy, distribute, and use these materials in any way, even commercially, with proper attribution. For more information, see our copyright guidelines: http://journals.plos.org/plosone/s/licenses-and-copyright.

We require you to either (1) present written permission from the copyright holder to publish these figures specifically under the CC BY 4.0 license, or (2) remove the figures from your submission:

a. You may seek permission from the original copyright holder of Figure(s) 1, 2, 6, 7, 8, 9, 10, 11, S1, S2, S3, S4, S5, S6, S8, S9, S10 to publish the content specifically under the CC BY 4.0 license.

We recommend that you contact the original copyright holder with the Content Permission Form (http://journals.plos.org/plosone/s/file?id=7c09/content-permission-form.pdf) and the following text:

“I request permission for the open-access journal PLOS ONE to publish XXX under the Creative Commons Attribution License (CCAL) CC BY 4.0 (http://creativecommons.org/licenses/by/4.0/). Please be aware that this license allows unrestricted use and distribution, even commercially, by third parties. Please reply and provide explicit written permission to publish XXX under a CC BY license and complete the attached form.”

Please upload the completed Content Permission Form or other proof of granted permissions as an "Other" file with your submission.

In the figure caption of the copyrighted figure, please include the following text: “Reprinted from [ref] under a CC BY license, with permission from [name of publisher], original copyright [original copyright year].”

b. If you are unable to obtain permission from the original copyright holder to publish these figures under the CC BY 4.0 license or if the copyright holder’s requirements are incompatible with the CC BY 4.0 license, please either i) remove the figure or ii) supply a replacement figure that complies with the CC BY 4.0 license. Please check copyright information on all replacement figures and update the figure caption with source information. If applicable, please specify in the figure caption text when a figure is similar but not identical to the original image and is therefore for illustrative purposes only.

7. We note that there is identifying data in the Supporting Information file <S1_Dataset.xlsx, S2_Dataset.xlsx, S3_Dataset.xlsx, S5_Dataset.xlsx, S1_Text and S1-S2_tables.pdf, S1-S7_Figs.pdf, S8_Fig.pdf, S9-S10 Figs.odf >. Due to the inclusion of these potentially identifying data, we have removed this file from your file inventory. Prior to sharing human research participant data, authors should consult with an ethics committee to ensure data are shared in accordance with participant consent and all applicable local laws.

Data sharing should never compromise participant privacy. It is therefore not appropriate to publicly share personally identifiable data on human research participants. The following are examples of data that should not be shared:

-Name, initials, physical address

-Ages more specific than whole numbers

-Internet protocol (IP) address

-Specific dates (birth dates, death dates, examination dates, etc.)

-Contact information such as phone number or email address

-Location data

-ID numbers that seem specific (long numbers, include initials, titled “Hospital ID”) rather than random (small numbers in numerical order)

Data that are not directly identifying may also be inappropriate to share, as in combination they can become identifying. For example, data collected from a small group of participants, vulnerable populations, or private groups should not be shared if they involve indirect identifiers (such as sex, ethnicity, location, etc.) that may risk the identification of study participants.

Additional guidance on preparing raw data for publication can be found in our Data Policy (https://journals.plos.org/plosone/s/data-availability#loc-human-research-participant-data-and-other-sensitive-data) and in the following article: http://www.bmj.com/content/340/bmj.c181.long.

Please remove or anonymize all personal information (<specific identifying information in file to be removed>), ensure that the data shared are in accordance with participant consent, and re-upload a fully anonymized data set. Please note that spreadsheet columns with personal information must be removed and not hidden as all hidden columns will appear in the published file.

8. If the reviewer comments include a recommendation to cite specific previously published works, please review and evaluate these publications to determine whether they are relevant and should be cited. There is no requirement to cite these works unless the editor has indicated otherwise.

[Note: HTML markup is below. Please do not edit.]

Reviewers' comments:

Reviewer's Responses to Questions

**Comments to the Author**

1. Is the manuscript technically sound, and do the data support the conclusions?

The manuscript must describe a technically sound piece of scientific research with data that supports the conclusions. Experiments must have been conducted rigorously, with appropriate controls, replication, and sample sizes. The conclusions must be drawn appropriately based on the data presented.

Reviewer #1: Partly

Reviewer #2: Yes

**********

2. Has the statistical analysis been performed appropriately and rigorously?

Reviewer #1: I Don't Know

Reviewer #2: Yes

**********

3. Have the authors made all data underlying the findings in their manuscript fully available?

The PLOS Data policy requires authors to make all data underlying the findings described in their manuscript fully available without restriction, with rare exception (please refer to the Data Availability Statement in the manuscript PDF file). The data should be provided as part of the manuscript or its supporting information, or deposited to a public repository. For example, in addition to summary statistics, the data points behind means, medians and variance measures should be available. If there are restrictions on publicly sharing data—e.g. participant privacy or use of data from a third party—those must be specified.

Reviewer #1: Yes

Reviewer #2: Yes

**********

4. Is the manuscript presented in an intelligible fashion and written in standard English?

PLOS ONE does not copyedit accepted manuscripts, so the language in submitted articles must be clear, correct, and unambiguous. Any typographical or grammatical errors should be corrected at revision, so please note any specific errors here.

Reviewer #1: Yes

Reviewer #2: Yes

**********

5. Review Comments to the Author

Please use the space provided to explain your answers to the questions above. You may also include additional comments for the author, including concerns about dual publication, research ethics, or publication ethics. (Please upload your review as an attachment if it exceeds 20,000 characters)

Reviewer #1: The Cantabrian region is one of the European regions where the most information can be obtained about the symbolic behavior of hunter-gatherer groups during Prehistory, given the enormous number of sites and archaeological studies that have been carried out there over the last 25 years. In particular, in the case of beads and pendants, and following an initial study of the archaeological evidence discovered up to the beginning of this century by Alvares-Fernandes (2006), specific studies have been carried out in the Cantabrian region on new sites (Praile Aitz II, Toral, Mazo, etc.) in recent years, which has made it possible to clarify the role of these objects.

This article, submitted for review to the journal PloSOne, presents a comprehensive study of the pendants documented in excavations carried out in different areas of the Llonin cave (Asturias), originating from interventions carried out by J. Fortea at the end of the last century. Occupations have been documented from the Mousterian to the Bronze Age, making it one of the sites with the most continuous occupation over time, first by Neanderthals and then by H. sapiens. This is a virtually unpublished study (reviewing Álvares 2006, the study of three of the objects also included here is cited). Apart from the decorative objects (207, one of the most abundant recorded at a site in the north of the Iberian Peninsula), other evidence (62) has been included that has been interpreted as raw materials for the manufacture of decorative objects.

INTRODUCTION

- Use “Middle Stone Age-MSA” instead of “Middle Paleolithic” when referring to the earliest contexts where shell beads have been documented in Africa

- It is noted that the earliest evidence of objects dates from the transition from the Middle to the Upper Paleolithic in Europe. Indicate where and why the authors consider that these objects should really be interpreted as ornaments.

- There is a lack of bibliography on the earliest evidence of ornamental objects in Central and Eastern Europe. Include, for example, the recent article by d'Errico et al. 2025 (PlosONE).

- There is a lack of traceological and experimental studies in the Iberian Peninsula (e.g., Avezuela et al. 2010).

MATERIALS & METHODS

- Place the Llonín cave in the Cantabrian context, in greater detail.

- Indicate why Llonín is considered a residential complex during the Upper Solutrean period, much more specialized than the Badegoulian occupation. Include publications that explain this point.

- This part of the article includes research results: number of objects, distribution by type and period, etc. This information should be included in the results section, not before the methodology, as it currently appears.

- Aspects related to the methodology for recovering objects should be indicated (found during excavation, during sediment sorting, types of sieves used to recover the remains, etc.).

- Indicate the area excavated in each zone of the cave and whether there have been differences between the distinct levels excavated throughout the sequence.

- Indicate whether spatial studies have been carried out, which may, for example, indicate whether there are concentrations of ornaments, place of manufacture, etc.

- Taxonomic & Biometric analyses again include research results. This is not the place for them. Information relating to holes is not included. There is no information on the classification of marine and terrestrial species and their habitat areas.

- Taphonomic analysis: in the case of shells, the references cited refer to marine mollusks, not those of riverine origin. There is no data or methodology on how the holes are made, or what techniques are used (abrasion, etc.). The methodology used for alterations is very limited. It needs to be more specific (calcium carbonate precipitation, thermal alteration, etc.). The order should be: alterations prior to the animal's death, anthropic alterations (drilling, application of ochre, traces of use, etc.) and post-depositional alterations.

- Morphometric and use wear analysis: A series of bibliographic references on manufacturing and suspension methods are provided. However, some of these references do not explicitly address these topics. It is necessary to be able to correlate manufacturing techniques, hole sizes, and signs of use.

RESULTS

Teeth:

- It is necessary to indicate what archaeofaunal information is available for each level and area, to see if, for example, the most hunted species corresponds to the species most used for bead creation. The MNI is established based on red deer canines. Does this coincide with the most hunted species? This will allow for a greater focus on strategies for obtaining raw materials from teeth, and thus the conclusions regarding the mobility of groups based on these materials will be more precise. Are there teeth from other species without perforations?

- There is a lack of experimentation on red deer canines that could determine how the surfaces to be perforated were prepared and how the perforations were made, and how this process and use have erased or not erased these technological traces.

- A better distinction needs to be made between preparation marks and other types of marks (jaw extraction, decoration). For example, in one of the specimens, it is indicated that the incision near the enamel is due to the need to frame the surface to be perforated, when in fact it is an incision to extract the tooth from the jaw.

- There is doubt that CA.X.85 and CA.XII.3 are atrophic deer canines; this information needs to be reviewed. Both are very similar and have the same decoration. They come from different levels, with different chronological criteria. Given that they come from an area where the stratigraphic entity is not guaranteed, is it likely that they belong to a single occupation?

- Traces of use should be indicated on all parts of the tooth that preserve them, perhaps by drawing them as M. Vanhaeren points out in his works (some of which are cited by the authors). The same should be indicated for ochre. In this way, traces of use will be correctly interpreted and the information provided in the article will be more accurate.

Shells:

- It is necessary to indicate what archaeofaunal information is available for each level and area. The existence of Littorina littorea and Patella sp. is shown. These species have nutritional value. Although some are perforated, there are fragments that are not perforated. It should be indicated whether there are remains of these species collected as food in different areas of the cave. This will provide more insight into strategies for gathering raw materials from shells and thus allow for more precise conclusions about the mobility of groups based on these materials. For example, strategies are not the same if the shells are collected on beaches (already dead) or on rocks (alive).

- A better distinction needs to be made between preparation marks and other types of marks (how can abrasion caused by sea sand be distinguished from that caused by human activity?).

- There are doubts about the identification of some of the proposed species. Although the reviewer only has access to the photos presented in the article, it is clear that errors have been made in the identification of some of the shells: This is the case with CA. XI.52 and CA.UD.2, which are clearly not Nucella lapillus, or CA.XI.54, which, given that the external surface with the reticulate decoration of the bivalve Ruditapes decussatus is not preserved, raises doubts about its classification at the species level. Correct taxonomic identification is essential for the study of shell ornaments.

- The influence of post-depositional alterations on aspects such as the preparation of the surfaces to be perforated, the making of perforations, and the preservation of traces of use has not been properly assessed. This is the case with calcium carbonate precipitation, which prevents or hinders its study, as does decalcification (see, for example, shells CA.IX.1 and CA.X.86). Shell VE.IV.1 retains sediment remains inside, making it impossible to document how it was perforated. On other occasions (in shells that lack detailed photos of the holes), there is doubt as to whether the interpretation of the anthropic traces has been carried out correctly, for example, whether the fractures in the holes are due to prolonged use (e.g., Pecten sp. valve). It would be necessary to incorporate detailed photos of all the holes.

- Traces of use and ochre should be indicated on all parts of perforated shells (see comment on teeth).

- Shells without traces of anthropic manipulation are included. How can we be sure that they are not fragments of shells collected as food rather than raw material for making ornaments, or broken ornaments that do not retain the hole?

- In the case of small Littorina obtusata/Littorina fabalis, could there be any other interpretation than that they are raw material for the manufacture of artifacts? In this regard, it is worth reviewing the article on Praile Aitz by F. d'Errico and his team, cited in the article.

DECORATED ELEMENTS

- Point out how the author differentiates between decorative marks and technological marks. This aspect should have been indicated in the methodology.

DISCUSSION & CONCLUSIONS

- The discussion, considering the comments made on the results, must be nuanced and clarified.

- In the case of the fossil bead, indicate where the geological formations are located in Asturias and closest to Llonín.

- As indicated, this article does not provide any information on how the holes in the ornaments were made, whether there are variations between species or periods.

- It is surprising that the documented traces of use are only found in the holes. If no traces of use have been documented in other parts of the supports, the reason for this should be indicated.

- No convincing information is provided as to why pendants are produced in Llonín (work areas, fragmented objects in the process of being made, etc.).

- Specify the relationship between recovered objects, volume of excavated sediment, archaeological level, and area of the cave. This will allow us to observe whether there are significant differences between the different areas of the cave that have been excavated and between different periods.

- There is mention of an event involving the gathering or exchange of ornaments in the cave with groups that came from outside. This term, coined by M. Conkey, needs further explanation (the author is not cited in the paper).

- It is noted that some of the archaeological material is not in its original position and that, due to sedimentological dynamics, there are doubts about the attribution of certain materials. However, these materials have been included in the analysis and interpretation.

- The mobility inferred from the objects should be better specified. This information should be cross-referenced in detail with other types of information studied in Llonín (e.g., lithic raw materials).

TABLES

- Table 1. This should be redone, incorporating the totals for objects in the different periods. Details should also be included on the type of support used by taxon, whether they are shells (specifying gastropods, bivalves, and scaphopods), teeth, or bones belonging to the different taxa indicated. Among the taxa indicated, it has been specified that, in the case of the horse, the species has not been identified as it is not certain (despite the fact that in the Cantabrian region, the species hunted, as cited by archaeozoologists, is Equus caballus); on the contrary, the species Salmo salar has been specified, when the aforementioned researchers indicate that it is difficult to determine specimens of the Salmonidae family exclusively from their vertebrae. It is indicated that this species is riverine but also inhabits the sea. The identification of perforated teeth of Vulpex vulpes and not Alopex lagopus is also noted, although the latter species has been documented by Altuna (2004) in the north of the Iberian Peninsula in the Paleolithic period. In this case, has it been possible to differentiate the first taxon from the second? Why have specimens of the Glycymeriidae family been considered as Glycymeris glycymeris, if 1) the shells are decalcified and show no coloration, and 2) there are different species of this family in the Cantabrian/Mediterranean region to which the shells documented in Llonín may belong?

FIGURES

- Figure 1. The upper part includes sites where pendants have been documented. The relevant bibliography for each of them must be indicated. The lower part shows the stratigraphy of each of the areas excavated in the cave. This part can be included in “Supplementary Information.”

- Each piece studied must be accompanied by a photo showing the hole in detail. This information must be also included in the SI.

REFERENCES

I consider the bibliographic citations used in this article to be appropriate (although specific aspects of these publications have been previously pointed out in the different parts of the article that have been revised). However, there are errors in the citation of some of the references. Authors who did not participate in the writing of these articles have been included; e.g., Jones et al. 2021-QSR; d'Errico et al. 2015-Archaeometry; Martín-Jarque et al. 2023- L'Anthropològie; Corchón et al. 2008 L'Anthropològie). A thorough review is required to check for errors of this type in this part of the article.

SUPPLEMENTARY INFORMATION

S1_Dataset

The legend does not include all the information needed for it to be correctly reviewed and interpreted. There is no fundamental data recorded for each of the pieces (basic dimensions, dimensions of the perforations, dimensions of the root, in the case of teeth, etc.). This Excel file needs to be much more detailed.

S1_Dataset

This refers exclusively to teeth. The meaning of “UD” must be indicated, as well as other data, such as the maximum and minimum diameter of the perforation, the techniques used to create the hole, and the taphonomic alterations affecting each of the teeth (concretion, ochre, thermal alteration, etc.). It is essential that there be a reference to the photos presented (details of the perforation, etc.), indicated in the figures in the text.

S2_Dataset

This table must be merged with S1_Dataset, which refers to teeth. In the case of other raw materials, they must be separated into other Excel tables (shells, etc.). The headings must be developed (e.g., EPrf. Technique_P1), as well as the techniques (AB, IM, RT, etc.), the location of the holes (e.g., E1), etc. Traces of use must be specified (part of the perforation, root or other parts in the case of teeth, other parts of shells, etc.). The part of the shell where ochre appears must be indicated. In this case, and in terms of use, it is appropriate to make drawings of different views of each piece and indicate with colors the areas of use and the presence of ochre.

S3_Dataset

This refers to the comparison of measurements of archaeological shells that appear in abundance in the Llonín cave with measurements of current specimens. This information is relevant.

S4_Dataset

It refers to the measurements of the different objects. These must be included with the rest of the information that must be indicated on each of the Excel sheets created for each type.

Text S1 & Table S1

This text is essential for understanding the archaeological interventions carried out in different areas of the cave. As indicated, “Gallery” is the area where “provides the best-preserved spatial distribution of archaeological elements at Llonín, having remained undisturbed by sedimentary dynamics due to its elevated position, isolated from the dejection cone.” This is reinforced by the good set of dates it presents. However, this contrasts with the other excavated areas (Cones and Vestibule), where, as indicated, there have been post-depositional processes. For these areas, however, no radiocarbon dates are provided to indicate the validity of the stratigraphy. It is noted that it has been possible to correlate the different areas, assigning their levels to different periods. One way of doing this is through the correlation of dates, but these are not presented. Nor is there any mention of the study of archaeological materials from different areas that could clarify these assignments (for example, through publications).

Table S2. This is not a table; it is a figure that should be better related to the experimental part of the article, i.e., Figures 9-10 (see comments below).

Figures S1-7. I consider the inclusion of these figures in this part of the article to be very positive, as they help to better understand the location of the site and aspects related to the biometrics of the artifacts. Figure 5, inf should include a scale; Figures 2 and 7 should be translated into English.

Figure S8. The figures shown are essential for the correct study and interpretation of the pendants recovered from the Llonin cave. The acronyms for each piece (area of the cave, etc.) should be included, and perhaps a legend should be added to each of the pages included in this figure.

Figures S9-S10. The text on the experimental protocol carried out for the replicas made (working materials, taphonomy of the shells, execution and use time, etc.) should be included, although it should always be noted on which species the experimentation was carried out. It is considered appropriate to include it, given how detailed it is, although no experimentation has been carried out on bivalves or scaphopods. However, it is based on shells, which would support part of the study of the malacological objects documented in Llonín. However, the same is not done with other raw materials documented at the site: bone and mineral matter. In the case of teeth, photos of teeth are included, but it is not indicated to which animal they belong.

Reviewer #2: Thank you for your excellent work, very interesting and rigorously done. My suggestions mainly focus on the presentation of the results obtained from traceology. I hope you find my notes and suggestions useful and constructive.

**********

6. PLOS authors have the option to publish the peer review history of their article (what does this mean?). If published, this will include your full peer review and any attached files.

If you choose “no”, your identity will remain anonymous but your review may still be made public.

**Do you want your identity to be public for this peer review?** For information about this choice, including consent withdrawal, please see our Privacy Policy.

Reviewer #1: No

Reviewer #2: **Yes:** Leïla Hoareau

**********

[NOTE: If reviewer comments were submitted as an attachment file, they will be attached to this email and accessible via the submission site. Please log into your account, locate the manuscript record, and check for the action link "View Attachments". If this link does not appear, there are no attachment files.]

To ensure your figures meet our technical requirements, please review our figure guidelines: https://journals.plos.org/plosone/s/figures

You may also use PLOS’s free figure tool, NAAS, to help you prepare publication quality figures: https://journals.plos.org/plosone/s/figures#loc-tools-for-figure-preparation.

NAAS will assess whether your figures meet our technical requirements by comparing each figure against our figure specifications.

Attachment Submitted filename: Review_PONE-D-25-62247.docx

# Author comment: Author response to Decision Letter 1

**DOI:** 10.1371/journal.pone.0351170.r002

22 Apr 2026

Every point raised by the revisors have been answered on the documents attached to the submission "Response to reviewer 1" and "Response to reviewer 2"

Attachment Submitted filename: Response to Reviewer 2.pdf

# Peer review: Decision Letter 1

**DOI:** 10.1371/journal.pone.0351170.r003

**Authors:** Marco Peresani

24 May 2026

Identity and Mobility through Personal Ornaments in Upper Paleolithic Cantabrian Hunter-Gatherer Societies: insights from Llonín cave (Asturias, Spain)

PONE-D-25-62247R1

Dear Dr. Pérez-García de los Salmones,

We’re pleased to inform you that your manuscript has been judged scientifically suitable for publication and will be formally accepted for publication once it meets all outstanding technical requirements.

Within one week, you’ll receive an e-mail detailing the required amendments. When these have been addressed, you’ll receive a formal acceptance letter and your manuscript will be scheduled for publication.

An invoice will be generated when your article is formally accepted. Please note, if your institution has a publishing partnership with PLOS and your article meets the relevant criteria, all or part of your publication costs will be covered. Please make sure your user information is up-to-date by logging into Editorial Manager at Editorial Manager® and clicking the ‘Update My Information' link at the top of the page. For questions related to billing, please contact billing support.

If your institution or institutions have a press office, please notify them about your upcoming paper to help maximize its impact. If they’ll be preparing press materials, please inform our press team as soon as possible -- no later than 48 hours after receiving the formal acceptance. Your manuscript will remain under strict press embargo until 2 pm Eastern Time on the date of publication. For more information, please contact onepress@plos.org.

Kind regards,

Marco Peresani

Academic Editor

PLOS One

Reviewers' comments:

Reviewer's Responses to Questions

**Comments to the Author**

1. If the authors have adequately addressed your comments raised in a previous round of review and you feel that this manuscript is now acceptable for publication, you may indicate that here to bypass the “Comments to the Author” section, enter your conflict of interest statement in the “Confidential to Editor” section, and submit your "Accept" recommendation.

Reviewer #2: All comments have been addressed

**********

2. Is the manuscript technically sound, and do the data support the conclusions?

The manuscript must describe a technically sound piece of scientific research with data that supports the conclusions. Experiments must have been conducted rigorously, with appropriate controls, replication, and sample sizes. The conclusions must be drawn appropriately based on the data presented.

Reviewer #2: Yes

**********

3. Has the statistical analysis been performed appropriately and rigorously?

Reviewer #2: Yes

**********

4. Have the authors made all data underlying the findings in their manuscript fully available?

The PLOS Data policy requires authors to make all data underlying the findings described in their manuscript fully available without restriction, with rare exception (please refer to the Data Availability Statement in the manuscript PDF file). The data should be provided as part of the manuscript or its supporting information, or deposited to a public repository. For example, in addition to summary statistics, the data points behind means, medians and variance measures should be available. If there are restrictions on publicly sharing data—e.g. participant privacy or use of data from a third party—those must be specified.

Reviewer #2: Yes

**********

5. Is the manuscript presented in an intelligible fashion and written in standard English?

PLOS ONE does not copyedit accepted manuscripts, so the language in submitted articles must be clear, correct, and unambiguous. Any typographical or grammatical errors should be corrected at revision, so please note any specific errors here.

Reviewer #2: Yes

**********

6. Review Comments to the Author

Please use the space provided to explain your answers to the questions above. You may also include additional comments for the author, including concerns about dual publication, research ethics, or publication ethics. (Please upload your review as an attachment if it exceeds 20,000 characters)

Reviewer #2: The authors responded to all my comments and incorporated the necessary changes. I wish them all the best for their future work !

**********

7. PLOS authors have the option to publish the peer review history of their article (what does this mean?). If published, this will include your full peer review and any attached files.

If you choose “no”, your identity will remain anonymous but your review may still be made public.

**Do you want your identity to be public for this peer review?** For information about this choice, including consent withdrawal, please see our Privacy Policy.

Reviewer #2: **Yes:** Leïla Hoareau

**********

# Editor report: Acceptance letter

**DOI:** 10.1371/journal.pone.0351170.r004

**Authors:** Marco Peresani

PONE-D-25-62247R1

PLOS One

Dear Dr. Pérez-García de los Salmones,

I'm pleased to inform you that your manuscript has been deemed suitable for publication in PLOS One. Congratulations! Your manuscript is now being handed over to our production team.

At this stage, our production department will prepare your paper for publication. This includes ensuring the following:

* All references, tables, and figures are properly cited

* All relevant supporting information is included in the manuscript submission,

* There are no issues that prevent the paper from being properly typeset

You will receive further instructions from the production team, including instructions on how to review your proof when it is ready. Please keep in mind that we are working through a large volume of accepted articles, so please give us a few days to review your paper and let you know the next and final steps.

Lastly, if your institution or institutions have a press office, please let them know about your upcoming paper now to help maximize its impact. If they'll be preparing press materials, please inform our press team within the next 48 hours. Your manuscript will remain under strict press embargo until 2 pm Eastern Time on the date of publication. For more information, please contact onepress@plos.org.

You will receive an invoice from PLOS for your publication fee after your manuscript has reached the completed accept phase. If you receive an email requesting payment before acceptance or for any other service, this may be a phishing scheme. Learn how to identify phishing emails and protect your accounts at https://explore.plos.org/phishing.

If we can help with anything else, please email us at customercare@plos.org.

Thank you for submitting your work to PLOS ONE and supporting open access.

Kind regards,

PLOS ONE Editorial Office Staff

on behalf of

Dr. Marco Peresani

Academic Editor

PLOS One
